# Tunable Self-Referenced Molecular Thermometers via Manipulation of Dual Emission in Platinum(II) Pyridinedipyrrolide Complexes

**DOI:** 10.1021/acsami.3c19226

**Published:** 2024-02-23

**Authors:** Andreas Russegger, Susanne M. Fischer, Angela C. Debruyne, Helmar Wiltsche, A. Daniel Boese, Ruslan I. Dmitriev, Sergey M. Borisov

**Affiliations:** † Institute of Analytical Chemistry and Food Chemistry, Graz University of Technology, Stremayrgasse 9, Graz 8010, Austria; ‡ Physical and Theoretical Chemistry, Institute of Chemistry, University of Graz, Heinrichstrasse 28/IV, Graz 8010, Austria; § Tissue Engineering and Biomaterials Group, Department of Human Structure and Repair, Faculty of Medical and Health Sciences, Ghent University, C. Heymanslaan 10, Ghent 9000, Belgium; ∥ Ghent Light Microscopy Core, Ghent University, Ghent 9000, Belgium

**Keywords:** TADF, phosphorescence, ratiometric, temperature, sensor, imaging

## Abstract

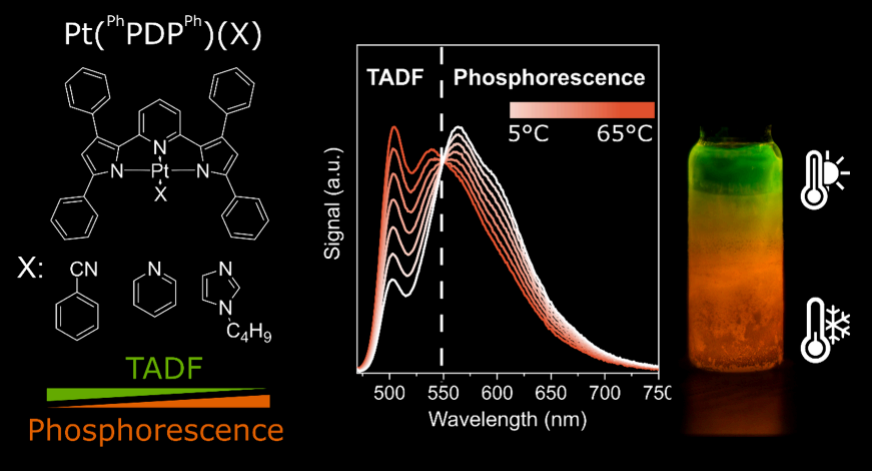

Optical temperature sensors based on self-referenced readout schemes such as the emission ratio and the decay time are crucial for a wide range of applications, with the former often preferred due to simplicity of instrumentation. This work describes a new group of dually emitting dyes, platinum(II) pincer complexes, that can be used directly for ratiometric temperature sensing without an additional reference material. They consist of Pt(II) metal center surrounded by a pyridinedipyrrolide ligand (PDP) and a terminal ligand (benzonitrile, pyridine, 1-butylimidazol or carbon monoxide). Upon excitation with blue light, these complexes exhibit green to orange emission, with quantum yields in anoxic toluene at 25 °C ranging from 13% to 86% and decay times spanning from 8.5 to 97 μs. The emission is attributed to simultaneous thermally activated delayed fluorescence (TADF) and phosphorescence processes on the basis of photophysical investigations and DFT calculations. Rather uniquely, simple manipulations in substituents of the PDP ligand and alteration of the terminal ligand allow fine-tuning of the ratio between TADF and phosphorescence from almost 100% TADF emission (**Pt(**^**Mes**^**PDP**^**C6F5**^**(BN)**) to over 80% of phosphorescence (**Pt(**^**Ph**^**PDP**^**Ph**^**(BuIm)**). Apart from ratiometric capabilities, the complexes also are useful as decay time-based temperature indicators with temperature coefficients exceeding 1.5% K^–1^ in most cases. Immobilization of the dyes into oxygen-impermeable polyacrylonitrile produces temperature sensing materials that can be read out with an ordinary RGB camera or a smartphone. In addition, **Pt(**^**Ph**^**PDP**^**Ph**^**)Py** can be incorporated into biocompatible RL100 nanoparticles suitable for cellular nanothermometry, as we demonstrate with temperature measurements in multicellular colon cancer spheroids.

## 1 Introduction

In the past decades luminescent optical sensors became indispensable analytical tools in research and industry. With luminescence sensors, a wide range of analytes can be measured such as oxygen,^1^ pH,^2^ carbon dioxide,^3^ ammonia,^4^ and ions,^5^ to mention only a few.

Temperature represents another parameter of interest, also due to the fact that any sensor (including optical) shows temperature-dependent behavior. Although it can be conveniently accessed with conventional tools like resistance thermometers, optical read-out offers advantages in certain scenarios such as measurements in strong electromagnetic fields, mapping of temperature distribution (e.g., with temperature-sensitive paints), or studies in particularly small objects like microfluidic chips or live cells.^6,7^

Many optical thermometers reported so far can be classified into six readout groups depending on which luminescence property exhibits temperature-dependent behavior: (1) emission intensity, (2) spectral positions of absorption and emission bands, (3) emission bandwidth, (4) anisotropy, (5) luminescence decay time, and (6) relative intensity between two emission bands (ratiometric systems).^8^ Optical thermometers of the last two types are particularly interesting because of their self-referencing character. In fact, they can compensate for fluctuations in the intensity of the excitation light, sensitivity of the photodetector, inhomogeneous distribution of the luminescent probe and, in case of decay time read-out, photobleaching.

Ratiometric optical thermometers rely on measuring the intensity ratio between two different emission signals and benefit from simplicity of needed instrumentation (e.g., two filters or RGB channels). Depending on the origin of the two emission signals, three different groups can be distinguished: (a) two luminescent dyes, at least one of which shows strong response to temperature, (b) two indicators interacting by energy transfer with each other, the magnitude of which is temperature-dependent and (c) one indicator with two excited luminescent states in thermal equilibrium.^9,10^ Optical thermometers based on two luminophores (groups (a) and (b)) are the most common systems and numerous materials have been reported in various designs (e.g., metal–organic frameworks,^11,12^ lanthanide coordination polymers,^13^ upconversion nanoparticles,^14−16^ dye-doped nanoparticles,^17−19^ polymer-based materials,^20−22^ and quantum dots^23^). However, application of these systems can be challenged by increased complexity in sensor design, drift due to instability of the indicator or the reference material and change of the luminescence ratio in the presence of quenchers that affect the individual emitters to a different extent. Therefore, indicators that exhibit two temperature-dependent emission bands due to thermal equilibrium between two excited states (group (c)) are of particular interest. However, temperature sensing materials of this type are rare and mostly include lanthanide nanoparticles,^24,25^ quantum dots,^26,27^ copper(I) iodide clusters,^28^ polymer dots,^29^ and molecular probes such as porphyrin dimers,^30^ corannulenes,^31^ and small organic molecules of other classes.^32−34^

A particularly interesting group of dual emitters features simultaneous thermally activated delayed fluorescence (TADF) and phosphorescence. So far, such emission has only been observed in a small number of compounds, such as organic molecules,^35−37^ copper(I) complexes,^38^ and complexes of platinum group metals.^39−42^ We previously showed that also metalobenzoporphyrins can exhibit simultaneous TADF and phosphorescence which can be utilized not only for temperature sensing but also to measure temperature and oxygen at the same time.^43,44^

**Figure 1:**
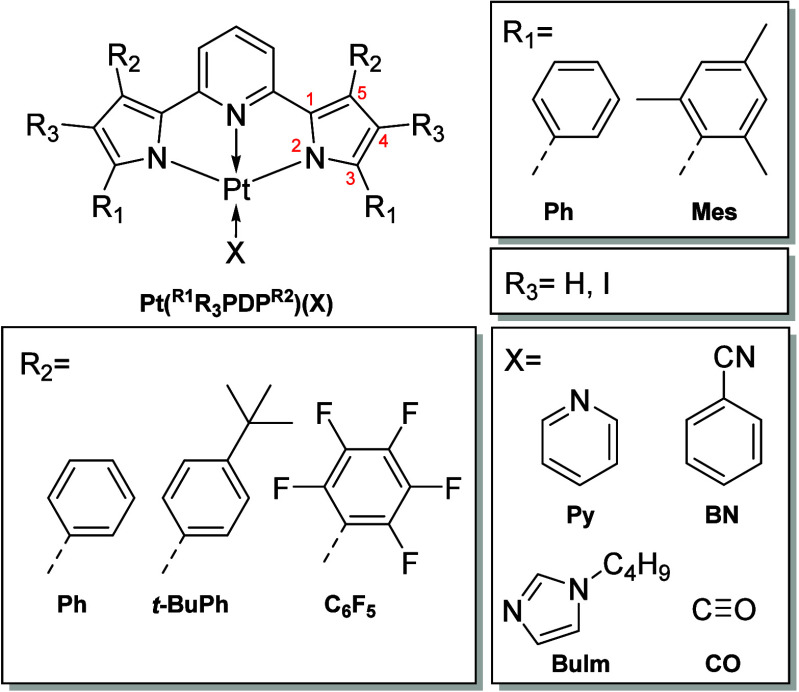
Structures of synthesized Pt(^R1^R_3_PDP^R2^)(X) complexes.

In this work, we report a new group of platinum(II) pyridinedipyrrolide complexes (Figure 1) that simultaneously emit in the green and orange regions of the electromagnetic spectrum. The complexes can be used as optical thermometers and the readout mode can be varied between lifetime-based and ratiometric by simply exchanging the substituents on the pyridinedipyrrolide (PDP) core or by exchanging the terminal ligand. The complexes for ratiometric readout are particularly interesting because, due to their spectral properties, inexpensive RGB cameras^45^ or microscopes can be used.

## 2 Results and Discussion

### Design and Synthesis of the Emitters

The new square-planar Pt(II) complexes consist of a tridentate PDP pincer ligand and a terminal ligand and thus differ significantly from reported zirconium(IV) complexes with the same pyridinedipyrrolide ligand (Zr(PDP)_2_).^46−49^ Although similar Pt(II) complexes have been synthesized in the past, their photophysical properties were either not investigated or the complexes solely emitted phosphorescence.^50−52^ Because of the small singlet–triplet energy gap in such complexes it was of considerable interest to investigate how the photophysical properties of the Pt(II) complexes are influenced by comparably small structural changes in the pincer ligand and by the nature of the terminal ligand. The modification possibilities included (i) sterically hindered mesityl (Mes) group instead of phenyl (Ph) group as R_1_ (Figure 1); (ii) introduction of electron-donating (*t*-Bu) or electron-withdrawing (F) substituents in the phenyl ring as R_2_; (iii) substitution of H by heavy atom (I) in R_3_; and (iv) varying the terminal ligand X. Overall, ten different complexes have been prepared.

**Scheme 1:**
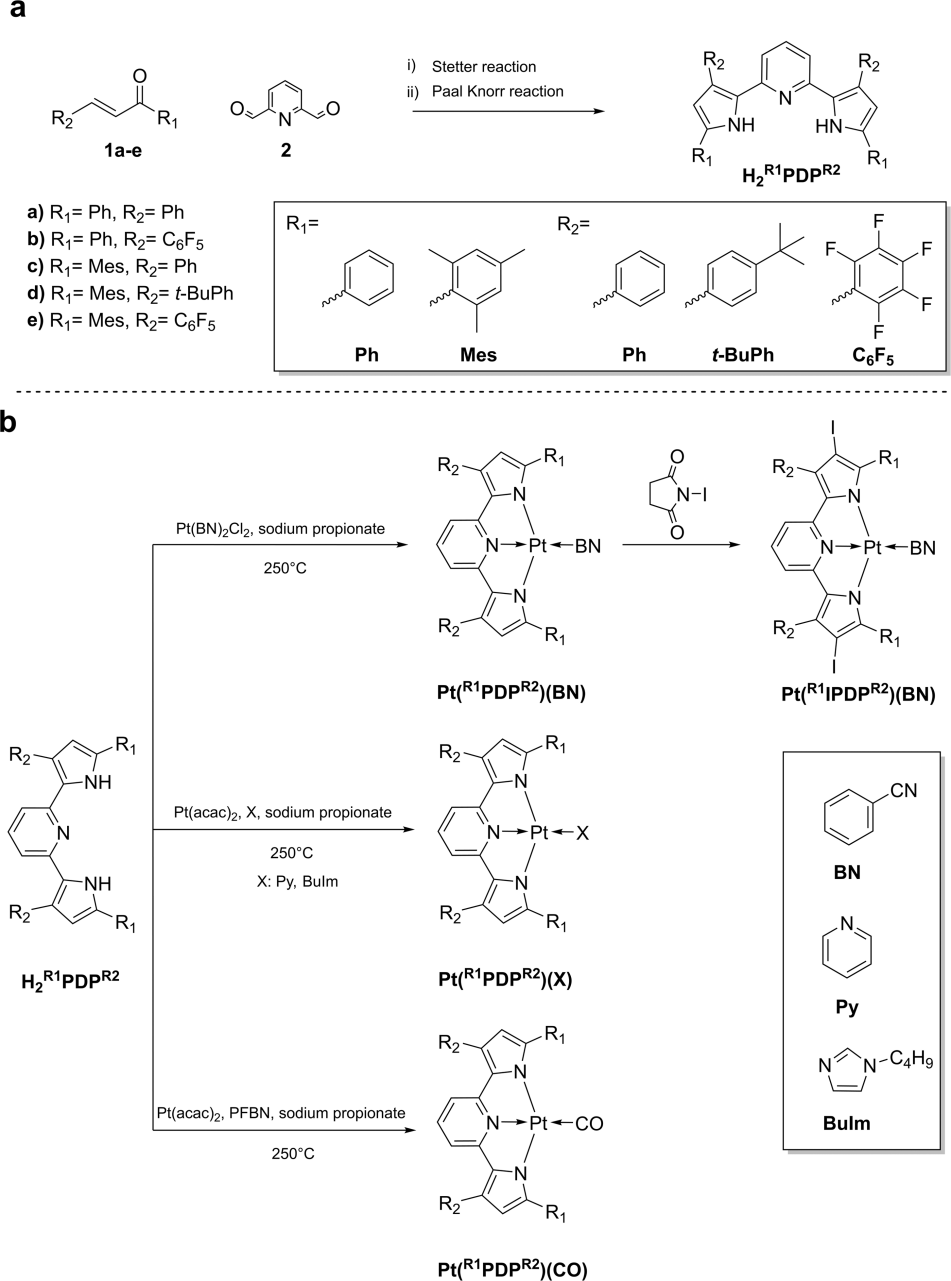
Synthesis of H_2_^R1^PDP^R2^-Ligands (a) and the Platinum(II) Complexes (b)

The synthesis of H_2_^R2^PDP^R1^ is straightforward (Scheme 1a) and follows the previously reported protocols.^46,47,49,53^ The pincer ligand is synthesized in a two-step, one-pot reaction starting from chalcone derivatives utilizing Stetter and Paal–Knorr reactions.

The Pt(II) complexes are obtained by reacting H_2_^R2^PDP^R1^ with a respective Pt(II) precursor in hot 1,2-dichlorobenzene (250 °C) in the presence of sodium propionate as proton scavenger (Scheme 1b). Importantly, the nature of the Pt(II) precursor determines which terminal ligand is coordinated to the metal center. *Cis*-bis(benzonitrile)dichloroplatinum(II) (Pt(BN)_2_Cl_2_) was used to obtain complexes with terminal benzonitrile (BN). Quite surprisingly we found it impossible to exchange the terminal ligand by stirring the BN complex with excess of another ligand in 1,2-dichlorobenzene at 250 °C. The complexes with pyridine (Py) or 1-butylimidazol (BuIm) as terminal ligands were prepared from Pt(II) acetylacetonate (Pt(acac)_2_) and an excess of the respective terminal ligand. Here, the weaker acetylacetone ligands were replaced *in situ* by the stronger coordinating Py or BuIm ligand. Rather unexpectedly, the complex with carbon monoxide (CO) terminal ligand was obtained as the main product by using Pt(acac)_2_ in combination with 2,3,4,5,6-pentafluorobenzonitrile (PFBN). We observed the formation of the same complex when Pt(BN)_2_Cl_2_ was used as a precursor, but only as a side product. Finally, in order to access the effect of additional heavy atoms in the pyrrole rings, **Pt(**^**Ph**^**PDP**^**Ph**^**)(BN)** and **Pt(**^**Mes**^**PDP**^**C6F5**^**)(BN)** were reacted with *N*-iodosuccinimide.

### Photophysical Properties

**Figure 2:**
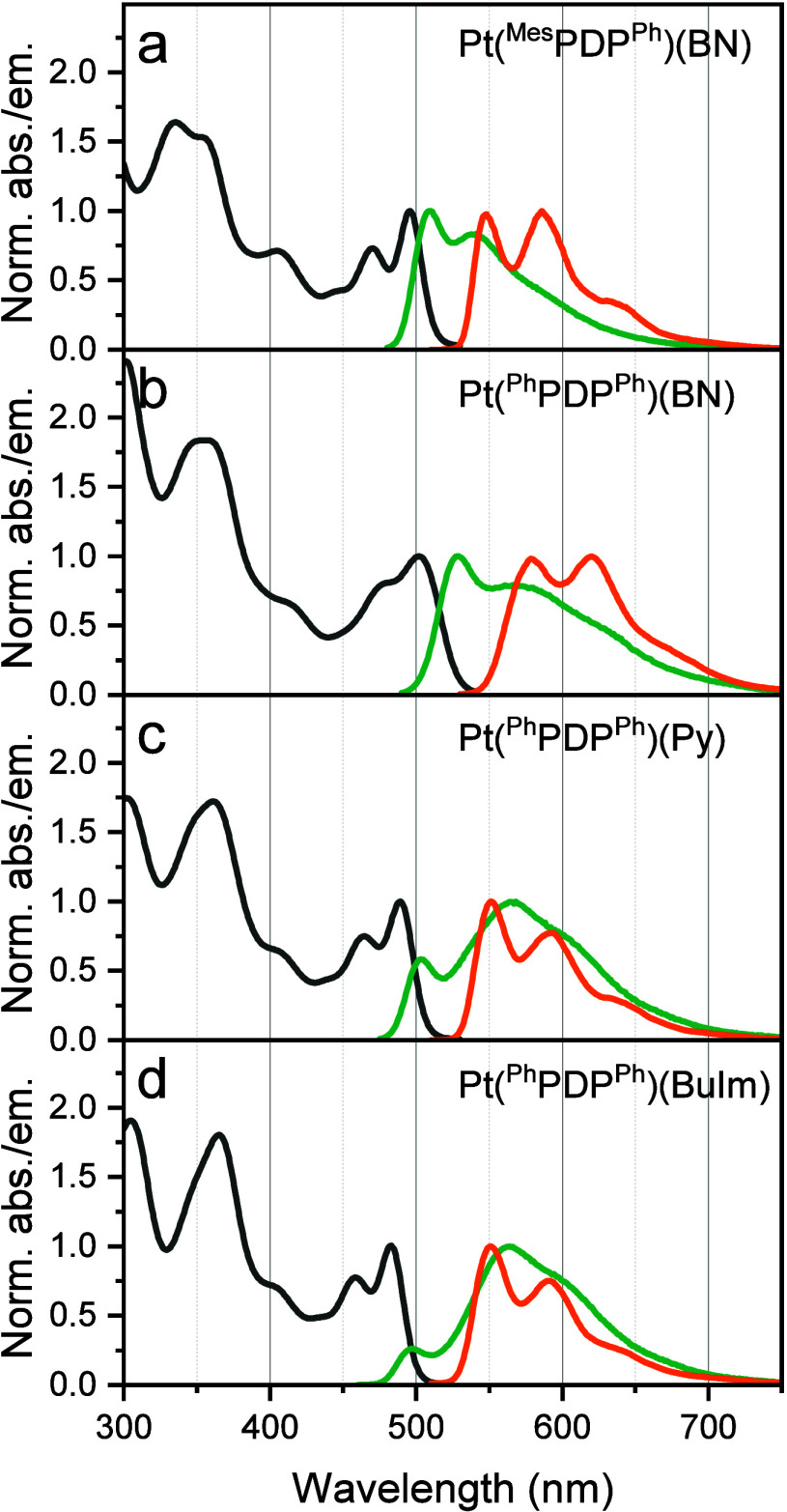
Normalized absorption spectra (in toluene, black lines), emission spectra at room temperature (in anoxic toluene, green lines) and at 77 K (in toluene/THF (4:6 v/v), orange lines) of (a) **Pt(**^**Mes**^**PDP**^**Ph**^**)(BN)**, (b) **Pt(**^**Ph**^**PDP**^**Ph**^**)(BN)**, (c) **Pt(**^**Ph**^**PDP**^**Ph**^**)(Py)**, and (d) **Pt(**^**Ph**^**PDP**^**Ph**^**)(BuIm)**.

The absorption of the complexes spans from UV to the blue part of the electromagnetic spectrum (Figure 2, Figure S1 (Supporting Information), Table 1). Similar to previously reported Zr(PDP)_2_ chelates^46,47^ the shape and the position of the absorption bands in the Pt(II) complexes are barely affected by substituents in the PDP ligand. The same is observed for the terminal ligand. Thus, the complexes are compatible with common light sources such as blue LEDs (450–485 nm) or argon laser (488 nm). In stark contrast to other complexes, **Pt(**^**Ph**^**PDP**^**Ph**^**)(CO)** shows a red-shifted absorption spectrum with an absorption maximum at 525 nm.

**Table 1:** Photophysical Properties of Pt(II) Complexes in Toluene at 25 °C

Complex	λ_abs,max_/ε (nm/M^–1^ cm^–1^)	λ_em_ (nm)	λ_em,77K_^a^ (nm)	Δ*E*_ST_^b^(eV)	τ (μs)	Φ_rel_^d^	*k*_r_ × 10^–2^(s^–1^)	*k*_nr_ × 10^–2^(s^–1^)
**Pt(**^**Ph**^**PDP**^**Ph**^**)(BN)**	502/13 700	529, 568	579, 620	0.21	80	0.30	38	88
**Pt(**^**Ph**^**PDP**^**Ph**^**)(Py)**	489/13 300	503, 566	551, 592	0.23	97	0.45	46	57
**Pt(**^**Ph**^**PDP**^**Ph**^**)(BuIm)**	483/13 300	497, 564	551, 590	0.25	64	0.28	44	110
**Pt(**^**Ph**^**PDP**^**Ph**^**)(CO)**	525/14 200	551	613	0.33	40^c^	<0.01	<2.5	250
**Pt(**^**Ph**^**IPDP**^**Ph**^**)(BN)**	494/12 600	513, 541	541, 578	0.18	8.8^c^	0.13	150	990
**Pt(**^**Ph**^**PDP**^**C6F5**^**)(BN)**	492/10 700	514, 552	561, 596	0.19	57	0.44	77	98
**Pt(**^**Mes**^**PDP**^**Ph**^**)(BN)**	496/15 700	509, 541	548, 586	0.20	47	0.47	100	110
**Pt(**^**Mes**^**PDP**^*t***-BuPh**^)(BN)	496/15 200	511, 541	548, 587	0.20	77	0.73	95	35
**Pt(**^**Mes**^**PDP**^**C6F5**^**)(BN)**	488/13 200	501, 532	532, 570	0.18	36	0.86	240	39
**Pt(**^**Mes**^**IPDP**^**C6F5**^**)(BN)**	491/10 100	504, 533	533, 571	0.19	8.5	0.85	1000	180

a Emission maxima measured in a mixture of toluene/THF (4:6 v/v) at 77 K.

b Calculated from the onset of the emission bands.

c Biexponential decay, here the average lifetime is given.

d Relative to fluorescein in 0.1 M NaOH (Φ = 0.92).^54^

Under anoxic conditions, the complexes are brightly emissive in the green and orange regions of the electromagnetic spectrum (Figure 2, Figure S1 (Supporting Information), Table 1). **Pt(**^**Ph**^**PDP**^**Ph**^**)(CO)**, again represents a notable exception with emission quantum yield below 1%. Substitution of the PDP ligand has little effect on the shape of the emission spectrum. In contrast, the substitution of the terminal ligands from BN to Py or BuIm significantly changes the shape of the emission spectrum (Figure 2). In fact, the emission between 480 and 500 nm decreases in the order **Pt(**^**Ph**^**PDP**^**Ph**^**)(BN)** > **Pt(**^**Ph**^**PDP**^**Ph**^**)(Py)** > **Pt(**^**Ph**^**PDP**^**Ph**^**)(BuIm)** which is accompanied by appearance of the second emission maximum above 550 nm. This behavior is attributed to a change in the emission type from TADF to phosphorescence showing emission maxima at about 500 and 550 nm, respectively.

At room temperature, the emission of **Pt(**^**Ph**^**PDP**^**Ph**^**)(BN)** is mainly of TADF type with only a small contribution of phosphorescence indicated by a shoulder in the emission spectrum. For **Pt(**^**Ph**^**PDP**^**Ph**^**)(Py)** and especially for **Pt(**^**Ph**^**PDP**^**Ph**^**)(BuIm)** the TADF emission is much weaker than the phosphorescence compared to **Pt(**^**Ph**^**PDP**^**Ph**^**)(BN)**. This behavior is in excellent correlation with the energy gap between the S_1_ and T_1_ states (Δ*E*_*ST*_) that increases from (Table 1). This can be explained by enhanced HOMO/LUMO overlap in these complexes. In contrast, Δ*E*_*ST*_ decreases at the transition from **Pt(**^**Ph**^**PDP**^**Ph**^**)(BN)** to **Pt(**^**Mes**^**PDP**^**Ph**^**)(BN)** (0.21 and 0.20 eV, respectively) due to a better delocalization of the HOMO and LUMO orbitals, leading to a larger fraction of TADF (Figure 2a and b). A further reduction of Δ*E*_*ST*_ and thus an almost pure TADF emission can be achieved by introducing iodine into the pyrrole rings. For **Pt(**^**Ph**^**IPDP**^**Ph**^**)(BN)** and **Pt(**^**Mes**^**IPDP**^**C6F5**^**)(BN)**, the lowest Δ*E*_ST_ values were achieved with 0.18 and 0.19 eV respectively. To the best of our knowledge, such conversion of TADF to phosphorescence with help of comparably small structural modifications is unprecedented in literature. In general, Δ*E*_*ST*_ values ranging from 0.18 to 0.33 eV are comparable to those determined for other compounds with dual TADF and phosphorescence.^38,39,41^

**Figure 3:**
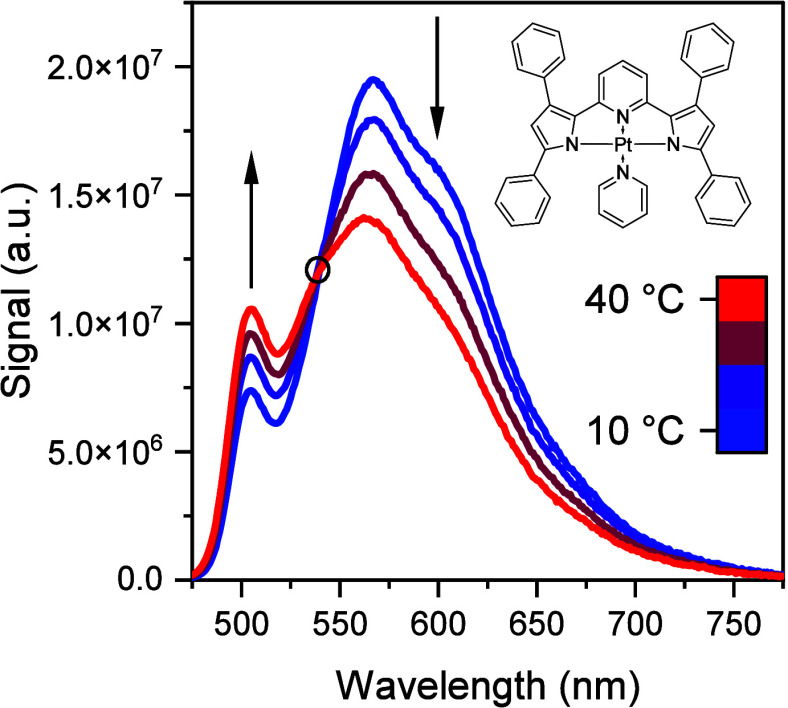
Emission spectra (λ_exc_ = 455 nm) of **Pt(**^**Ph**^**PDP**^**Ph**^**)(Py)** between 10 and 40 °C measured in toluene under anoxic conditions.

The origin of the emission was confirmed by measurements at 77 K. At low temperatures, the repopulation of the S_1_ state from the T_1_ state is prevented and only the red-shifted phosphorescence can be observed in all cases (Figure 2 and Figure S1; Supporting Information). Vice versa, the dyes showing comparably strong phosphorescence at room temperature show characteristic spectral changes with temperature (Figure 3 as exemplified for **Pt(**^**Ph**^**PDP**^**Ph**^**)(Py)**). As the temperature increases, TADF gets enhanced and the phosphorescence intensity decreases. Isosbestic point (marked with a black circle) indicates that the emission shifts between the two electronic transitions.

For the complexes **Pt(**^**Mes**^**PDP**^**Ph**^**)(BN)** and **Pt(**^**Ph**^**PDP**^**Ph**^**)(Py)** the influence of solvent polarity was investigated. Both complexes show a slight hypsochromic shift of the absorption spectra and slight bathochromic shift of the emission when the polarity is increased (Figure S2, Supporting Information), whereby the solvatochromic effect is more pronounced for **Pt(**^**Ph**^**PDP**^**Ph**^**)(Py)**. In addition, **Pt(**^**Ph**^**PDP**^**Ph**^**)(Py)** shows significant change in the TADF/phosphorescence ratio with lower contribution of TADF in more polar solvents.

Luminescence quantum yields (Φ) strongly depend on substituents. Substitution of phenyl group by mesityl at the 3-position improves quantum yields and the pentafluorophenyl (C_6_F_5_) group at the 5-position appears to be also beneficial. Interestingly, iodination of **Pt(**^**Ph**^**PDP**^**Ph**^**)(BN)** yielding **Pt(**^**Ph**^**IPDP**^**Ph**^**)(BN)** leads to significant reduction in the quantum yield but no change in the quantum yield in case of **Pt(**^**Mes**^**PDP**^**C6F5**^**)(BN)**. As mentioned above, **Pt(**^**Ph**^**PDP**^**Ph**^**)(CO)** is significantly different from the other complexes and shows very low Φ. Although the reason for this is currently unknown, we note that this compound mainly shows TADF (Figure S1, Supporting Information) despite a large Δ*E*_ST_ (0.33 eV). This behavior might be explained by the higher contribution of the heavy platinum metal center to the LUMO and thus by a higher exchange rate between the S_1_ and T_1_ states.

Except for **Pt(**^**Ph**^**IPDP**^**Ph**^**)(BN)** and **Pt(**^**Ph**^**PDP**^**Ph**^**)(CO)** all the complexes show monoexponential decay (Figure S3, Supporting Information) and the decay times (τ) for all platinum(II) complexes are below 100 μs at 25 °C. It should be mentioned that the measurement of the decay time at the wavelengths corresponding to the TADF and phosphorescence gives identical values, which means that both emissions are in thermal equilibrium. This was also observed for other dual-TADF/phosphorescence emitters.^39,43^ Substitution with an electron-withdrawing C_6_F_5_-group at the 5-position, Mes-groups at the 3-position and iodine at the 4-position leads to shorter decay times in comparison to **Pt(**^**Ph**^**PDP**^**Ph**^**)(BN)** (Table 1). These substitutions show additive effect so that the decay time for **Pt(**^**Mes**^**IPDP**^**C6F5**^**)(BN)** is about 1 order of magnitude lower than for **Pt(**^**Ph**^**PDP**^**Ph**^**)(BN)**. The variation of terminal ligands also affects the decay times. The longest lifetimes were observed for **Pt(**^**Ph**^**PDP**^**Ph**^**)(Py)** and the shortest for **Pt(**^**Ph**^**PDP**^**Ph**^**)(CO)**.

Photostability of the new dyes was accessed by irradiating air-saturated solutions in toluene with a high-power light source (metal halide lamp). The decrease in dye concentration was measured via UV–vis spectroscopy (Figure S4, Figure S5, Supporting Information). Tetramethylrhodamine ethyl ester perchlorate (TMR) dissolved in water, that was reported to have good photostability,^55^ was selected as a reference dye. Quite surprisingly, photostability of all Pt(II) complexes is significantly lower than that of TMR, which is in stark contrast to the results obtained previously for Zr(IV) bis-pyridinedipyrrolide complexes that were found to be even more photostable than TMR.^34^ In the Zr(IV) complexes, the chromophore might be better protected since two bulky ligands are assembled around the central metal in octahedral configuration. Among the Pt(II) complexes, the lowest photostabilities were observed for **Pt(**^**Ph**^**PDP**^**Ph**^**)(BN)**, **Pt(**^**Mes**^**PDP**^**Ph**^**)(BN)** and for iodine substituted complexes **Pt(**^**Ph**^**IPDP**^**Ph**^**)(BN)** and **Pt(**^**Mes**^**IPDP**^**C6F5**^**)(BN)**. Substitution with an electron withdrawing C_6_F_5_ group at the 5-position and particularly with terminal ligands like Py, BuIm or CO instead of BN significantly enhances the photostability.

### DFT and TDDFT Calculations

**Figure 4:**
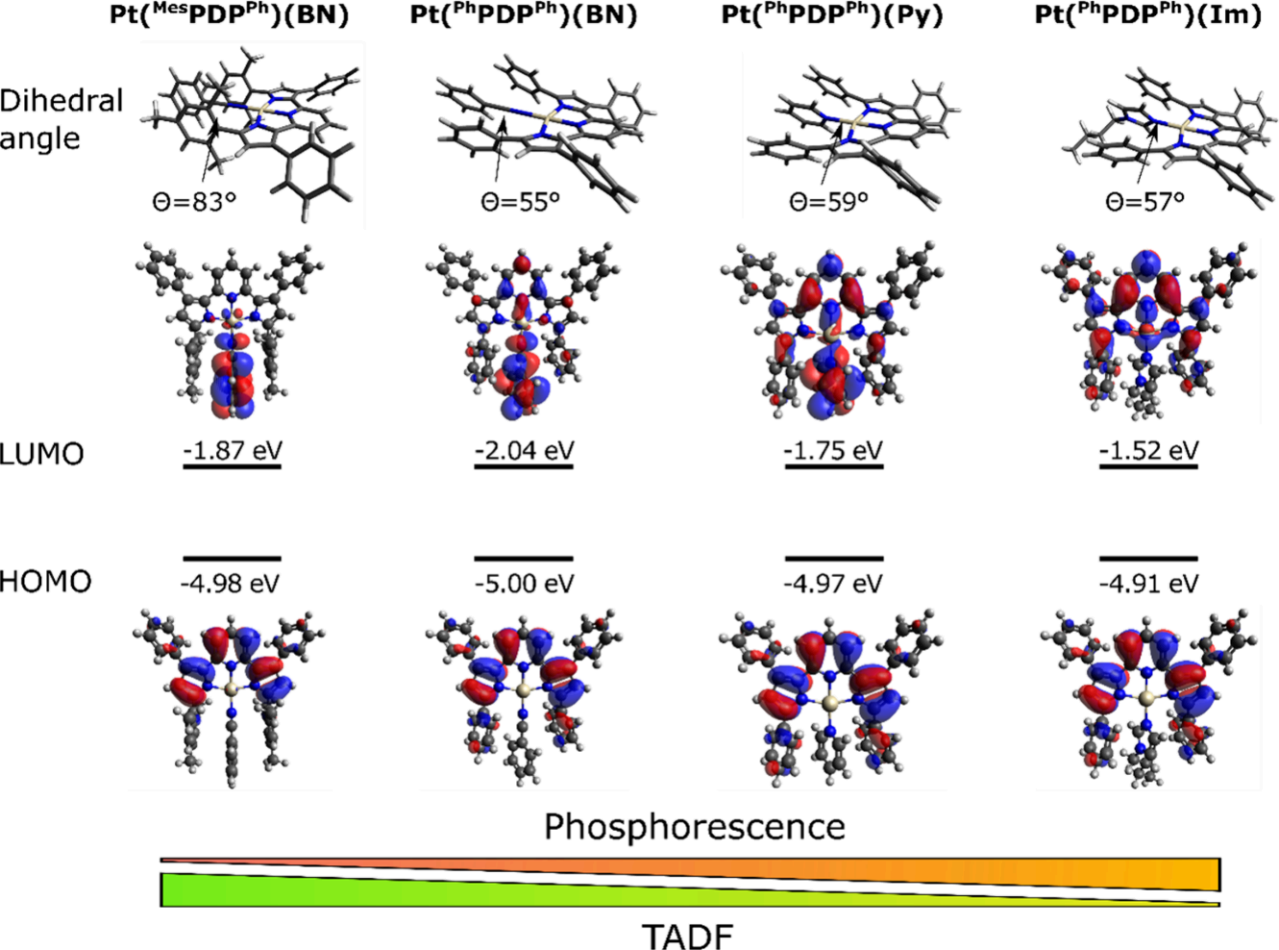
Perspective drawings of the optimized ground state (*S*_0_) geometries with marked dihedral angle (θ) between PDP ligand and terminal ligand and HOMO and LUMO of **Pt(**^**Mes**^**PDP**^**Ph**^**)(BN)**, **Pt(**^**Ph**^**PDP**^**Ph**^**)(BN)**, **Pt(**^**Ph**^**PDP**^**Ph**^**)(Py)**, and **Pt(**^**Ph**^**PDP**^**Ph**^**)(BuIm)**.

To gain insights into the influence of the modification of the ligand on the photophysical properties, density functional theory (DFT) calculations have been carried out at the B3LYP/def2-TZVP level. The results are summarized in Table S1 (Supporting Information) and a graphical visualization of the frontal molecular orbitals is given in Figure 4 and Figure S6 of the Supporting Information.

It was found that the localization of the highest occupied molecular orbitals (HOMO) is hardly affected by varying the PDP substituents. In all Pt(II) complexes, the HOMO is mainly located on the pyridine and pyrrole rings of the PDP ligand and has an energy between −4.91 eV and −5.41 eV. However, for complexes containing C_6_F_5_-subsituents at the 5-position of the pyrrole ring, d-orbitals of the Pt(II) metal center also contribute to the HOMO leading to a stabilization of it (−5.31 eV and −5.00 eV for **Pt(**^**Ph**^**PDP**^**C6F5**^**)(BN)** and **Pt(**^**Ph**^**PDP**^**Ph**^**)(BN)**, respectively, and −5.31 eV and −4.98 eV for **Pt(**^**Mes**^**PDP**^**C6F5**^**)(BN)** and **Pt(**^**Mes**^**PDP**^**Ph**^**)(BN)**, respectively) which can be rationalized with the electron-withdrawing properties of the C_6_F_5_-substituent. Similar effects are observed for complexes with iodine at the 4-position of the pyrrole ring (**Pt(**^**Ph**^**IPDP**^**Ph**^**)(BN)** and **Pt(**^**Mes**^**IPDP**^**C6F5**^**)(BN)**, respectively).

The localization of the LUMOs strongly depends on the substituent of the PDP ligand and the terminal ligand which is in sharp contrast to the qualitative observations made for the HOMOs. For example, all complexes with Mes-groups at the 3-position exhibit a delocalization of the LUMO over the terminal BN ligand and the platinum atom. In these complexes, the terminal ligand is almost perpendicular to the PDP ligand (dihedral angle (θ) between 82 and 90°) due to the bulky Mes-groups leading to a complete spatial separation of the frontal molecular orbitals. In contrast, by exchanging the Mes-groups to Ph-groups, the terminal ligands are less rotated (θ between 55° and 74°) allowing for an overlap of the frontal orbitals. The LUMO energies range from −1.52 eV to −2.17 eV. Similar to the HOMOs, the introduction of C_6_F_5_-substituents stabilizes the LUMOs and leads to lower energies compared to the Ph-substituted complexes (−2.17 eV and −2.04 eV for **Pt(**^**Ph**^**PDP**^**C6F5**^**)(BN)** and **Pt(**^**Ph**^**PDP**^**Ph**^**)(BN)**, respectively, and −2.01 eV and −1.87 eV for **Pt(**^**Mes**^**PDP**^**C6F5**^**)(BN)** and **Pt(**^**Mes**^**PDP**^**Ph**^**)(BN)**, respectively). Additionally, the terminal ligand plays an important role in the localization of the LUMO (Figure 4). While the LUMO of **Pt(**^**Ph**^**PDP**^**Ph**^**)(BN)** and **Pt(**^**Ph**^**PDP**^**Ph**^**)(Py)** is located both on the PDP ligand and on the terminal ligand, in **Pt(**^**Ph**^**PDP**^**Ph**^**)(BuIm)** it is localized only on the PDP ligand.

To further support the experimental observations, excitation energies were calculated using time-dependent density functional theory (TDDFT) using the above-mentioned method. For most complexes we found the lowest energy singlet excitation to be of HOMO → LUMO character while for the fluorine containing complexes **Pt(**^**Mes**^**PDP**^**C6F5**^**)(BN)** and **Pt(**^**Mes**^**IPDP**^**C6F5**^**)(BN)** the singlet excitation transition occurs between HOMO → LUMO+1. The lowest energy triplet excitation is mostly of mixed character, with the HOMO → LUMO+1 transition as the dominant one except for the two above-mentioned complexes. After calculation of the vertical excitation energies (Table S1, Supporting Information), deviations from experimental values could be observed. Therefore, geometry optimization of both S_1_ and T_1_ states was carried out, and the previously reported approach of calculating excitation energies by utilizing ground-state geometries could not be applied in this case.^39−41,48^ Here, the excited state structure will be distorted by the rotation of the dihedral angle, changing the excitation energy in turn. Calculated Δ*E*_ST_ between the optimized S_1_ and T_1_ states are reported in Table S1 (Supporting Info). Generally, TDDFT calculations are in line with the experimentally observed trend. The singlet–triplet energy gap increases from 0.13 to 0.44 eV in the order of **Pt(**^**Mes**^**PDP**^**Ph**^**)(BN)** < **Pt(**^**Ph**^**PDP**^**Ph**^**)(BN)** < **Pt(**^**Ph**^**PDP**^**Ph**^**)(Py)** < **Pt(**^**Ph**^**PDP**^**Ph**^**)(BuIm)** which was also observed experimentally. The lowest singlet–triplet energy gap is observed for **Pt(**^**Mes**^**PDP**^**Ph**^**)(BN)** also showing the biggest spatial separation of frontal orbitals and largest dihedral angle between the donor and acceptor ligands among the four complexes. The opposite was observed for the complex with the largest gap, **Pt(**^**Ph**^**PDP**^**Ph**^**)(BuIm)**. Therefore, it can be concluded that efficient decoupling of the frontier orbitals leads to smaller singlet–triplet energy gaps and mainly TADF emission which is in line with previously reported data.^39,56^

The method used here can accurately reflect the experimentally observed trends except for the complexes containing heavy atoms (F, I) where the obtained energies are generally overestimated and the complex **Pt(**^**Ph**^**PDP**^**Ph**^**)(CO)** where the influence of the loosely bound CO molecule cannot be described properly. Thus, with TDDFT we obtain some qualitative agreement with the experiment without having to revert to post-Hartree–Fock methods.

### Properties of Polystyrene-Immobilized Dyes

Further evaluation of photophysical and temperature-sensing properties was performed for dyes (0.5 wt %) embedded in polystyrene (PS) that was used as a model substrate. In order to avoid oxygen quenching the materials were studied under oxygen-free conditions. Immobilization in rigid PS matrix does not significantly affect the emission spectra (at room temperature and 77 K, Figure S7, Supporting Information) but was found to enhance the luminescence quantum yields and result in longer decay times (Table 2). Similar to the solution, the complexes in PS exhibit a monoexponential decay with the exception of **Pt(**^**Ph**^**IPDP**^**Ph**^**)(BN)** and **Pt(**^**Ph**^**PDP**^**Ph**^**)(CO)** (Figure S8, Supporting Information).

**Table 2:** Photophysical Properties of Pt(II) Complexes in Rigid Polystyrene Matrix under Anoxic Conditions at 25 °C

Complex	λ_em_ (nm)	λ_TADF,max_/λ_phos,max_^a^ (nm)	Δ*E*_ST_^b^ (eV)	τ(μs)	Φ_abs_^c^	dτ d*T*^–1^/dRatio d*T*^–1^(% K^–1^)
**Pt(**^**Ph**^**PDP**^**Ph**^**)(BN)**	526, 570	526/582	0.24	124	0.38 (0.52/0.48)	–1.4/1.4
**Pt(**^**Ph**^**PDP**^**Ph**^**)(Py)**	503, 563	504/560	0.24	135	0.68 (0.31/0.69)	–0.9/2.1
**Pt(**^**Ph**^**PDP**^**Ph**^**)(BuIm)**	497, 561	497/558	0.24	123	0.65 (0.17/0.83)	–0.6/2.7
**Pt(**^**Ph**^**PDP**^**Ph**^**)(CO)**	541, 571^d^	541/614	0.23	57^e^	0.03 (0.97/0.03)	–2.8/-
**Pt(**^**Ph**^**IPDP**^**Ph**^**)(BN)**	511, 539	511/547	0.17	25^e^	0.37 (0.82/0.18)	–2.4/0.4
**Pt(**^**Ph**^**PDP**^**C6F5**^**)(BN)**	513, 550	513/559	0.19	74	0.67 (0.60/0.40)	–1.4/1.2
**Pt(**^**Mes**^**PDP**^**Ph**^**)(BN)**	508, 539	509/552	0.20	78	0.60 (0.75/0.25)	–1.7/0.6
**Pt(**^**Mes**^**PDP**^*t***-BuPh**^)(BN)	509, 539	508/559	0.20	86	0.58 (0.73/0.27)	–1.7/0.8
**Pt(**^**Mes**^**PDP**^**C6F5**^**)(BN)**	499, 530	499/533	0.17	36	0.77 (0.81/0.19)	–1.6/0.5
**Pt(**^**Mes**^**IPDP**^**C6F5**^**)(BN)**	502, 530	502/527	0.14	9.5	0.85 (0.97/0.03)	–1.6/0.2

a TADF and phosphorescence emission maximum (Figure S7, Supporting Information).

b Calculated from the onset of the emission bands.

c Absolute quantum yields *Φ*_abs_. The relative fraction of TADF and phosphorescence (calculated from the deconvoluted spectra (Figures S13 and S14, Supporting Information)) is given in percent in parentheses.

d Shoulder.

e Biexponential decay, here the average lifetime is given.

Photostability of the PS-immobilized dyes accessed in anoxic conditions was good in case of **Pt(**^**Ph**^**PDP**^**Ph**^**)(Py)**, **Pt(**^**Ph**^**PDP**^**Ph**^**)(BuIm)**, **Pt(**^**Mes**^**PDP**^**C6F5**^**)(BN)**, and **Pt(**^**Mes**^**IPDP**^**C6F5**^**)(BN)**. These materials showed little (<5%) or no change in the emission intensity after 60 min irradiation with a xenon lamp (Figure S9, Supporting Information). Again, Mes substituent at the 3-position and Py and BuIm as terminal ligand were found to enhance the photostability. In case of **Pt(**^**Ph**^**PDP**^**Ph**^**)(BN)**, **Pt(**^**Ph**^**PDP**^**C6F5**^**)(BN)** and **Pt(**^**Ph**^**IPDP**^**C6F5**^**)(BN)** the luminescence intensity decreased by more than 10% in the same condition which may limit their use in sensory applications. It is important to note here that a comparison of the materials should be made with caution, since they absorb different amounts of photons and the xenon lamp shows variations in intensity depending on the excitation wavelength.

In addition to photostability, the thermal stability of four dyes (**Pt(**^**Mes**^**PDP**^**Ph**^**)(BN)**, **Pt(**^**Ph**^**PDP**^**Ph**^**)(BN)**, **Pt(**^**Ph**^**PDP**^**Ph**^**)(Py)** and **Pt(**^**Ph**^**PDP**^**Ph**^**)(BuIm)**) embedded in PS was also investigated. For this purpose, the embedded dyes were stored in water at 85 °C for 48 h and the emission spectra and lifetimes were measured before and after the storage. None of the dyes tested showed a measurable change in lifetime or emission spectrum, indicating high thermal stability of the materials below the glass transition temperature of PS (Figure S10, Supporting Information).

**Figure 5:**
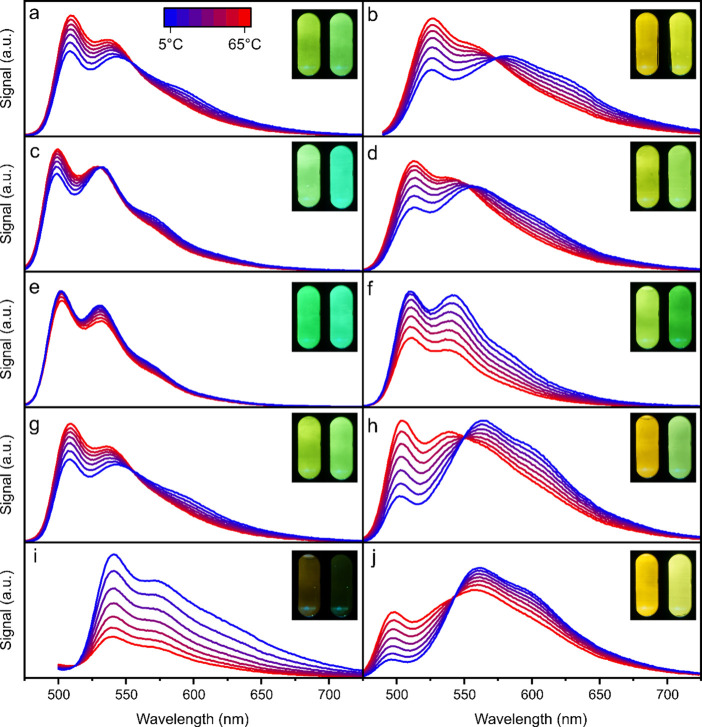
Temperature dependency of emission spectra for (a) **Pt(^Mes^PDP^Ph^)(BN)**, (b) **Pt(^Ph^PDP^Ph^)(BN)**, (c) **Pt(^Mes^PDP^C6F5^)(BN)**, (d) **Pt(^Ph^PDP^C6F5^)(BN)**, (e) **Pt(^Mes^IPDP^C6F5^)(BN)**, (f) **Pt(^Ph^IPDP^C6F5^)(BN)**, (g) **Pt(^Mes^PDP^*t*-BuPh^)(BN)**, (h) **Pt(^Ph^PDP^Ph^)(Py)**, (i) **Pt(^Ph^PDP^Ph^)(CO)**, and (j) **Pt(^Ph^PDP^Ph^)(BuIm)** immobilized in PS (0.5 wt %), recorded between 5 and 65 °C under anoxic conditions. The Pt(II) complexes were excited at 450 nm (e, f, j), 455 nm (a, c, d, g, h), 475 nm (b), and 480 nm (i). The insets show RGB images of the sensor materials at 5 °C (left) and 65 °C (right), excited by a blue LED (455 nm).

To evaluate potential suitability of the materials as temperature sensors, the emission spectra (Figure 5) and the decay times (Figure S11, Supporting Information) were measured in a temperature range between 5 and 65 °C. The emission spectra were deconvoluted to obtain separate TADF and phosphorescence spectra and access their contribution to the total emission (Figures S12–S14; Supporting Information). We assumed that both TADF and phosphorescence spectra maintain their shape over the entire temperature range.^40^ According to temperature sensitivity of the luminescence (Table 2) the complexes can be divided into two groups, namely materials for ratiometric read-out and for decay time-based temperature sensing.

**Figure 6:**
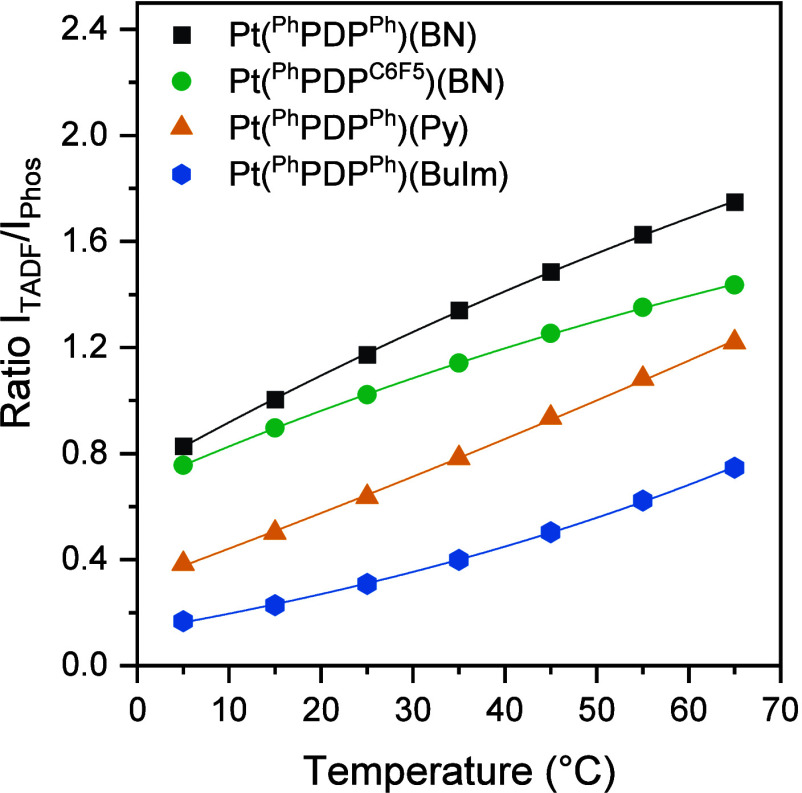
Temperature response on the ratio between TADF and phosphorescence intensities. The intensities were measured at the TADF and phosphorescence emission maxima (Table 2).

The complexes **Pt(**^**Ph**^**PDP**^**Ph**^**)(BN)**, **Pt(**^**Ph**^**PDP**^**C6F5**^**)(BN)**, **Pt(**^**Ph**^**PDP**^**Ph**^**)(Py)**, and **Pt(**^**Ph**^**PDP**^**Ph**^**)(BuIm)** are particularly suitable for ratiometric measurements showing very distinct spectral changes (Figure 5) and the highest sensitivities (dRatio d*T*^–1^) (Figure 6). These span between 1.2 and 2.7% K^–1^ and correlate well with the fraction of phosphorescence: the higher the fraction of phosphorescence the higher the sensitivity (Table 2).

The decay time for all Mes-substituted (4-position) complexes, **Pt(**^**Ph**^**IPDP**^**Ph**^**)(BN)** and **Pt(**^**Ph**^**PDP**^**Ph**^**)(CO)**, showing mainly TADF, but also for **Pt(**^**Ph**^**PDP**^**Ph**^**)(BN)** and **Pt(**^**Ph**^**PDP**^**C6F5**^**)(BN)**, is highly sensitive to temperature changes (dτ d*T*^–1^ between −1.4 and −2.8% K^–1^). With the exception of **Pt(**^**Ph**^**PDP**^**Ph**^**)(CO)** that shows low brightness, these complexes can be used as decay time-based temperature indicators similar to previously reported Zr(PDP)_2_ complexes.^46^

### Planar Temperature Sensors

Polystyrene (PS) based materials are unsuitable for temperature measurements in aerated environments, mainly because PS has a significant oxygen permeability (1.9 × 10^–13^ cm^3^ (STP) cm cm^–2^ s^–1^ Pa^–1^).^57^ Quenching by oxygen would result in significant signal decrease in the ratiometric mode and make the decay time read-out impossible. In order to eliminate oxygen interference, the complexes with the best ratiometric capabilities (**Pt(**^**Ph**^**PDP**^**Ph**^**)(BN)**, **Pt(**^**Ph**^**PDP**^**Ph**^**)(Py)**, **Pt(**^**Ph**^**PDP**^**Ph**^**)(BuIm)**, and **Pt(**^**Mes**^**PDP**^**Ph**^**)(BN)**) were embedded in virtually oxygen impermeable polyacrylonitrile (PAN, 0.00015 cm^3^ (STP) cm cm^–2^ s^–1^ Pa^–1^)^57^ and knife-coated on a transparent poly(ethylene terephthalate) support to make planar temperature sensor foils.

**Figure 7:**
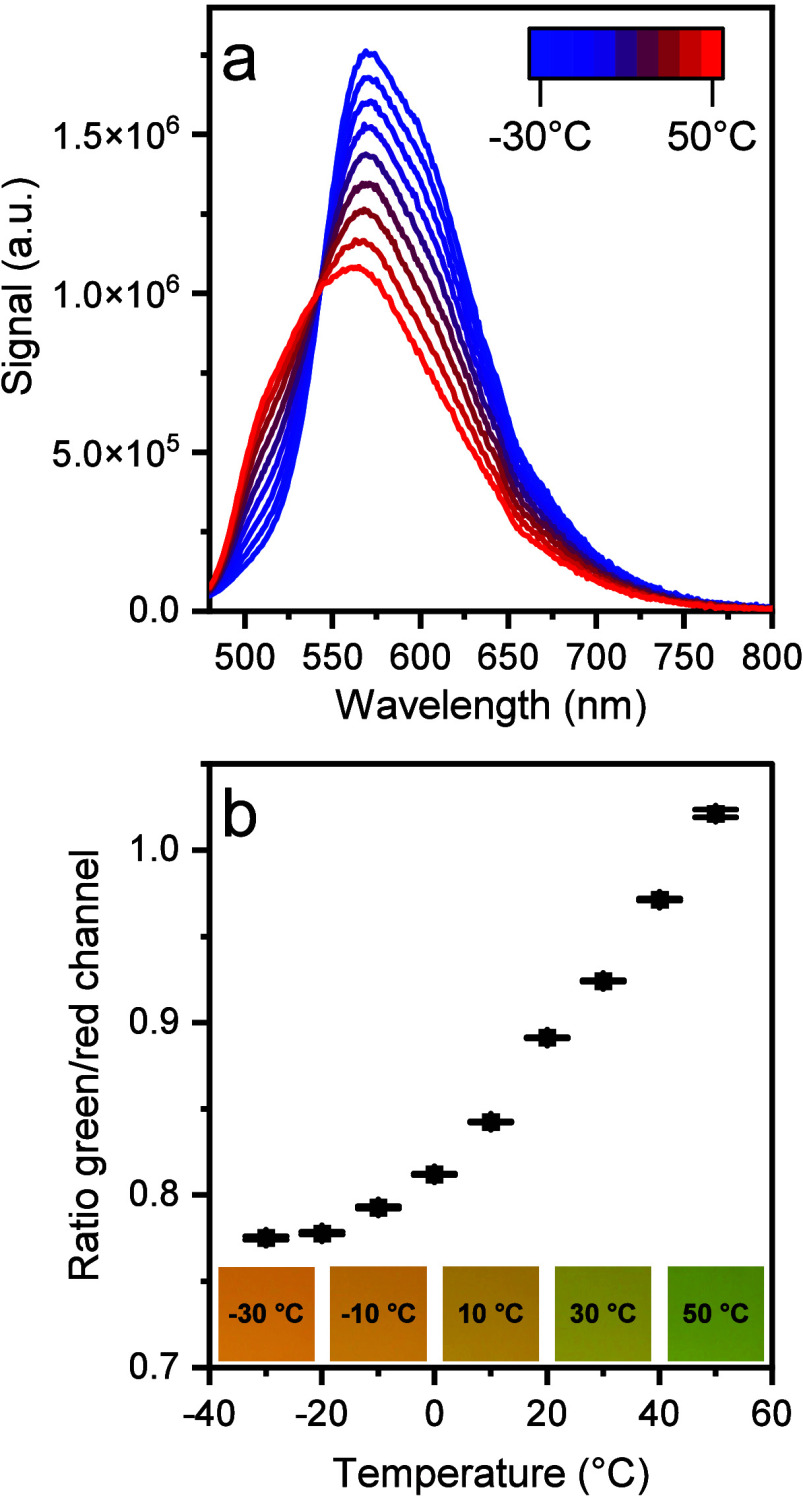
Temperature dependence of the (a) emission spectrum (λ_exc_ = 450 nm) and (b) green/red channel ratio of the photographic images for **Pt(**^**Ph**^**PDP**^**Ph**^**)(Py)** immobilized in PAN, acquired in air. Part (b) also shows RGB images taken at the respective temperature. The spectra were recorded with a Fluorolog-3 spectrometer and the images were taken with an RGB camera (Sony Alpha 6000) upon excitation with blue LED (455 nm).

Figure 7 and Figure S16 (Supporting Information) show that immobilization in PAN leads to a significant change in the shape of the emission spectra compared to toluene and PS. In fact, the contribution of TADF decreases so that this emission type is reduced to a shoulder. This can be rationalized by a change in the geometry of the complexes in the more polar matrix and is in line with spectral changes observed in solutions of the more polar solvents. Nevertheless, all investigated materials show excellent ratiometric response. Moreover, even **Pt(**^**Mes**^**PDP**^**Ph**^**)(BN)**, which did not show ratiometric sensing capabilities in polystyrene, represents a promising sensing material in combination with PAN (Figure S16, Supporting Information).

Although the ratiometric sensors can be conveniently read-out using two emission filters, spectral properties of the new materials make it also possible to utilize a consumer RGB camera/smartphone for temperature imaging. RGB images obtained upon excitation of the sensor foils with a blue LED (455 nm) in combination with an inexpensive plastic color filter (LEE 010 Medium Yellow) in front of the camera are shown in Figure 7b and Figure S16 (Supporting Information). For **Pt(**^**Ph**^**PDP**^**Ph**^**)(BN)**, **Pt(**^**Ph**^**PDP**^**Ph**^**)(Py)**, and **Pt(**^**Ph**^**PDP**^**Ph**^**)(BuIm)** in PAN the luminescence color changes from orange to green when heated. Below 0 °C almost the entire emission is due to phosphorescence, resulting in a nearly constant ratio between the green and the red channel. In case of **Pt(**^**Mes**^**PDP**^**Ph**^**)(BN)** the color change is less obvious but the ratio increases over the entire temperature range since both emission types are present at every temperature. It should be mentioned that the green and red channels of the used camera do not perfectly match the emission spectra leading to a partial detection of the phosphorescence signal in the green channel as well. It is therefore expected that much higher ratios between the green and red channel can be obtained by using a multibandpass filter.

### New Cellular Nanothermometers

**Figure 8:**
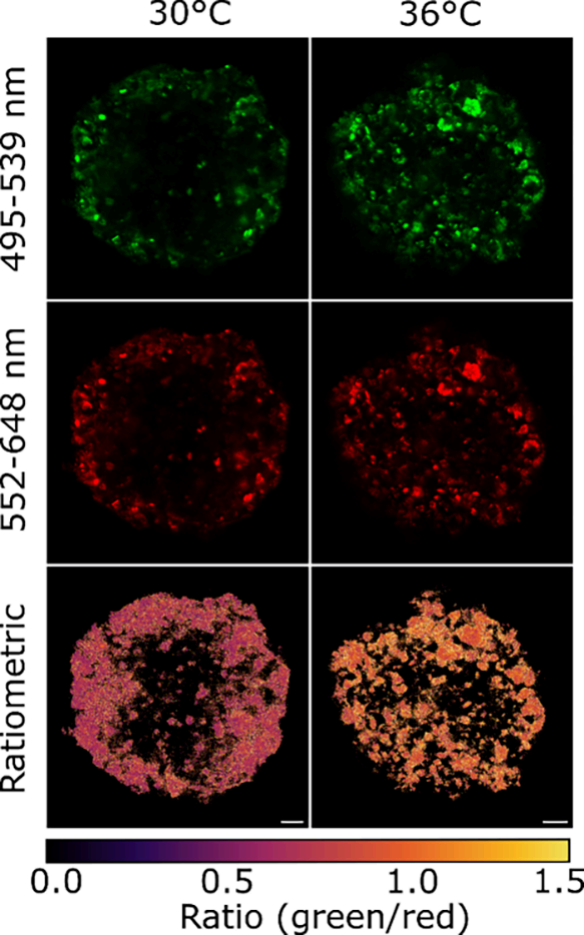
Confocal microscopy images of live HCT116 spheroids (excited at 485 nm) stained with 100 μg mL^–1^**Pt(**^**Ph**^**PDP**^**Ph**^**)(Py)**-RL100 nanoparticles and measured at 30 °C (left) and 36 °C (right). The green channels (top) were acquired by collecting the TADF emission from 495 to 539 nm and the red channel (middle) by collecting the phosphorescence from 552 to 648 nm. At the bottom, ratiometric images between the green and red channel are depicted. The scale bar is 25 μm.

Considering a prominent ratiometric response of **Pt(**^**Ph**^**PDP**^**Ph**^**)(Py)**, we tested utility of this dye in fluorescence nanothermometry.^58^ For encapsulation into nanoparticles, we chose RL-100 biocompatible polymer with moderate permeability to oxygen, enabling for improved cell interactivity, environmental protection.^59,60^ Produced RL-100 nanoparticles displayed excellent ratio response over 20–45 °C (Figure S17, Supporting Information) and efficiently stained human colon cancer HCT116 cells in adherent cultures, with no evident toxicity (not shown). Multicellular spheroids and “tumor avatars” represent important models for better understanding of the tumor microenvironment and precision oncology applications.^61,62^ At the same time, thermogenesis in cancer cells can represent a new therapeutic target.^63,64^ We therefore performed proof-of-principle experiments by staining HCT116 spheroids with RL-100 nanoparticles (Figure 8) and testing their ratio response in live 3D spheroids. Confocal live microscopy of RL-100-stained spheroids showed bright colocalized signals of TADF and phosphorescence, with measurable responses between 30 and 36 °C (Figure 8). This data confirms that **Pt(**^**Ph**^**PDP**^**Ph**^**)(Py)** shows sufficient sensitivity for “cancer cell” nanothermometry and is a promising indicator for encapsulation into nanoparticles for biomedical applications.

## 3 Conclusions

In summary, the herein presented Pt(II) pyridinedipyrrolide complexes show unique photophysical properties. They can be excited in the blue region of the electromagnetic spectrum and show simultaneous TADF and phosphorescence at room temperature. The ratio between green TADF and orange phosphorescence can easily be tuned by changing the substituents at pyrrole rings of the PDP ligand or by exchanging the terminal ligand. The type of emission can be adjusted from pure TADF to almost pure phosphorescence. These properties are utilized in two different types of self-referenced materials for optical sensing and imaging of temperature prepared by immobilization of the dyes into polymers. The materials showing predominantly TADF exhibit fairly high temperature dependency of the luminescence decay time (over −1.5% K^–1^ at 25 °C). In contrast, materials with simultaneous emission utilize conversion of phosphorescence into TADF with temperature and thus show ratiometric two-wavelength sensing capabilities. Moreover, spectral match between TADF/phosphorescence and green/red channels of the conventional cameras enables temperature mapping with very simple instrumentation requiring only blue light for the excitation and an inexpensive plastic long-pass filter in front of the camera. Furthermore, it was shown that the dye **Pt(**^**Ph**^**PDP**^**Ph**^**)(Py)** can be embedded in biocompatible nanoparticles, to suit growing needs of nanothermometry with various microtissue models, as we demonstrated with the human colon cancer spheroids model.

## 4 Experimental Section

### Materials

1-Butylimidazole (BuIm), 2,3,4,5,6-pentafluorobenzonitrile (PFBN), platinum(II) acetylacetonate (Pt(acac)_2_), 25–35% methylhydrosiloxane)-dimethylsiloxane copolymer (25–35 cSt), vinyl-terminated polydimethylsiloxane (1000 cSt), and 1,3,5,7-tetravinyl-1,3,5,7-tetramethylcyclotetrasiloxane were purchased from ABCR, D-(+)-glucose monohydrate, glucose oxidase from *Aspergillus niger* (∼200 U/mg), 1,2-dichlorobenzene, sodium propionate, polyacrylonitrile (PAN, average M.W. 150 000 Da), pyridine (Py), platinum-divinyltetramethyldisiloxane and sodium *tert*-butoxide (*t*-BuONa) were obtained from Sigma-Aldrich. Dichloromethane (DCM) and ethanol (EtOH) were from Fisher Scientific. Polystyrene (PS, average M.W. 260 000 Da) was from Acros Organics. Ammonium acetate (NH_4_OAc), sodium sulfite (Na_2_SO_3_), dimethylformamide (DMF), acetonitrile (MeCN, HPLC grade) and toluene (HPLC grade) were purchased from Carl Roth. *N*-Iodosuccinimide (NIS) was purchased from TCI Europe, 3-benzyl-5-(2-hydroxyethyl)-4-methylthiazolium chloride was from Fluorochem. Deuterated solvents were obtained from Eurisotop. Cyclohexane (CH), chloroform (CHCl_3_), and tetrahydrofuran (THF) were from VWR Chemicals. Silica gel 60 (0.063–0.200 mm), aluminum oxide 90 (active neutral, 0.063–0.200 mm), sodium chloride and heptane were purchased from Merck. Ultrafine titanium dioxide (TiO_2_) powder (UV-TITAN P170) was from Kemira. Poly(ethylene terephthalate) (PET) support was received from Pütz. Eudragit RL-100 (∼10% of quaternary ammonium groups, MW ∼ 150 000 Da) was from Degussa.

### Synthesis

2,6-Pyridinedicarboxaldehyde^65^ (**2**), 3-(2,3,4,5,6-pentafluorophenyl)-1-phenyl-2-propen-1-one^66^ (**1b**), *cis*-bis(benzonitrile)dichloroplatinum(II)^67^ (Pt(BN)_2_Cl_2_), and ligands (**H**_**2**_^**Ph**^**PDP**^**Ph**^, **H**_**2**_^**Mes**^**PDP**^**Ph**^, **H**_**2**_^**Mes**^**PDP**^**C6F5**^ and **H**_**2**_^**Mes**^**PDP**^*t***-BuPh**^)^46,47^ were synthesized according to the literature. A Monowave 50 synthesis reactor from Anton Paar was used for performing reactions under high pressure. Flash chromatography was performed by a Biotage Select system using Sfär Silica columns from Biotage.

#### Synthesis of 2,6-Bis(3-(perfluorophenyl)-5-phenyl-1H-pyrrol-2-yl)pyridine (**H**_**2**_^**Ph**^**PDP**^**C6F5**^)

In a flame-dried Schlenk tube, compound **1b** (251 mg, 842 μmol, 2 equiv), 2,6-pyridinedicarboxaldehyde (**2**) (55.8 mg, 413 μmol, 1 equiv), and 3-benzyl-5-(2-hydroxyethyl)-4-methylthiazolium chloride (54.0 mg, 200 mmol, 0.5 equiv) were dissolved in dry EtOH (4 mL) in an argon-filled atmosphere. The solution was degassed with argon for 15 min, and then *t*-BuONa (24.0 mg, 250 μmol, 0.6 equiv) was added during stirring. The dark brown mixture was stirred at reflux for 24 h. Afterward, the reaction was cooled to room temperature and transferred to a thick-walled pressure vessel. Ammonium acetate (230 mg, 2.98 mmol, 7 equiv) was then added and the mixture was heated to 170 °C for 3.5 h. Upon cooling to room temperature, the solvent was removed under reduced pressure and the product was purified via column chromatography using silica gel and a gradient of CH to CH:DCM (7:3 v/v) as the eluent. The final product was isolated as a white solid, with a yield of 170 mg (59%).

^1^H NMR (500 MHz, CD_2_Cl_2_) δ 9.87 (s, 2H), 7.65 (d, *J* = 7.2 Hz, 4H), 7.50–7.46 (m, 4H), 7.44 (t, *J* = 7.9 Hz, 1H), 7.34 (t, *J* = 7.4 Hz, 2H), 6.88 (d, *J* = 7.9 Hz, 2H), 6.68 (d, *J* = 3.0 Hz, 2H). ^13^C NMR (126 MHz, CD_2_Cl_2_) δ 149.87, 145.16, 141.05, 138.57, 138.20, 134.34, 131.77, 130.66, 129.68, 128.08, 124.94, 117.58, 112.12, 111.00, 108.59. ^19^F NMR (470 MHz, CD_2_Cl_2_) δ −140.03 (dd, *J* = 23.4, 8.1 Hz), −156.64 (t, *J* = 20.9 Hz), −162.82 (ddd, *J* = 23.3, 21.0, 8.2 Hz).

MS (APCI) *m*/*z*: calcd. for C_37_H_18_F_10_N_3_ [M + H]^+^: 694.1, found: 694.8

#### Synthesis of **Pt(**^**Ph**^**PDP**^**Ph**^**)(BN)**

In a thick-walled pressure vessel, **H**_**2**_^**Ph**^**PDP**^**Ph**^ (15.5 mg, 30.1 μmol, 1 equiv) and sodium propionate (8.7 mg, 90.4 μmol, 3 equiv) were suspended in 1,2-dichlorobenzene (1 mL). Then, Pt(BN)_2_Cl_2_ (8.3 mg, 17.6 μmol, 0.6 equiv) was added and the mixture was heated to 250 °C for 10 min. After cooling to room temperature, additional 0.6 equiv of Pt(BN)_2_Cl_2_ were added, and the mixture was heated once more to 250 °C for 10 min. This process of adding Pt(BN)_2_Cl_2_ and heating was repeated until no more **H**_**2**_^**Ph**^**PDP**^**Ph**^ was detected via TLC analysis. Once the reaction was complete, the mixture was filtered through aluminum oxide to eliminate elemental platinum. The solvent was then evaporated under reduced pressure and the solid residue was adsorbed onto aluminum oxide. The adsorbed product was then passed through a prepacked Sfär Silica column and purified via flash chromatography, using a gradient of CH to CH:DCM (3:2 v/v) as the eluent. The final product was obtained as an orange solid, with a yield of 17 mg (70%).

^1^H NMR (300 MHz, CD_2_Cl_2_) δ 7.68 (d, *J* = 6.7 Hz, 4H), 7.60–7.46 (m, 5H), 7.46–7.37 (m, 4H), 7.36–7.04 (m, 9H), 6.83 (t, *J* = 7.4 Hz, 2H), 6.72 (d, *J* = 8.1 Hz, 2H), 6.62 (d, *J* = 7.4 Hz, 2H), 6.26 (s, 2H). ^13^C NMR (76 MHz, CD_2_Cl_2_) δ 155.84, 146.59, 138.97, 137.53, 137.29, 136.89, 134.60, 133.54, 132.29, 130.03, 129.94, 128.88, 128.66, 128.21, 127.18 (overlay of two peaks), 120.36, 113.65, 110.44, 109.93.

HRMS (MALDI) *m*/*z*: [M]^+^ calcd. for C_44_H_30_N_4_Pt: 808.2097; found 808.2100

#### Synthesis of **Pt(^Ph^PDP^C6F5^)(BN)**

The synthesis of **Pt(**^**Ph**^**PDP**^**C6F5**^**)(BN)** was performed analogously to **Pt(**^**Ph**^**PDP**^**Ph**^**)(BN)**. However, 20.1 mg (29.0 μmol, 1 equiv) of **H**_**2**_^**Ph**^**PDP**^**C6F5**^, 14.4 mg (149 μmol, 5.2 equiv) of sodium propionate and 8.1 mg (17.2 μmol, 0.6 equiv) of Pt(BN)_2_Cl_2_ suspended in 1 mL 1,2-dichlorobenzene were used instead. The product was isolated as an orange solid by column chromatography using a gradient from CH to CH:DCM (7:3 v/v) as an eluent. Yield: 13 mg (45%).

^1^H NMR (500 MHz, CD_2_Cl_2_) δ 7.66 (d, *J* = 7.1 Hz, 4H), 7.56 (t, *J* = 7.7 Hz, 1H), 7.30 (t, *J* = 8.1 Hz, 1H), 7.25 (t, *J* = 7.7 Hz, 2H), 7.17 (t, *J* = 7.5 Hz, 4H), 6.84 (t, *J* = 7.5 Hz, 2H), 6.64 (d, *J* = 7.3 Hz, 2H), 6.33 (d, *J* = 8.1 Hz, 2H), 6.29 (s, 2H). ^13^C NMR (126 MHz, CD_2_Cl_2_) δ 155.20, 147.27, 144.95, 140.85, 140.07, 139.37, 138.45, 136.20, 134.83, 133.56, 130.10, 128.72, 128.27, 127.58, 114.10, 111.83, 110.82, 109.59. ^19^F NMR (470 MHz, CD_2_Cl_2_) δ −140.22 (dd, *J* = 23.9, 8.1 Hz), −157.18 (t, *J* = 20.9 Hz), −163.12 (ddd, *J* = 23.9, 21.0, 8.2 Hz).

HRMS (MALDI) *m*/*z*: [M]^+^ calcd. for C_44_H_20_F_10_N_4_Pt: 988.1155; found 988.1169

#### Synthesis of **Pt(^Mes^PDP^Ph^)(BN)**

The synthesis of **Pt(**^**Mes**^**PDP**^**Ph**^**)(BN)** was performed analogously to **Pt(**^**Ph**^**PDP**^**Ph**^**)(BN)**. However, 15.0 mg (25.1 μmol, 1 equiv) of **H**_**2**_^**Mes**^**PDP**^**Ph**^, 7.3 mg (76.0 μmol, 3 equiv) of sodium propionate, and 7.0 mg (14.9 μmol, 0.6 equiv) of Pt(BN)_2_Cl_2_ suspended in 1 mL of 1,2-dichlorobenzene were used instead. The product was isolated as an orange solid by column chromatography using a gradient from CH to CH:DCM (7:3 v/v) as an eluent. Yield: 7.8 mg (35%).

^1^H NMR (300 MHz, CD_2_Cl_2_) δ 7.60–7.47 (m, 5H), 7.41 (q, J = 7.9 Hz, 6H), 7.27 (t, *J* = 7.3 Hz, 2H), 7.11–7.00 (m, 3H), 6.72 (d, *J* = 8.1 Hz, 2H), 6.42 (s, 4H), 5.84 (s, 2H), 2.23 (s, 12H), 1.48 (s, 6H). ^13^C NMR (76 MHz, CD_2_Cl_2_) δ 155.93, 144.54, 138.95, 138.40, 137.73, 137.64, 135.64, 134.23, 133.92, 132.73, 132.14, 129.90, 128.82, 128.75, 128.19, 126.80, 118.12, 112.60, 110.64, 109.15, 21.05, 20.95.

HRMS (MALDI) *m*/*z*: [M]^+^ calcd. for C_50_H_42_N_4_Pt: 892.3036; found 892.3043.

#### Synthesis of **Pt(^Mes^PDP^*t*-BuPh^)(BN)**

The synthesis of **Pt(**^**Mes**^**PDP**^*t***-BuPh**^**)(BN)** was performed analogously to **Pt(**^**Ph**^**PDP**^**Ph**^**)(BN)**. However, 20.2 mg (28.4 μmol, 1 equiv) of **H**_**2**_^**Mes**^**PDP**^*t***-BuPh**^, 8.2 mg (85.3 μmol, 3 equiv) of sodium propionate, and 8.7 mg (18.5 μmol, 0.6 equiv) of Pt(BN)_2_Cl_2_ suspended in 1 mL of 1,2-dichlorobenzene were used instead. The product was isolated as an orange solid by column chromatography using a gradient from CH to CH:DCM (7:3 v/v) as an eluent. Yield: 15 mg (53%).

^1^H NMR (300 MHz, CD_2_Cl_2_) δ 7.56 (t, *J* = 7.5 Hz, 1H), 7.43 (s, 10H), 7.09 (t, *J* = 8.1 Hz, 1H), 7.03 (d, *J* = 7.3 Hz, 2H), 6.79 (d, *J* = 8.1 Hz, 2H), 6.42 (s, 4H), 5.82 (s, 2H), 2.23 (s, 12H), 1.48 (s, 6H), 1.36 (s, 18H). ^13^C NMR (76 MHz, CD_2_Cl_2_) δ 155.96, 149.80, 144.50, 138.90, 138.41, 137.60, 135.59, 134.65, 134.30, 133.89, 132.72, 132.07, 129.43, 128.81, 128.18, 125.70, 118.08, 112.65, 110.68, 109.11, 34.99, 21.06, 20.96.

HRMS (MALDI) *m*/*z*: [M]^+^ calcd. for C_58_H_58_N_4_Pt: 1004.4288; found 1004.4271.

#### Synthesis of **Pt(^Mes^PDP^C6F5^)(BN)**

The synthesis of **Pt(**^**Mes**^**PDP**^**C6F5**^**)(BN)** was performed analogously to **Pt(**^**Ph**^**PDP**^**Ph**^**)(BN)**. However, 20.9 mg (26.9 μmol, 1 equiv) of **H**_**2**_^**Mes**^**PDP**^**C6F5**^, 12.3 mg (128 μmol, 4.8 equiv) of sodium propionate, and 10.4 mg (21.9 μmol, 0.8 equiv) of Pt(BN)_2_Cl_2_ suspended in 1 mL of 1,2-dichlorobenzene were used instead. The product was isolated as an orange solid by column chromatography using a gradient from CH to CH:DCM (7:3 v/v) as an eluent. Yield: 17 mg (49%).

^1^H NMR (500 MHz, CD_2_Cl_2_) δ 7.58 (t, *J* = 7.6 Hz, 1H), 7.44 (t, *J* = 7.1 Hz, 2H), 7.25 (t, *J* = 8.1 Hz, 1H), 7.04 (d, *J* = 7.7 Hz, 2H), 6.43 (s, 4H), 6.27 (d, *J* = 7.1 Hz, 2H), 5.90 (s, 2H), 2.20 (s, 12H), 1.48 (s, 6H). ^13^C NMR (126 MHz, CD_2_Cl_2_) δ 155.31, 144.98, 144.86, 140.58, 140.00, 138.43, 138.41, 138.06, 137.86, 134.17, 133.44, 132.76, 128.92, 128.53, 128.23, 113.32, 112.38, 111.54, 110.31, 109.77, 20.94, 20.91. ^19^F NMR (470 MHz, CD_2_Cl_2_) δ −140.42 (dd, *J* = 23.9, 7.9 Hz), −158.02 (t, *J* = 20.9 Hz), −163.45 – −163.71 (m).

HRMS (MALDI) *m*/*z*: [M]^+^ calcd. for C_50_H_32_F_10_N_4_Pt: 1072.2095; found 1072.2057

#### Synthesis of **Pt(**^**Ph**^**PDP**^**Ph**^**)(Py)**

In a thick-walled pressure vessel, **H**_**2**_^**Ph**^**PDP**^**Ph**^ (15.2 mg, 29.6 μmol, 1 equiv), platinum(II) acetylacetonate (15.3 mg, 38.5 μmol, 1.3 equiv), sodium propionate (15.0 mg, 156 μmol, 5 equiv), and pyridine (11.8 mg, 149 μmol, 5 equiv) were dissolved in 1,2-dichlorobenzene (1 mL). The mixture was heated to 250 °C for 30 min. Upon cooling to room temperature, the mixture was filtered through aluminum oxide to remove elemental platinum. The solvent was then removed under reduced pressure, and the remaining solid was adsorbed onto aluminum oxide. The adsorbed product was then loaded onto a prepacked Sfär Silica column and purified through flash chromatography using a gradient of CH to CH:DCM (1:1 v/v) as the eluent. The final product was isolated as a yellow solid, with a yield of 15.9 mg (68%).

^1^H NMR (300 MHz, CD_2_Cl_2_) δ 8.34 (d, *J* = 4.8 Hz, 2H), 7.51 (d, *J* = 7.0 Hz, 4H), 7.42 (t, *J* = 7.5 Hz, 4H), 7.31 (t, *J* = 7.2 Hz, 2H), 7.14–6.98 (m, 6H), 6.80–6.66 (m, 8H), 6.38 (t, *J* = 6.9 Hz, 2H), 6.16 (s, 2H). ^13^C NMR (76 MHz, CD_2_Cl_2_) δ 155.57, 154.40, 146.57, 138.61, 138.54, 138.28, 137.44, 131.91, 129.95, 128.86, 128.56, 127.98, 127.08, 126.20, 125.43, 113.82, 110.43.

HRMS (MALDI) *m*/*z*: [M]^+^ calcd. for C_42_H_30_N_4_Pt: 784.2097; found 784.2064.

#### Synthesis of **Pt(^Ph^PDP^Ph^)(BuIm)**

The synthesis of **Pt(**^**Mes**^**PDP**^**C6F5**^**)(BuIm)** was performed analogously to **Pt(**^**Ph**^**PDP**^**Ph**^**)(Py)**. However, 15.4 mg (29.9 μmol, 1 equiv) of **H**_**2**_^**Ph**^**PDP**^**Ph**^, 14.0 mg (146 μmol, 4.9 equiv) of sodium propionate, 19.0 mg (153 μmol, 5.1 equiv) of 1-butylimidazole, and 15.3 mg (38.5 μmol, 1.3 equiv) of platinum(II) acetylacetonate suspended in 1 mL of 1,2-dichlorobenzene were used instead. The product was isolated as an yellow solid by column chromatography using a gradient from CH to CH:DCM (1:1 v/v) as an eluent. Yield: 14 mg (56%).

^1^H NMR (300 MHz, CD_2_Cl_2_) δ 7.50 (d, *J* = 7.0 Hz, 4H), 7.41 (t, *J* = 7.5 Hz, 4H), 7.30 (t, *J* = 7.2 Hz, 2H), 7.16–7.05 (m, 5H), 6.91 (dd, *J* = 5.2, 2.1 Hz, 7H), 6.71 (d, *J* = 8.0 Hz, 2H), 6.51 (s, 1H), 6.19 (s, 2H), 5.91 (s, 1H), 3.17 (t, *J* = 7.8 Hz, 2H), 1.50–1.41 (m, 2H), 1.34 (t, *J* = 7.5 Hz, 2H), 0.99 (t, *J* = 7.2 Hz, 3H). ^13^C NMR (76 MHz, CD_2_Cl_2_) δ 155.56, 146.64, 138.45, 138.20, 137.56, 137.11, 131.89, 130.95, 129.96, 128.83, 128.38, 127.49, 127.01, 125.84, 119.35, 113.55, 110.40, 48.18, 32.06, 20.57, 13.90.

HRMS (MALDI) *m*/*z*: [M]^+^ calcd. for C_44_H_37_N_5_Pt: 829.2676; found 829.2690.

#### Synthesis of **Pt(^Ph^PDP^Ph^)(CO)**

The synthesis of **Pt(**^**Mes**^**PDP**^**C6F5**^**)(BuIm)** was performed analogously to **Pt(**^**Ph**^**PDP**^**Ph**^**)(Py)**. However, 15.2 mg (29.63 μmol, 1 equiv) of **H**_**2**_^**Ph**^**PDP**^**Ph**^, 14.1 mg (147 μmol, 5 equiv) of sodium propionate, 29.1 mg (151 μmol, 5.1 equiv) of 2,3,4,5,6-pentafluorobenzonitrile, and 15.6 mg (39.3 μmol, 1.3 equiv) of platinum(II) acetylacetonate suspended in 1 mL of 1,2-dichlorobenzene were used instead. The product was isolated as an red solid by column chromatography using a gradient from CH to CH:DCM (1:1 v/v) as an eluent. Yield: 8.9 mg (41%).

^1^H NMR (300 MHz, CD_2_Cl_2_) δ 7.57–7.27 (m, 20H), 7.19 (t, *J* = 8.1 Hz, 1H), 6.83 (d, *J* = 8.0 Hz, 2H), 6.29 (s, 2H). ^13^C NMR (76 MHz, CD_2_Cl_2_) δ 164.33, 155.46, 147.31, 140.82, 137.32, 137.27, 136.63, 133.18, 130.44, 129.85, 129.02, 128.60, 128.53, 127.61, 112.88, 111.22.

HRMS (MALDI) *m*/*z*: [M]^+^ calcd. for C_38_H_25_N_3_OPt: 733.1625; found 733.1640.

#### Synthesis of **Pt(^Ph^IPDP^Ph^)(BN)**

**Pt(**^**Ph**^**PDP**^**Ph**^**)(BN)** (14.7 mg, 18.2 μmol, 1 equiv) was dissolved in chloroform (1 mL) and NIS (8.4 mg, 37.2 μmol, 2.1 equiv) was added. The solution was stirred for 1 h at room temperature. Afterward, the mixture was filtered through aluminum oxide and the solvent was removed under reduced pressure. The remaining yellow solid was purified by column chromatography (aluminum oxide) using a gradient from heptane to heptane:DCM (1:1 v/v) as an eluent and the product was isolated as a yellow solid. Yield: 12 mg (62%).

^1^H NMR (500 MHz, CD_2_Cl_2_) δ 7.61 (t, *J* = 7.8 Hz, 1H), 7.48–7.45 (m, 8H), 7.41–7.37 (m, 6H), 7.31 (t, *J* = 7.7 Hz, 2H), 7.04 (t, *J* = 7.5 Hz, 4H), 6.98 (t, *J* = 8.2 Hz, 1H), 6.92 (d, *J* = 6.6 Hz, 2H), 6.58 (t, *J* = 7.5 Hz, 2H), 6.25 (d, *J* = 8.2 Hz, 2H). ^13^C NMR (126 MHz, CD_2_Cl_2_) δ 155.25, 146.82, 139.30, 138.43, 136.70, 136.46, 134.70, 134.02, 133.57, 131.58, 131.24, 128.90, 128.42, 128.14, 128.01, 127.96, 119.11, 111.23, 109.31, 70.08.

HRMS (MALDI) *m*/*z*: [M]^+^ calcd. for C_44_H_28_I_2_N_4_Pt: 1060.0031; found 1060.0046.

#### Synthesis of **Pt(^Mes^IPDP^C6F5^)(BN)**

The synthesis of **Pt(**^**Mes**^**IPDP**^**C6F5**^**)(BN)** was performed analogously to **Pt(**^**Ph**^**IPDP**^**Ph**^**)(BN)**. However, 16.4 mg (9.31 μmol, 1 equiv) of **Pt(**^**Mes**^**PDP**^**C6F5**^**)(BN)** and 4.47 mg (19.9 μmol, 2.1 equiv) of NIS dissolved in 1 mL of chloroform were used instead and the solution was stirred for 4 h. The product was isolated as an yellow solid by column chromatography using a gradient from CH to CH:DCM (4:1 v/v) as an eluent. Yield: 7.0 mg (57%).

^1^H NMR (300 MHz, CD_2_Cl_2_) δ 7.62 (t, *J* = 7.6 Hz, 1H), 7.47 (t, *J* = 8.0 Hz, 2H), 7.24 (t, *J* = 8.1 Hz, 1H), 7.06 (d, *J* = 7.0 Hz, 2H), 6.46 (s, 4H), 6.16 (d, *J* = 8.2 Hz, 2H), 2.12 (s, 12H), 1.49 (s, 6H). ^13^C NMR (126 MHz, CD_2_Cl_2_) δ 154.68, 147.25, 145.00, 141.53, 140.60, 139.67, 138.97, 138.58, 138.49, 134.44, 132.81, 132.50, 129.10, 128.37, 116.56, 111.63, 110.55, 110.12, 70.39, 30.25, 21.08, 20.43. ^19^F NMR (376 MHz, CD_2_Cl_2_) δ −138.37 (dd, *J* = 24.0, 8.3 Hz), −155.37 (t, *J* = 20.9 Hz), −162.74 (ddd, *J* = 24.0, 20.9, 8.4 Hz).

HRMS (MALDI) *m*/*z*: [M]^+^ calcd. for C_50_H_30_F_10_I_2_N_4_Pt: 1324.0027; found 1324.0028.

### Preparation of Planar Sensor Foils

#### PS Sensor Foils

A “cocktail” was prepared by dissolving the dye (0.5 wt % in respect to the polymer) and polystyrene (10 wt % in respect to the solvent) in chloroform. The “cocktail” was knife-coated on a dust-free PET support with a wet film thickness of 25 μm and the foils were dried for 1 day at room temperature.

#### PAN Sensor Foils

Dye (0.5 wt % in respect to the polymer) and PAN (10 wt % in respect to the solvent) were dissolved in dry DMF and the “cocktail 1” was knife-coated in a glovebox on a dust-free PET support (wet film thickness of 25 μm). After drying the foil for 12 h at room temperature, the foil was transferred from the glovebox to a drying chamber and was dried for another 12 h at 70 °C. Then, the PAN film was coated with a scattering layer (“cocktail 2”, wet film thickness of 25 μm) consisting of 100 mg of TiO_2_, 400 mg of vinyl-terminated polydimethylsiloxane, 16 μL of (25–35% methylhydrosiloxane)-dimethylsiloxane copolymer, 1 μL of 1,3,5,7-tetravinyl-1,3,5,7-tetramethylcyclotetrasiloxane, and 2 μL of platinum-divinyltetramethyldisiloxane complex dispersed/dissolved in 800 mg of “*n*-hexane”. The sensor films were kept in an oven (60 °C) for 15 min to complete the polymerization of the scattering layer.

### Preparation of Water Dispersible Nanoparticles

First 0.2 mg of **Pt(**^**Ph**^**PDP**^**Ph**^**)(Py)** and 20 mg of RL-100 were dissolved in a 6 mL mixture of acetone and THF (9 + 1 v/v). Water (40 mL) was then added rapidly to the solution under vigorous stirring and the organic solvents were removed under reduced pressure. Finally, the suspension was concentrated to a final volume of 10 mL.

### Measurements

^1^H, ^13^C{^1^H}{^19^F}, ^13^C-APT, and ^19^F NMR spectra were recorded on a Bruker Avance 300 MHz spectrometer or a Varian INOVA 500 MHz spectrometer. The residual signal of the deuterated solvent was used as an internal standard for calibration of ^1^H and ^13^C spectra.

An Expression CMS L compact mass spectrometer from Advion equipped with an atmospheric-pressure chemical ionization (APCI) source and quadrupole mass analyzer (range 10–2000 *m*/*z*) was used to acquire mass spectra (MS) for reaction monitoring and characterization of intermediates. High resolution mass spectra (HRMS) of the platinum(II) complexes were recorded on the matrix-assisted laser desorption/ionization time-of-flight mass spectrometer (MALDI-TOF MS) Micromass MALDI micro MX from Waters.

UV–vis spectra were recorded on a CARY 60 UV–vis spectrophotometer from Varian for dye concentrations of 15–30 μmol L^–1^. Luminescence spectra (*c* = 6–10 μmol L^–1^) and relative quantum yields were recorded using a Fluorolog-3 luminescence spectrometer equipped with a NIR-sensitive R2658 photomultiplier from Hamamatsu. The dye characterization in solution was performed in a screw-capped quartz cuvette from Hellma Analytics. The removal of oxygen from toluene solutions was achieved by passing high-purity nitrogen (99.99999% purity) from Linde Gas through the solution for a minimum of 15 min. For measurements at 77 K the platinum(II) complexes were either dissolved in a mixture of toluene and THF (4:6 v/v) or immobilized in PS and the sample was placed in a cryostat from Horiba Scientific cooled with liquid nitrogen. To measure emission spectra of dyes in PS between 5 and 65 °C, the foils were arranged diagonally within a precision quartz glass cuvette (10 mm) containing a 5 wt % aqueous solution of Na_2_SO_3_. Temperature was adjusted with a Cary SPV-1 × 0 Single Cell Peltier Accessory Peltier element from Varian in combination with a F12-ED refrigerated/heating circulator from Julabo. To measure the emission spectra of the PAN sensor foils, the foils were attached to a custom-made cooling/heating device, and the temperature was controlled between −30 and 50 °C. To minimize the risk of ice formation at low temperatures, the measurement chamber was continuously purged with dry nitrogen. Emission data were collected using the front-face orientation mode of the Fluorolog-3 spectrometer.

Absolute quantum yields in polystyrene were measured under oxygen-free conditions at room temperature (22 °C) on a Fluorolog-3 spectrometer equipped with an integrating sphere Quanta-phi. Deoxygenation was performed by using a 5 wt % glucose solution containing traces of glucose oxidase.

Luminescence decay times were measured via time correlated single photon counting (TCSPC) on a Fluorolog-3 spectrometer equipped with a spectraLED (λ = 392 nm) excitation source and a DeltaHub module from Horiba. Data analysis was performed on DAS-6 Analysis software from Horiba using a mono- or biexponential fit.

Decay time temperature response curves were fitted via an Arrhenius type model:^68^
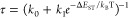
1where *k*_0_ is the temperature-independent decay rate for the excited-state deactivation, *k*_1_ is a pre-exponential factor, *k*_B_ is the Boltzmann constant, Δ*E*_ST_ is the energy gap between the first triplet and first singlet state, and *T* is the absolute temperature.

The photostability of the platinum(II) complexes and tetramethylrhodamine ethyl ester perchlorate (TMR) in solution was evaluated with a previously described homemade setup that uses a metal-halide lamp as an excitation source.^69^

To access the photostability of the PS-embedded complexes, the PS sensor films were placed in a 5 wt % aqueous solution of Na_2_SO_3_ at 25 °C and continuously excited with the xenon lamp of a Fluorolog-3 spectrometer at their lowest energy absorption maximum for 1 h. The emission intensities were measured every ten seconds at the emission maximum.

Deconvolution of dual emission spectra to determine the ratio between TADF and phosphorescence in PS was performed according to a previously published method.^70^

### Ratiometric Temperature Readout with an RGB Camera

The PAN sensor foils were tabbed on a homemade cooling/heating setup and the temperature was set between −30 and 50 °C. To prevent ice formation on the setup at low temperatures, the measurements were performed in a transparent PS-chamber with a dry atmosphere. The sensor foils were excited with a blue high-power LED (455 nm) from OSRAM. The images were made with a digital camera (Sony Alpha 6000), equipped with a 16–50 mm E-Mount zoom lens (SEL-P1650) from Sony. Parameters of the camera were set as follows: aperture F/5.6; ISO 100; shutter speed 0.8 s (**Pt(**^**Ph**^**PDP**^**Ph**^**)(BN)**) or 1.3 s (**Pt(**^**Ph**^**PDP**^**Ph**^**)(Py)**, **Pt(**^**Ph**^**PDP**^**Ph**^**)(BuIm)** and **Pt(**^**Mes**^**PDP**^**Ph**^**)(BN)**). A LEE 010 Medium Yellow filter was fixed in front of the camera to cut off the excitation light. The RAW images were separated into their blue, green, and red channels, and the mean ratio between the green and red channels was calculated for four regions of interest.

### Cell Measurements

Human colon carcinoma HCT116 cells from ATCC were handled essentially as described before.^46^ Briefly, HCT116 was cultured in McCoy’s 5A media (VWR, 392–0420) supplemented with 10% heat-inactivated FBS (Gibco, 12662), 1 mM sodium pyruvate (Gibco, 11360), and 10 mM HEPES (Gibco, 15630) and cultured under in a humidified incubator at 37 °C, 18.6% O_2_, and 5% CO_2_. At 80–90% confluency cells were passaged using 0.25% trypsin/1 mM EDTA (Gibco, 25300104) solution. Cells were cultured and analyzed under antibiotic-free conditions.

Spheroids were formed by seeding (initial 100 cells) together with 100 μg/mL **Pt(**^**Ph**^**PDP**^**Ph**^**)(Py)**-RL100 nanoparticles on a 0.5 wt % Lipidure-coating on a U-bottom 96-well plate (low attachment method). Formation was performed over 6 days, before transferring on a precoated (0.07 mg/mL collagen IV, 0.03 mg/mL poly-d-Lysine) multiwell microscopy dishes (μ-slide 8-well, Ibidi GmbH, Germany), and microscopy.

Confocal FLIM microscopy was performed on an inverted Stellaris 8 Falcon (Leica) microscope (Ghent Light Microscopy Core), equipped with the white-light laser (440–790 nm), HC PL Apo 10*x*/0.4 air, HC Fluotar 25*x*/0.95 W, HC PL Apo 40*x*/1.25 GLYC corr., HC PL Apo 63*x*/1.4 Oil objectives, HyD X, HyD R and HyD S detectors, temperature-controlled incubator (Okolab), and dedicated LAS X acquisition and analysis software, as described previously.^46^ Spheroids were pre-equilibrated to the set temperature (5–10 min) and imaged using HC PL Apo 40*x*/1.25 Glyc corr. Objective, 512 × 512 resolution, scan speed either 200 Hz (pixel dwell time 7.69 μs) or 400 Hz (pixel dwell time 3.16 μs), pinhole 2 AU. Nanoparticles were excited with 485 nm (30% power) with emission collected at 495–539 nm (TADF) and 552–648 nm (phosphorescence).

### Computational Methods

A detailed description of the computational methods can be found in the Supporting Information.

## Supporting Information Available

Additional experimental data as well as NMR and MS spectra for all compounds (Figures S1–S54 and Table S1) (PDF)
