# Supplementary material for: Tunable Self-Referenced Molecular Thermometers via Manipulation of Dual Emission in Platinum(II) Pyridinedipyrrolide Complexes
Source: ACS Appl Mater Interfaces. 2024 Feb 23;16(9):11930–43. doi: 10.1021/acsami.3c19226 (PMC10921383; doi:10.1021/acsami.3c19226)
Supplement: Supplementary file 1 — am3c19226_si_001.pdf [file am3c19226_si_001.pdf]

Supporting Information

Tunable Self-Referenced Molecular Thermometers via Manipulation of Dual Emission in Platinum(II) Pyridinedipyrrolide Complexes

*Andreas Russegger[1], Susanne M. Fischer[2], Angela C. Debruyne[3], Helmar Wiltsche[1], A. Daniel Boese[2], Ruslan I. Dmitriev[3,4], Sergey M. Borisov[1]**

[1] Institute of Analytical Chemistry and Food Chemistry, Graz University of Technology, Stremayrgasse 9, 8010 Graz, Austria

[2] Physical and Theoretical Chemistry, Institute of Chemistry, University of Graz, Heinrichstrasse 28/IV, 8010 Graz, Austria

[3] Tissue Engineering and Biomaterials Group, Department of Human Structure and Repair, Faculty of Medical and Health Sciences, Ghent University, C. Heymanslaan 10, Ghent, 9000 Belgium

[4] Ghent Light Microscopy Core, Ghent University, Ghent, 9000 Belgium

E-mail: sergey.borisov@tugraz.at

Content

1. Photophysical Properties in Solution 3
   1.1. Absorption and emission spectra in toluene 3
   1.2. Solvatochromism 4
   1.3. Luminescence decay in toluene 4
   1.4. Photostability in toluene 5
2. Computational Methods 7
   2.1. Ground-state calculations 7
   2.2. Excited-state calculations 7
   2.3. Results of DFT and TDDFT calculations 7
3. Photophysical Properties in PS 9
   3.1. Emission spectra 9
   3.2. Luminescence decay in PS 10
   3.3. Photostability in PS 10
   3.4. Temperature stability in PS 11
   3.5. Temperature sensitivity (decay time) 12
   3.6. Deconvolution of emission spectra of Pt(II) complexes embedded in PS 13
   3.7. Contributions of TADF and phosphorescence 14
   3.8. Temperature sensitivity (ratiometric read-out) 16
4. Applications 17
   4.1. Ratiometric readout of planar PAN sensor foils 17
   4.2. Temperature Nanosensors 18
5. NMR Spectra 19
6. Mass Spectra 34
7. References 45

1. Photophysical Properties in Solution

1.1. Absorption and emission spectra in toluene

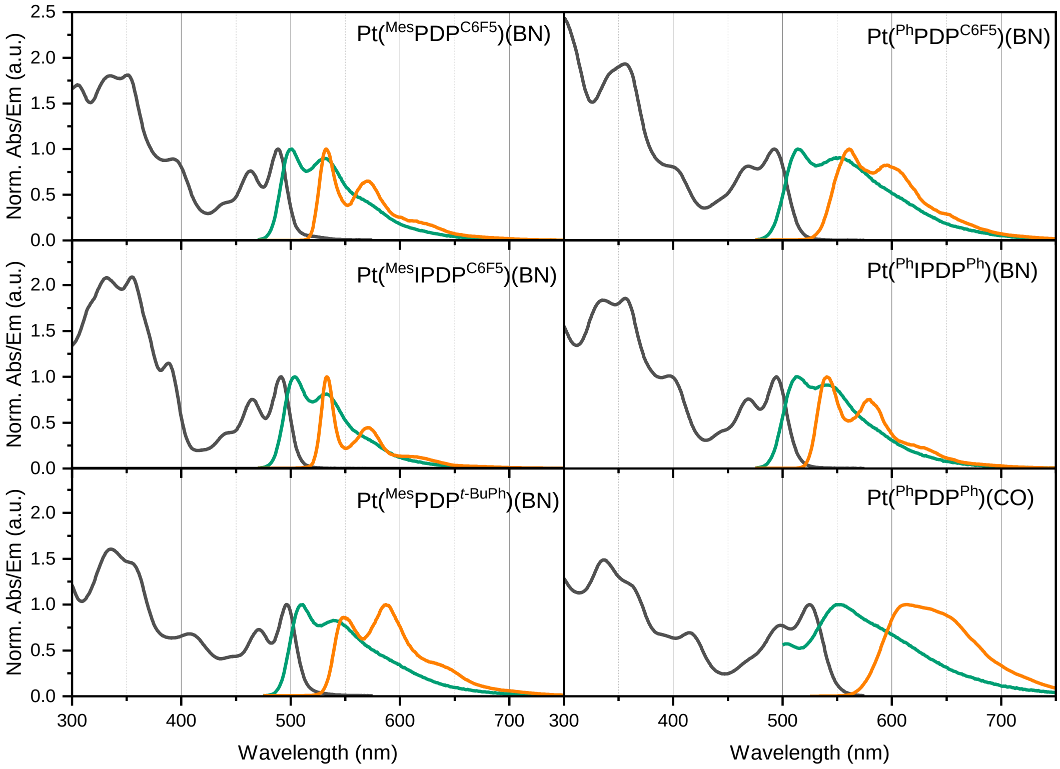

Figure S1. Normalized absorption spectra (in toluene, black lines), emission spectra at room temperature (in anoxic toluene, green lines) and at 77 K (in toluene/THF (4:6 v/v), orange lines).

1.2. Solvatochromism

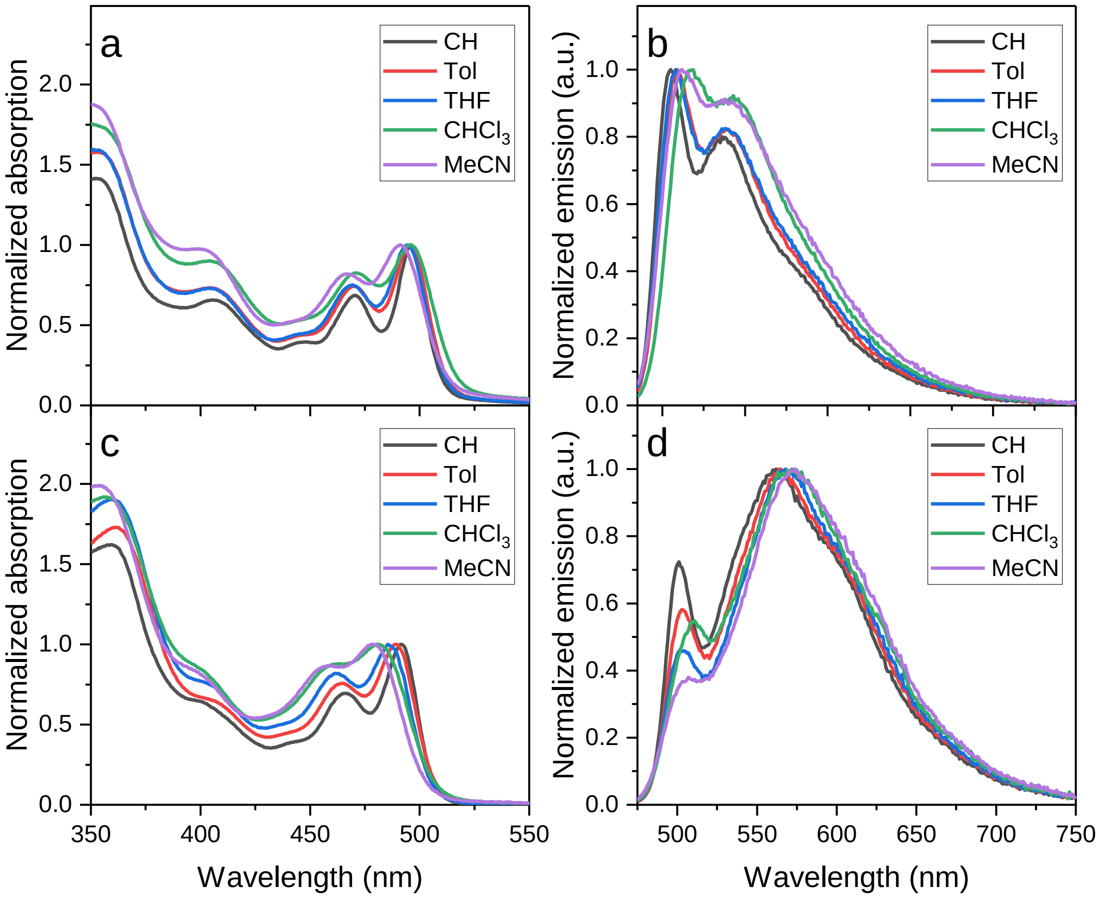

Figure S2. Normalized absorption and emission spectra for (a, b) **Pt(^{Mes}PDP^{Ph})(BN)** and (c, d) **Pt(^{Ph}PDP^{Ph})(Py)** in cyclohexane (CH), toluene (Tol), tetrahydrofuran (THF), chloroform ($CHCl_3$) and acetonitrile (MeCN) at 25 °C (C=10 μM).

1.3. Luminescence decay in toluene

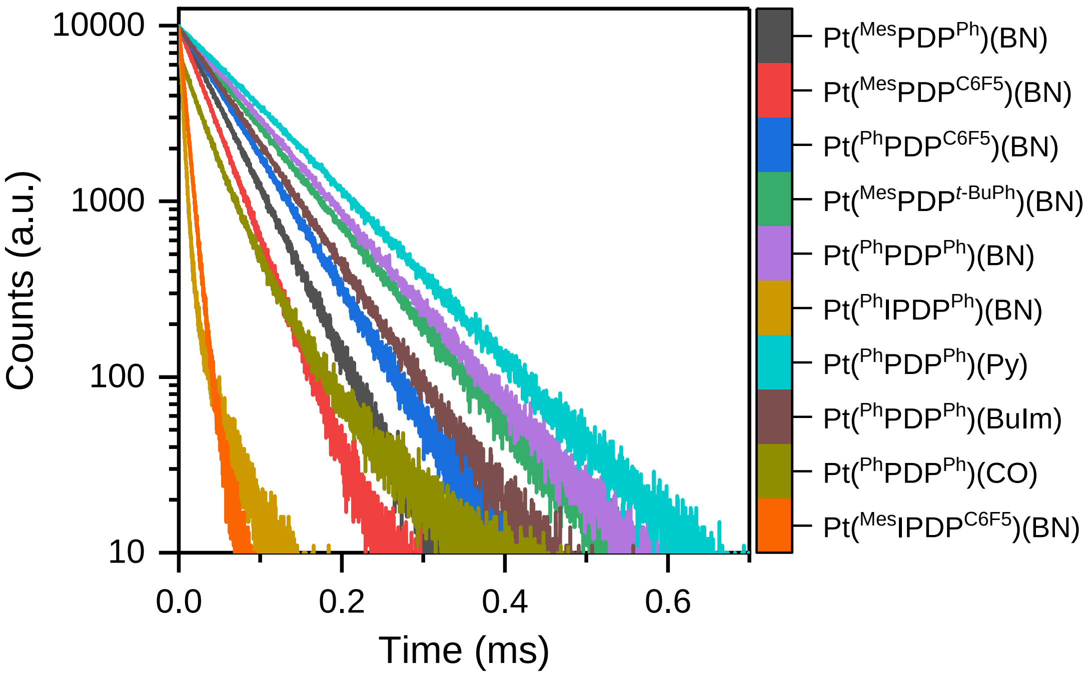

Figure S3. Luminescence decay of platinum(II) complexes in toluene under anoxic conditions at 25 °C. The decays were recorded at the emission maxima of the dyes.

1.4. Photostability in toluene

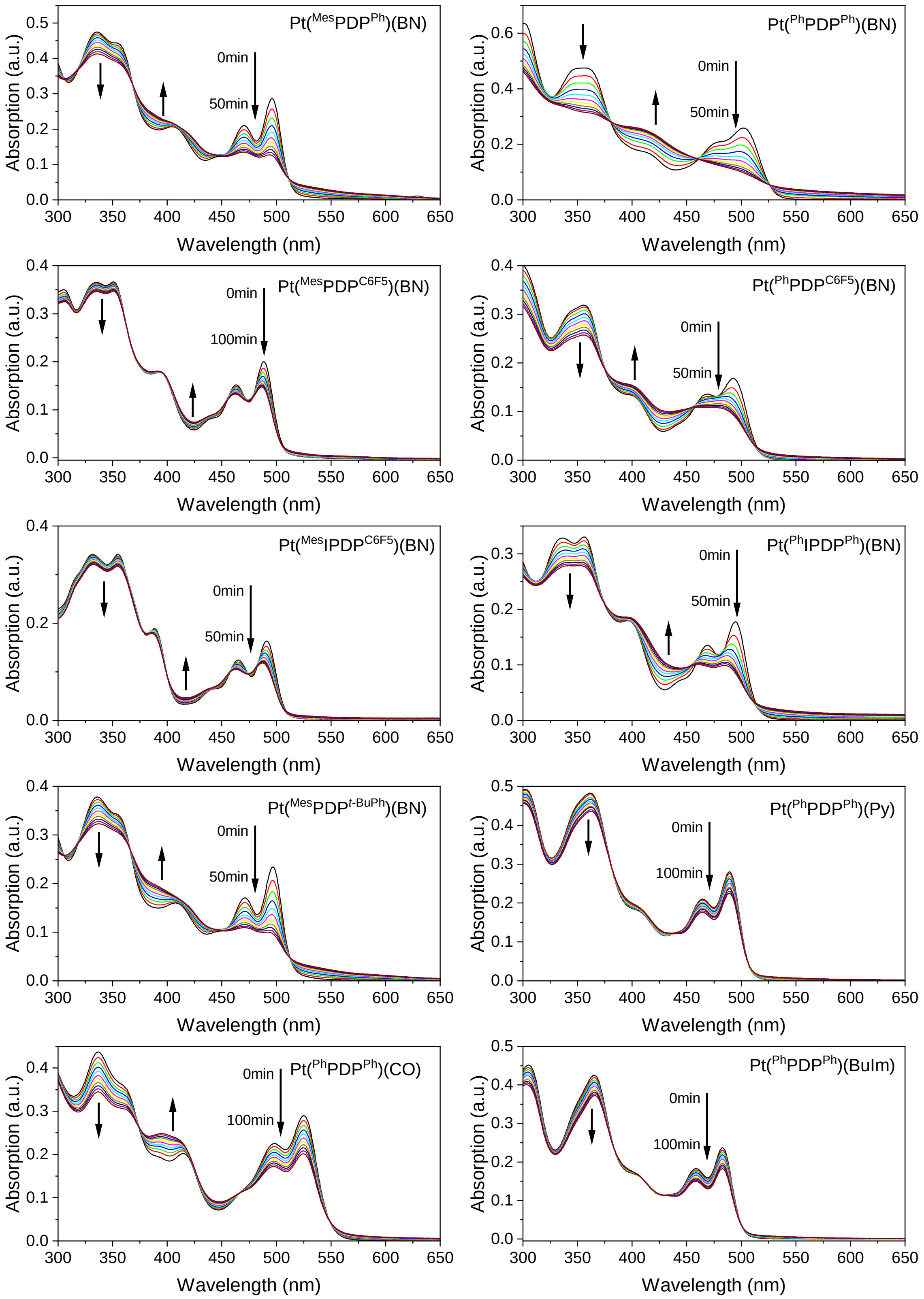

Figure S4. Absorption spectra of platinum(II) complexes in air-saturated toluene over a period of either 50 or 100 min (spectra were recorded every 5 or 10 min). The dye solutions were illuminated with a metal-halide lamp (400-700 nm).

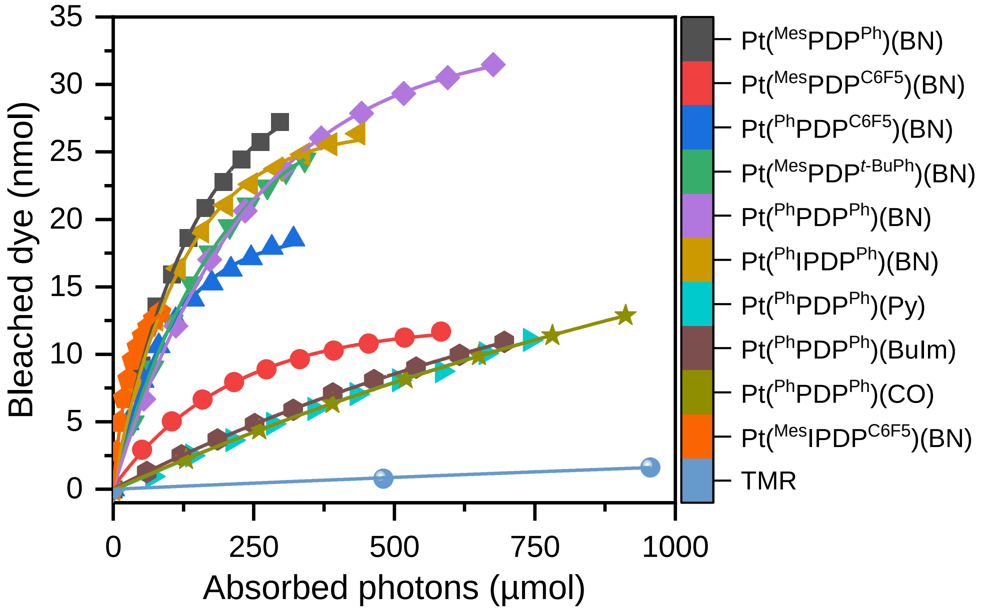

Figure S5. Photodegradation of platinum(II) complexes (in air-equilibrated toluene) and the reference TMR dye (in air-saturated water) upon irradiation with metal-halide lamp (400–700 nm).

2. Computational Methods

All calculations were performed using the quantum chemistry package ORCA (version 5.0.2).[1] Visualization of molecular orbitals was done with Avogadro.[2] Optimized geometries are provided as xyz files.

2.1. Ground-state calculations

Preoptimizations were performed using the B3LYP[3,4] functional, the def2-SVPD[5,6] basis set and D3[7] dispersion correction. The RIJCOSX[8,9] approximation and the def2/J auxiliary basis set were used to accelerate calculations. The conductor-like polarizable continuum model (CPCM)[10] using toluene (ε=2.4, refractive index=1.497) as the solvent was applied in all calculations. Stationary points were confirmed to be true energy minima by calculation of numerical frequencies. Subsequently, the structures were further optimized at triple-zeta level (def2-TZVP). All calculations were performed using the tight SCF settings and a denser grid (defgrid3).

2.2. Excited-state calculations

Excited-state calculations were performed using time-dependent density functional theory (TDDFT) as implemented in ORCA using the abovementioned method. Excited state energy of TDDFT states was calculated using the resulting S_0 geometries (vertical excitations) in line with the procedure presented in former works.[11–14] Additionally, all S_1 and T_1 states have also been optimized. The influence of relativistic effects was also assessed for **Pt(^{Ph}IPDP^{Ph})(BN)**, **Pt(^{Ph}PDP^{Ph})(Py)** and **Pt(^{Mes}PDP^{Ph})(BN)** using the zeroth-order regular approximation (ZORA)[15,16] but was found to be negligible and was therefore not considered for the other structures.

2.3. Results of DFT and TDDFT calculations

Table S1. Calculated HOMO and LUMO energies (E_{HOMO} ,E_{LUMO}), energy gap between HOMO and LUMO (E_{gap}), energies of the first excited singlet and triplet state (E_{S1}, E_{T1}), energy gap between first excited singlet and triplet (ΔE_{ST}) and dihedral angle (Θ) between the PDP ligand and the terminal ligand.

Complex	E_{HOMO} / E_{LUMO} (eV)	E_{gap} (eV)	E_{S1}/E_{T1} vertical (eV)	ΔE_{ST} vertical (eV)	E_{S1} / E_{T1} (eV)	ΔE_{ST} (eV)	Θ (deg)
**Pt(^{Ph}PDP^{Ph})(BN)**	-5.00 / -2.04	2.96	2.48 / 2.23	0.25	2.21/ 2.05	0.16	55
**Pt(^{Ph}PDP^{Ph})(Py)**	-4.97 / -1.75	3.22	2.66 / 2.34	0.32	2.47 / 2.18	0.29	59
**Pt(^{Ph}PDP^{Ph})(BuIm)**	-4.91 / -1.52	3.39	2.79 / 2.38	0.41	2.65 / 2.21	0.44	57
**Pt(^{Ph}PDP^{Ph})(CO)**	-5.22 / -2.15	3.07	2.52 / 2.13	0.39	2.09 / 1.98	0.11	-
**Pt(^{Ph}IPDP^{Ph})(BN)**	-5.17 / -2.02	3.15	2.64 / 2.36	0.28	2.35 / 2.18	0.17	74
**Pt(^{Ph}PDP^{C6F5})(BN)**	-5.31 / -2.17	3.14	2.62 / 2.32	0.29	2.37 / 2.13	0.24	56
**Pt(^{Mes}PDP^{Ph})(BN)**	-4.98 / -1.87	3.11	2.67 / 2.34	0.33	2.32 / 2.19	0.13	83
**Pt(^{Mes}PDP$^{t\text{-}BuPh}$)(BN)**	-4.93 / -1.86	3.07	2.64 / 2.33	0.31	2.30 / 2.18	0.12	82
**Pt(^{Mes}PDP^{C6F5})(BN)**	-5.31 / -2.01	3.30	2.79 / 2.39	0.40	2.51 / 2.24	0.27	90
**Pt(^{Mes}IPDP^{C6F5})(BN)**	-5.41 / 2.03	3.38	2.73 / 2.40	0.33	2.58 / 2.27	0.31	90

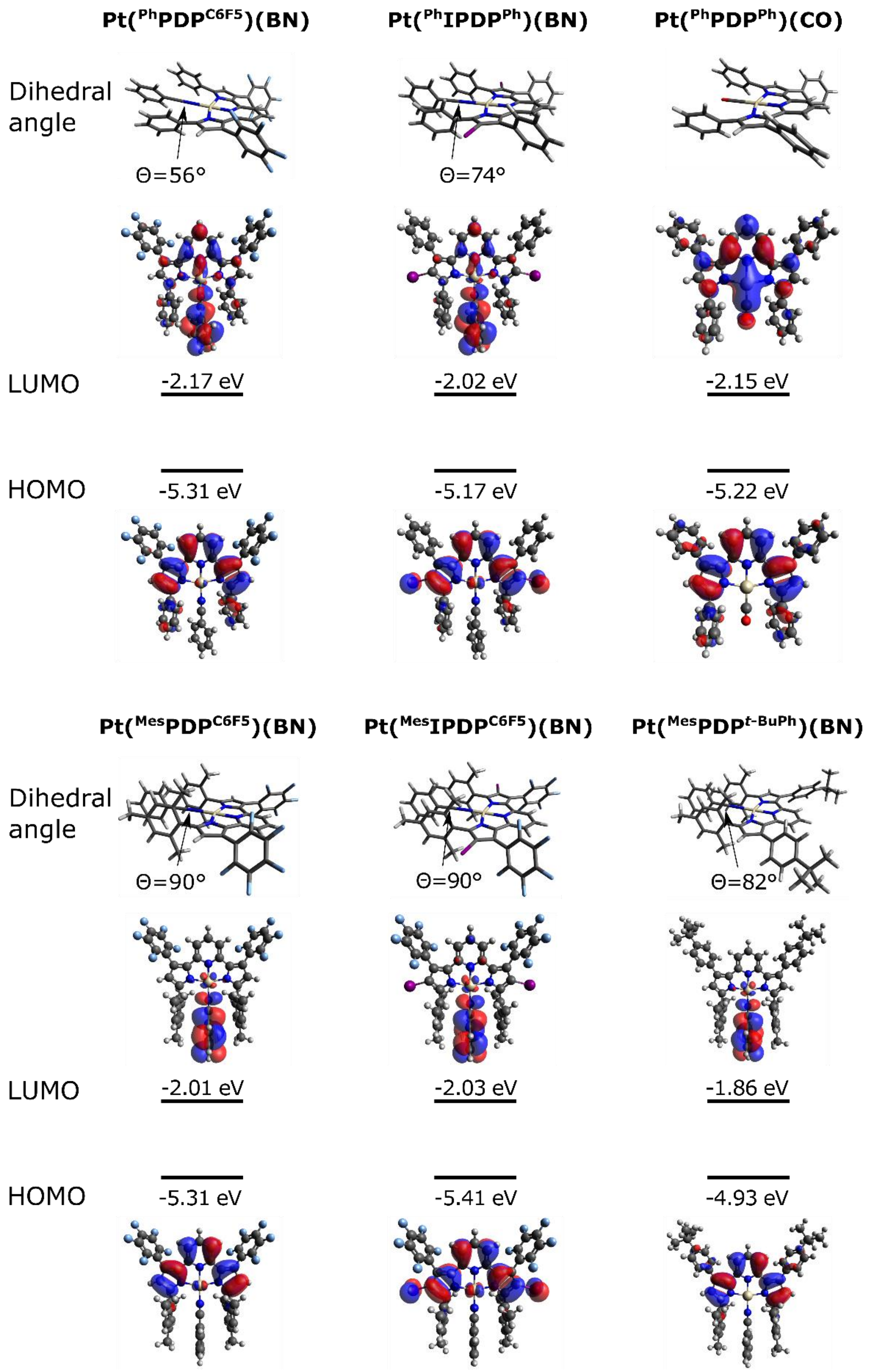

Figure S6. Perspective drawings of the optimized ground state (S_0) geometries with marked dihedral angle θ between donor (PDP ligand) and acceptor (terminal ligand) part and HOMO and LUMO of **Pt(^{Ph}PDP$^{\mathrm{C6F5}}$)(BN)**, **Pt(^{Ph}IPDP^{Ph})(BN)**, **Pt(^{Ph}PDP^{Ph})(CO)**, **Pt(^{Mes}PDP$^{\mathrm{C6F5}}$)(BN)**, **Pt(^{Mes}IPDP$^{\mathrm{C6F5}}$)(BN)** and **Pt(^{Mes}PDP$^{t\text{-}\mathrm{BuPh}}$)(BN)**.

3. Photophysical Properties in PS

3.1.Emission spectra

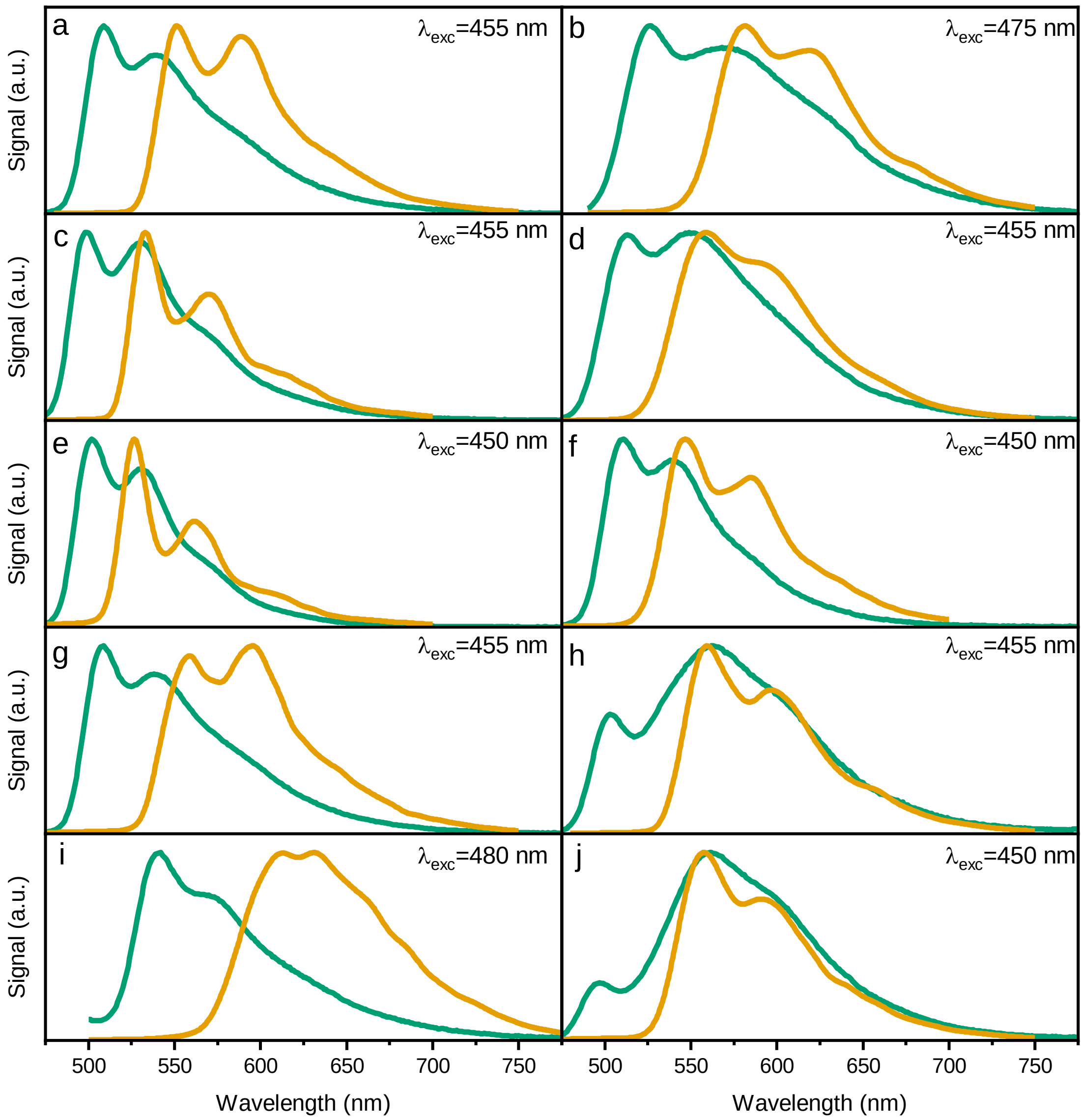

Figure S7. Normalized emission spectra at 25 °C (in 5 wt% Na_2SO_3, green lines) and at 77 K (orange lines) of (a) **Pt(^{Mes}PDP^{Ph})(BN)**, (b) **Pt(^{Ph}PDP^{Ph})(BN)**, (c) **Pt(^{Mes}PDP^{C6F5})(BN)**, (d) **Pt(^{Ph}PDP^{C6F5})(BN)**, (e) **Pt(^{Mes}IPDP^{C6F5})(BN)**, (f) **Pt(^{Ph}IPDP^{C6F5})(BN)**, (g) **Pt(^{Mes}PDP$^{t\text{-}BuPh}$)(BN)**, (h) **Pt(^{Ph}PDP^{Ph})(Py)**, (i) **Pt(^{Ph}PDP^{Ph})(CO)** and (j) **Pt(^{Ph}PDP^{Ph})(BuIm)** immobilized PS.

3.2. Luminescence decay in PS

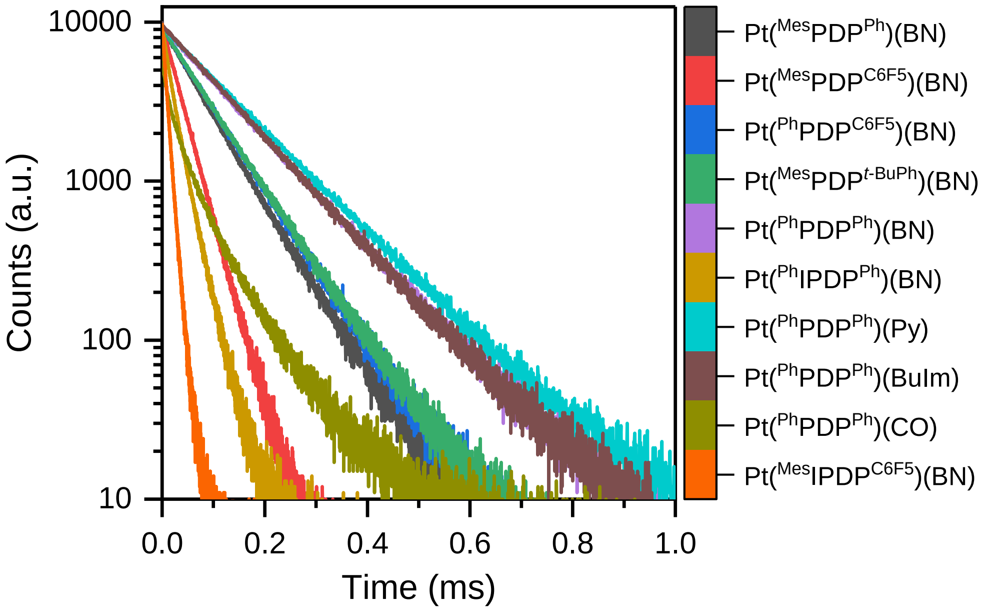

Figure S8. Luminescence decay of platinum(II) complexes immobilized in PS under anoxic conditions at 25 °C. The decays were recorded at the emission maxima of the dyes.

3.3. Photostability in PS

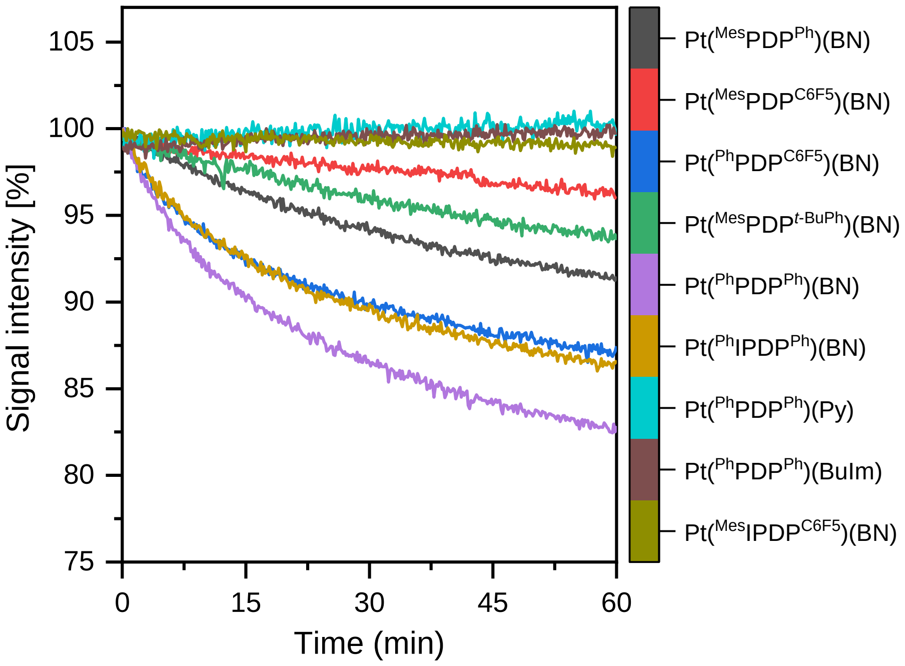

Figure S9. Photodegradation of platinum(II) complexes embedded in PS under anoxic conditions. The complexes where excited at the lowest energy absorption maxima for one hour with a xenon lamp of a Fluorolog-3 spectrometer.

3.4. Temperature stability in PS

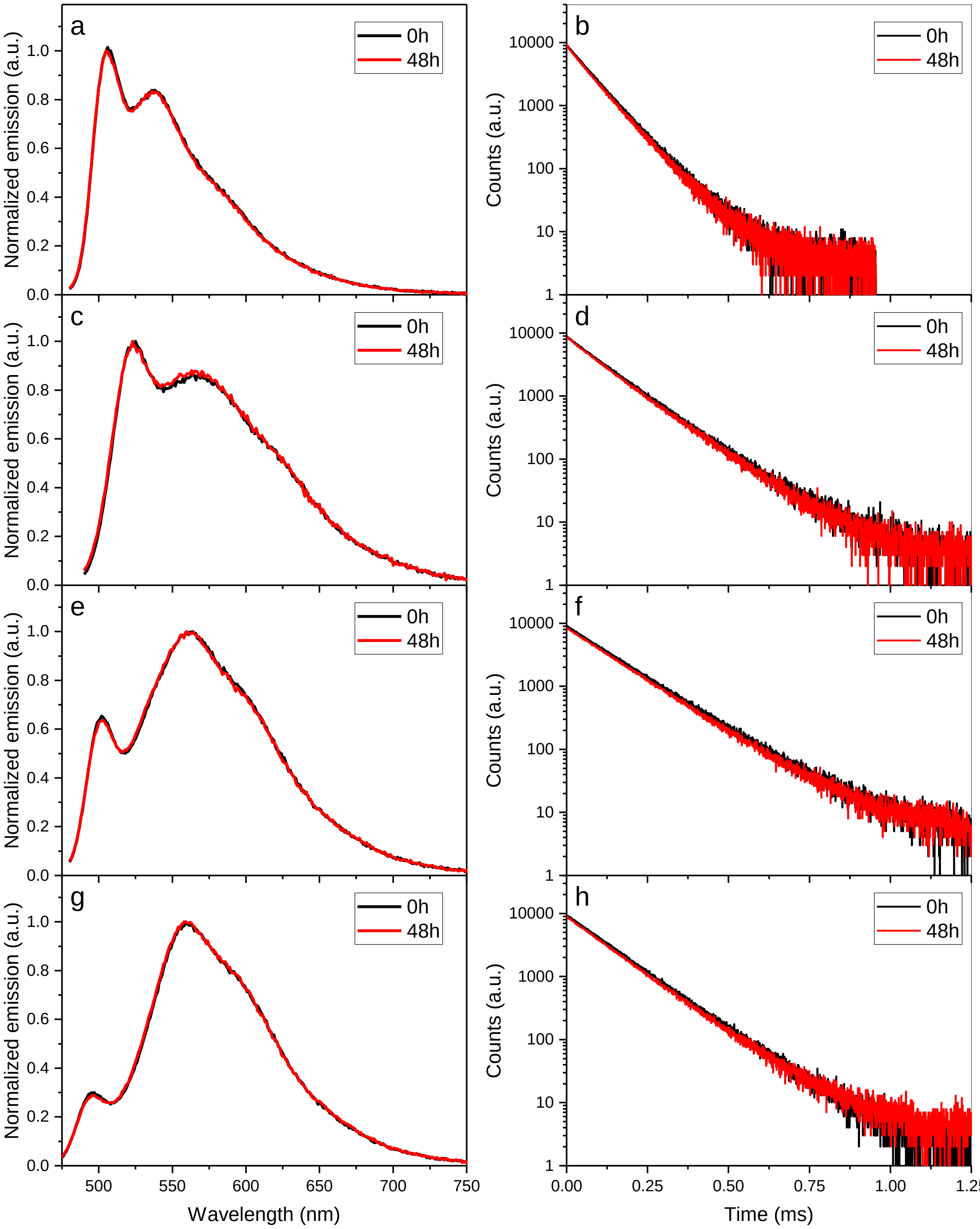

Figure S10. Emission spectra and luminescence decays of (a, b) **Pt(^{Mes}PDP^{Ph})(BN)**, (c, d) **Pt(^{Ph}PDP^{Ph})(BN)**, (e, f) **Pt(^{Ph}PDP^{Ph})(Py)** and (g, h) **Pt(^{Ph}PDP^{Ph})(BuIm)** immobilized in PS before and after being stored in water at 85°C for 48 hours. Emission spectra and luminescence decays were measured under anoxic conditions at 25°C.

3.5.Temperature sensitivity (decay time)

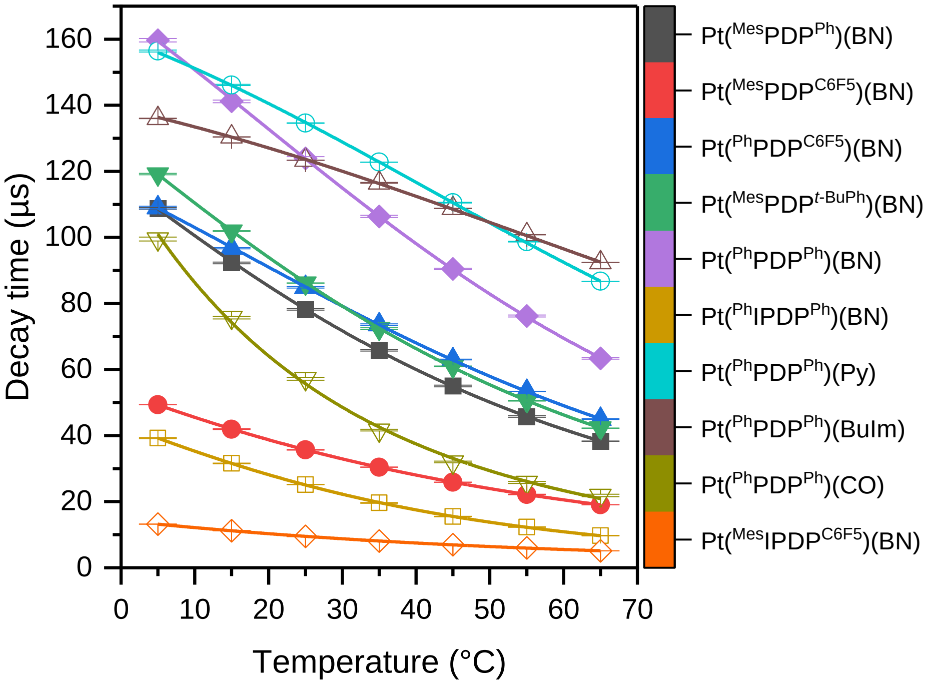

Figure S11. Temperature dependency of decay times for platinum(II) complexes immobilized in PS acquired under anoxic conditions. The complexes were excited with a spectraLED (λ = 392 nm) and the emission decays were acquired at the TADF emission maximum. For the temperature dependency measurements, three measurement cycles were performed.

3.6. Deconvolution of emission spectra of Pt(II) complexes embedded in PS

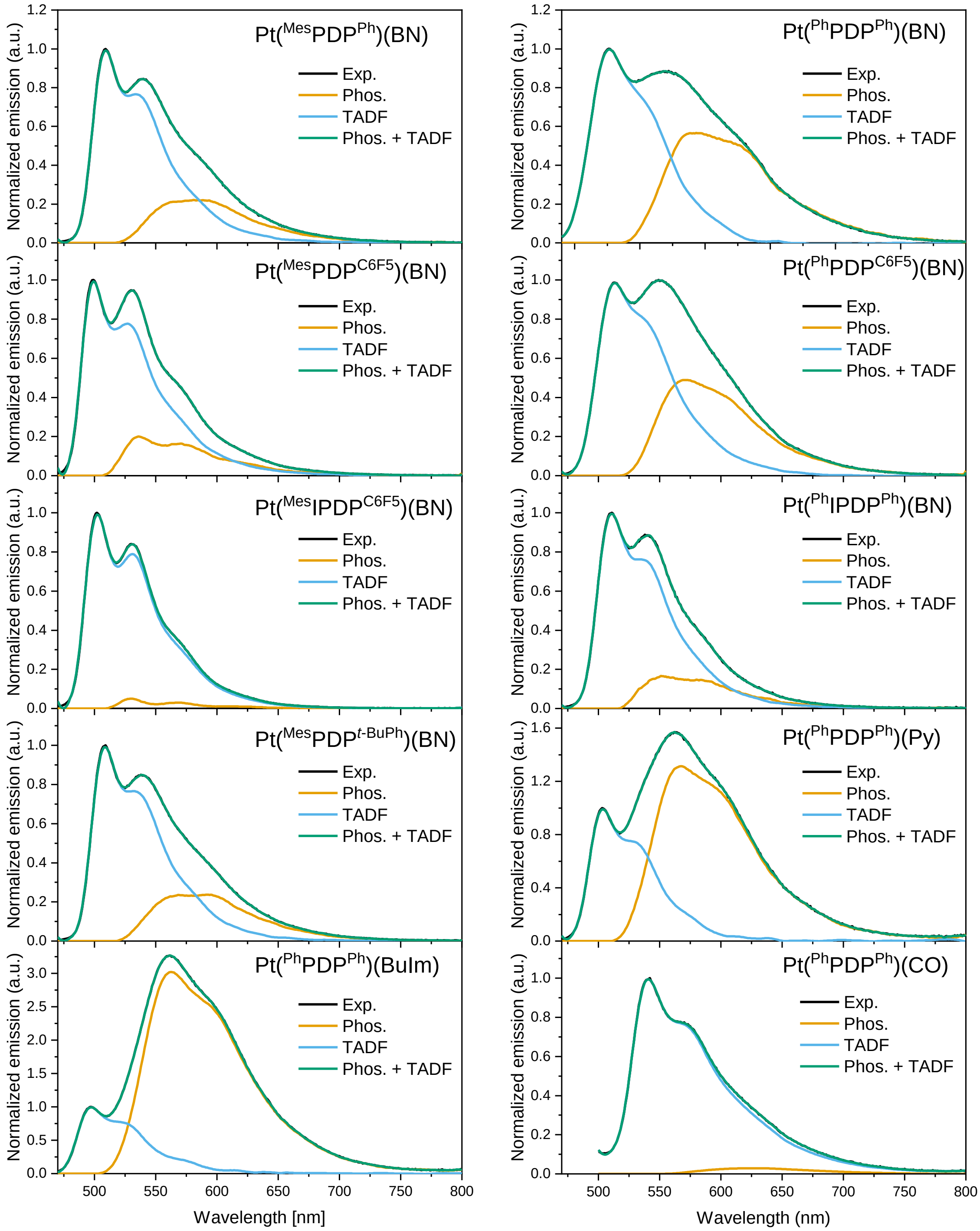

Figure S12. Deconvoluted emission spectra of platinum(II) complexes in PS. Exp.: experimental spectrum at 25 °C; Phos.: deconvoluted phosphorescence spectrum; TADF: deconvoluted TADF spectrum; Phos. + TADF: sum of deconvoluted phosphorescence and TADF spectra.

3.7. Contributions of TADF and phosphorescence

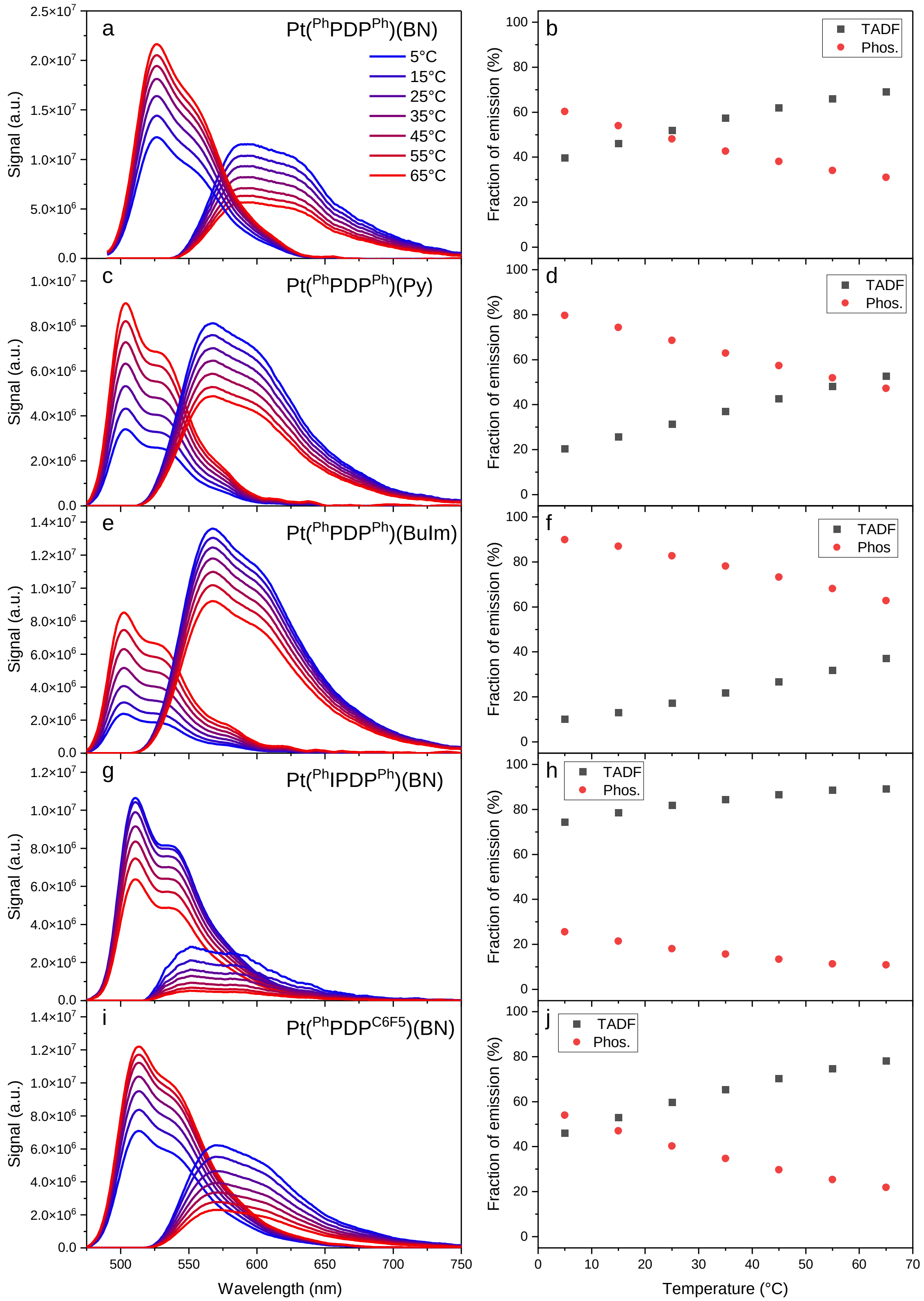

Figure S13. Deconvoluted emission spectra of platinum(II) complexes in PS recorded between 5 °C and 65 °C and calculated fraction of TADF and phosphorescence for (a, b) **Pt(^{Ph}PDP^{Ph})(BN)**, (c, d) **Pt(^{Ph}PDP^{Ph})(Py)**, (e, f) **Pt(^{Ph}PDP^{Ph})(BuIm)**, (g, h) **Pt(^{Ph}IPDP^{Ph})(BN)** and (i, j) **Pt(^{Ph}PDP^{C6F5})(BN)**.

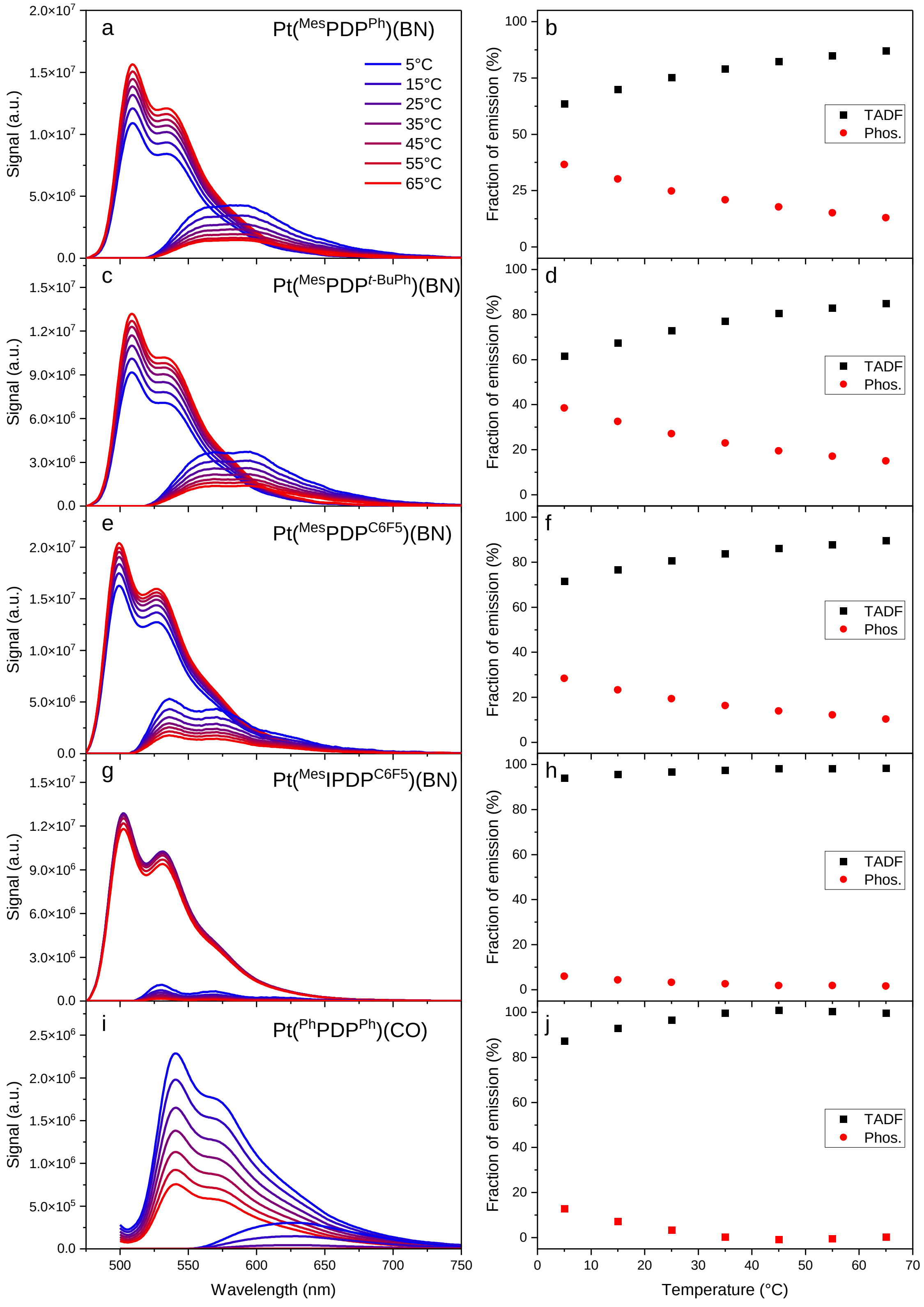

Figure S14. Deconvoluted emission spectra of platinum(II) complexes in PS recorded between 5 °C and 65 °C and calculated fraction of TADF and phosphorescence for (a, b) **Pt(^{Mes}PDP^{Ph})(BN)**, (c, d) **Pt(^{Mes}PDP$^{t\text{-}BuPh}$)(BN)**, (e, f) **Pt(^{Mes}PDP^{C6F5})(BN)**, (g, h) **Pt(^{Mes}IPDP^{C6F5})(BN)** and (i, j) **Pt(^{Ph}PDP^{Ph})(CO)**.

3.8. Temperature sensitivity (ratiometric read-out)

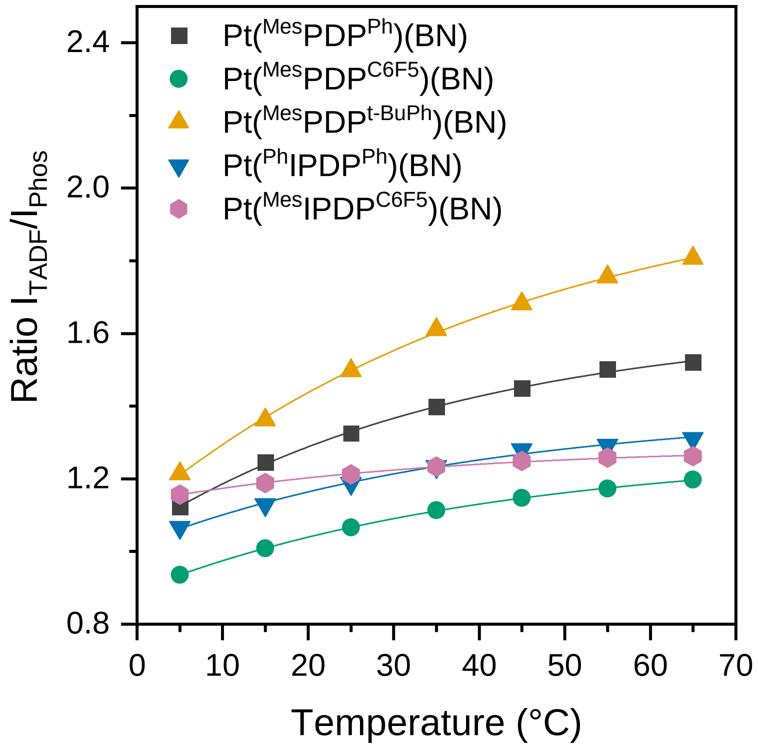

Figure S15. Temperature response on the ratio between TADF and phosphorescence intensities. The intensities were measured at the TADF and phosphorescence emission maxima (Table 2, manuscript).

4. Applications

4.1. Ratiometric readout of planar PAN sensor foils

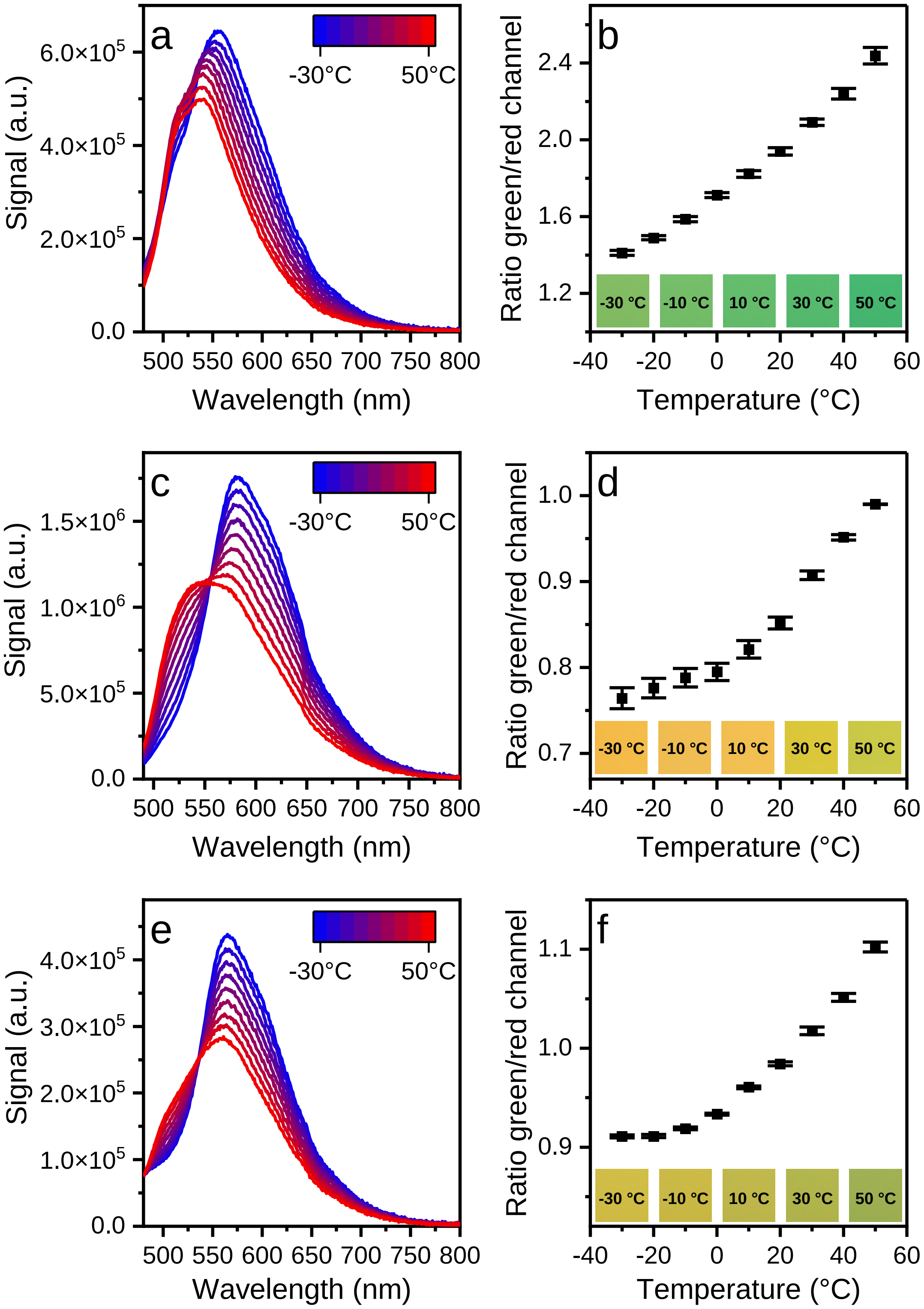

Figure S16. Temperature dependence of (a, c, e) emission spectra and (b, d, f) green/red channel ratios of the photographic images for (a, b) **Pt(^{Mes}PDP^{Ph})(BN)**, (c, d) **Pt(^{Ph}PDP^{Ph})(BN)** and (e, f) **Pt(^{Ph}PDP^{Ph})(BuIm)** immobilized in PAN. The images in (b, d, f) show RGB images taken at the respective temperature. The spectra were recorded with a Fluorolog-3 spectrometer and the images were taken with an RGB camera (Sony Alpha 6000).

4.2. Temperature Nanosensors

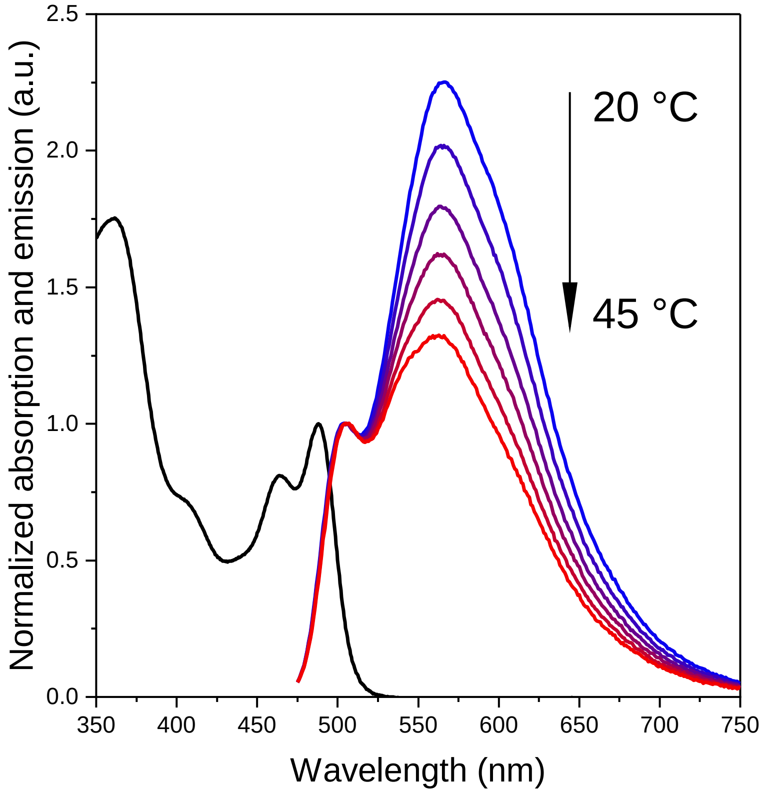

Figure S17. Normalized absorption spectra (black line) and emission spectra between 20 °C and 45 °C (5 °C steps, red and blue lines) of **Pt(^{Ph}PDP^{Ph})(Py)** embedded in RL100-based nanoparticles, measured in aerated water. For recording the emission spectra, the particles were excited at 455 nm.

5. NMR Spectra

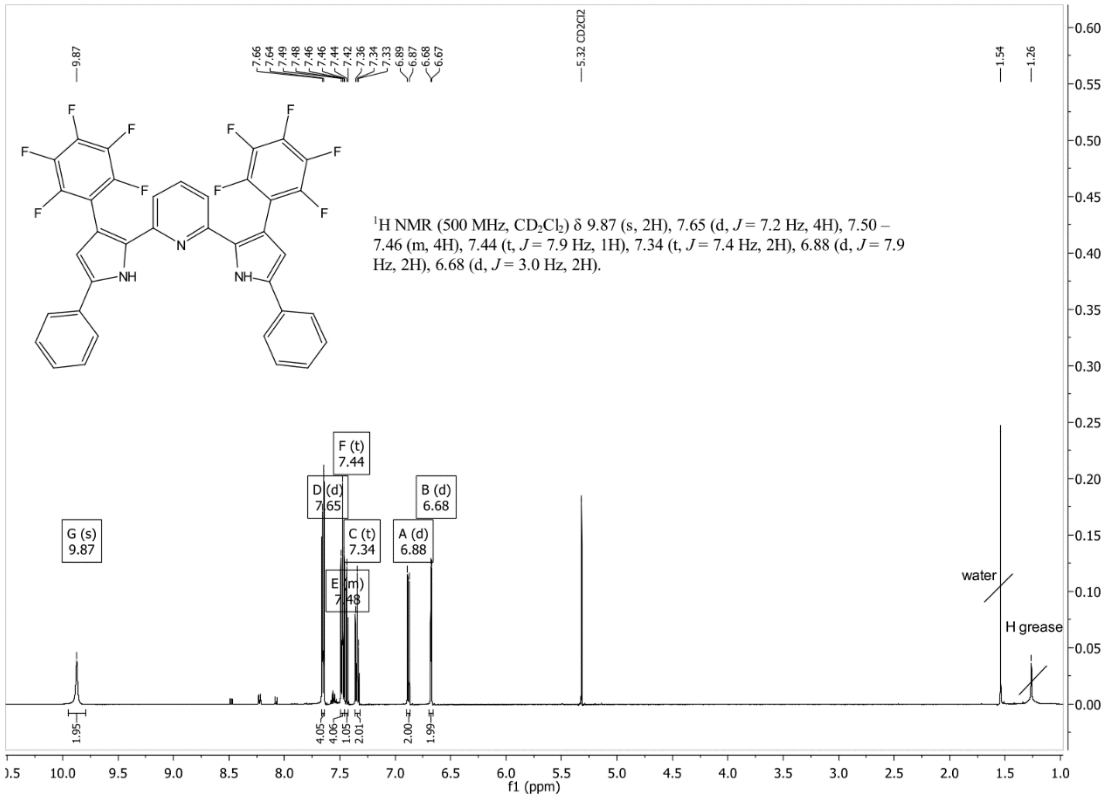

Figure S18. ^{1}H NMR spectrum of $\mathbf{H_2{}^{Ph}PDP^{C6F5}}$ in CD_2Cl_2.

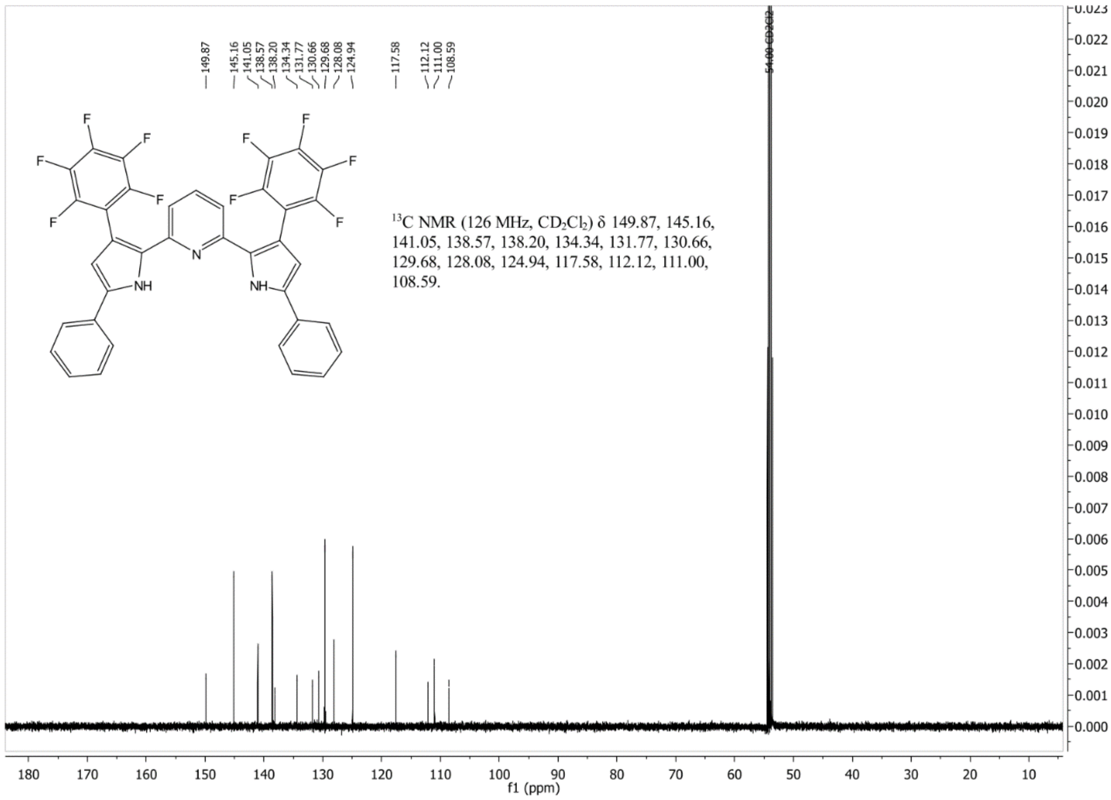

Figure S19. $^{13}C\{^{1}H\}\{^{19}F\}$ NMR spectrum of $\mathbf{H_2{}^{Ph}PDP^{C6F5}}$ in CD_2Cl_2.

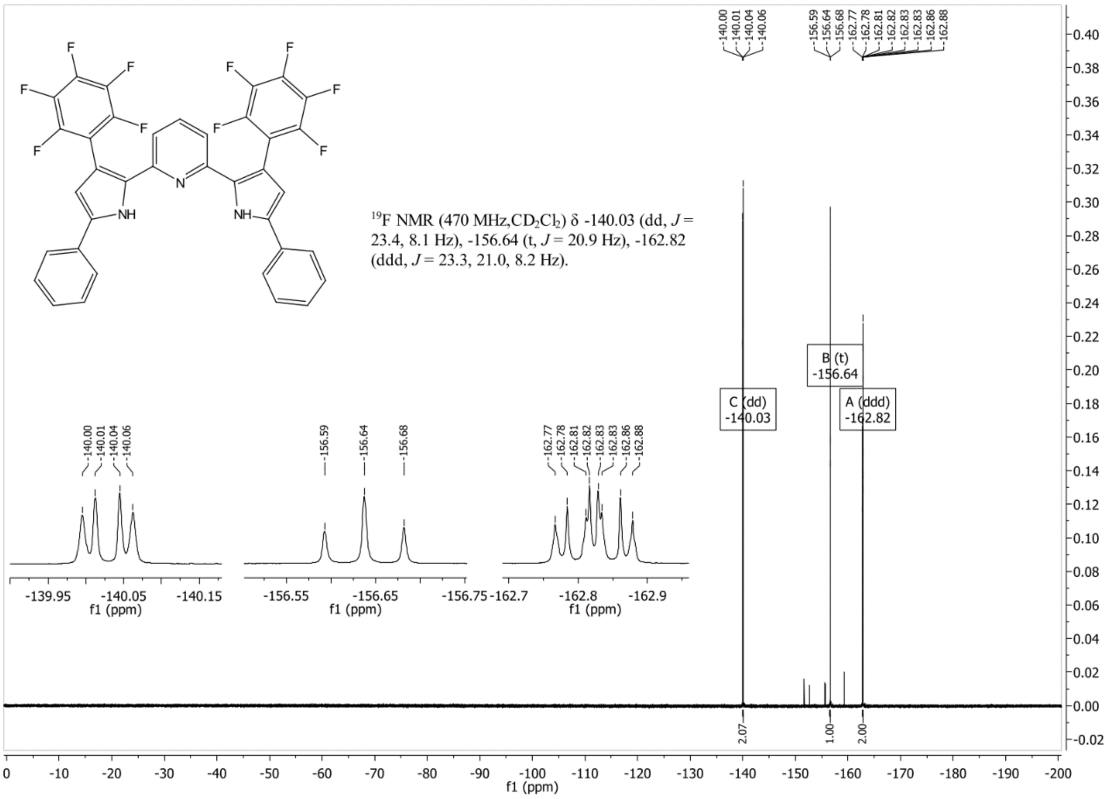

Figure S20. ^{19}F NMR spectrum of $\mathbf{H_2{}^{Ph}PDP^{C6F5}}$ in CD_2Cl_2.

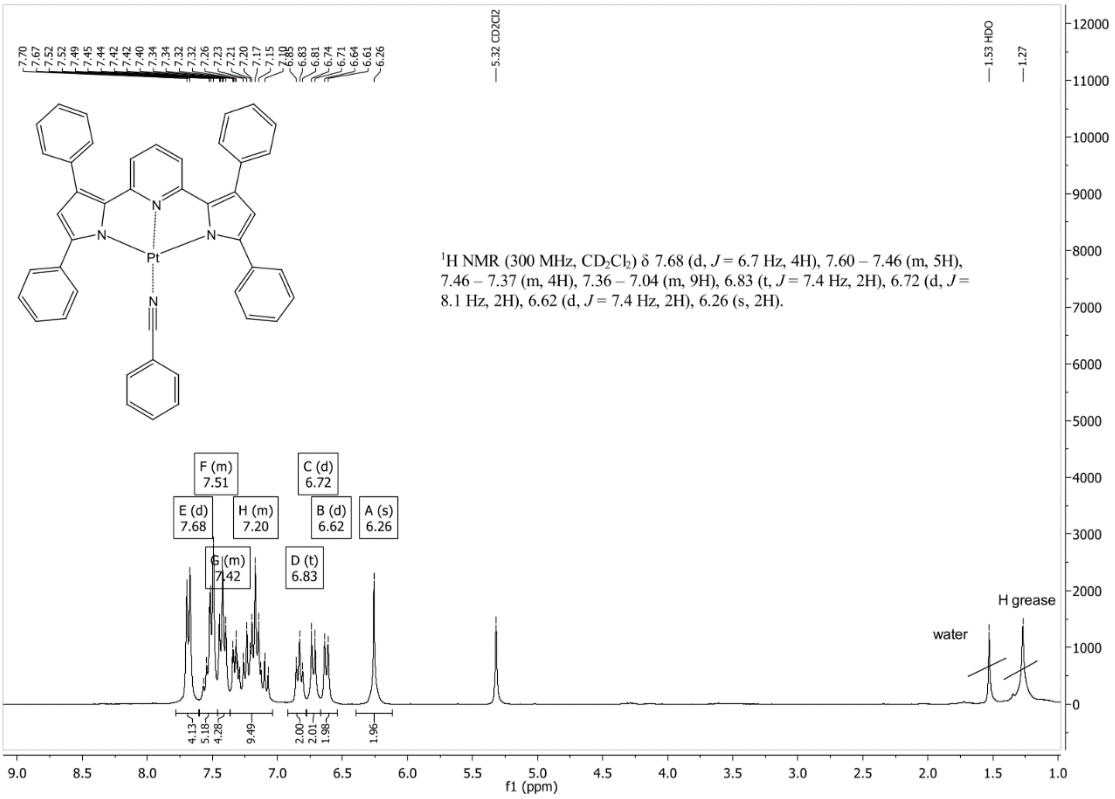

Figure S21. ^{1}H NMR spectrum of **Pt(^{Ph}PDP^{Ph})(BN)** in CD_2Cl_2.

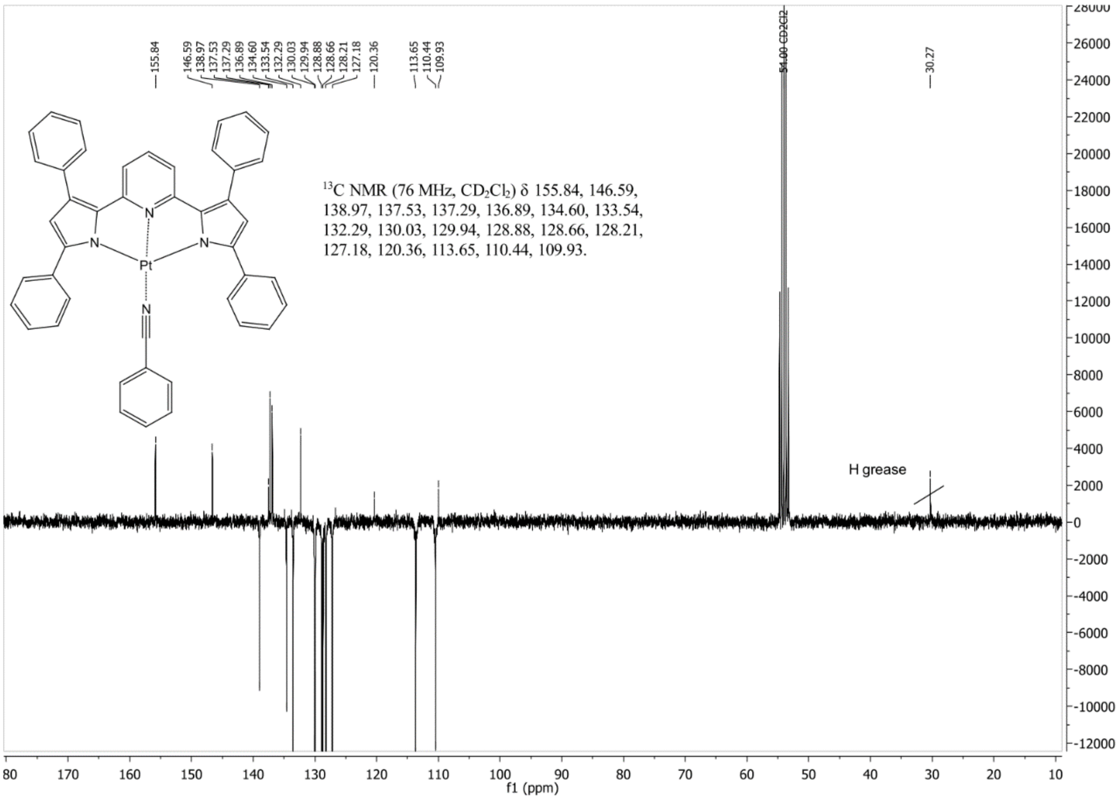

Figure S22. ^{13}C APT NMR spectrum of **Pt(^{Ph}PDP^{Ph})(BN)** in CD_2Cl_2.

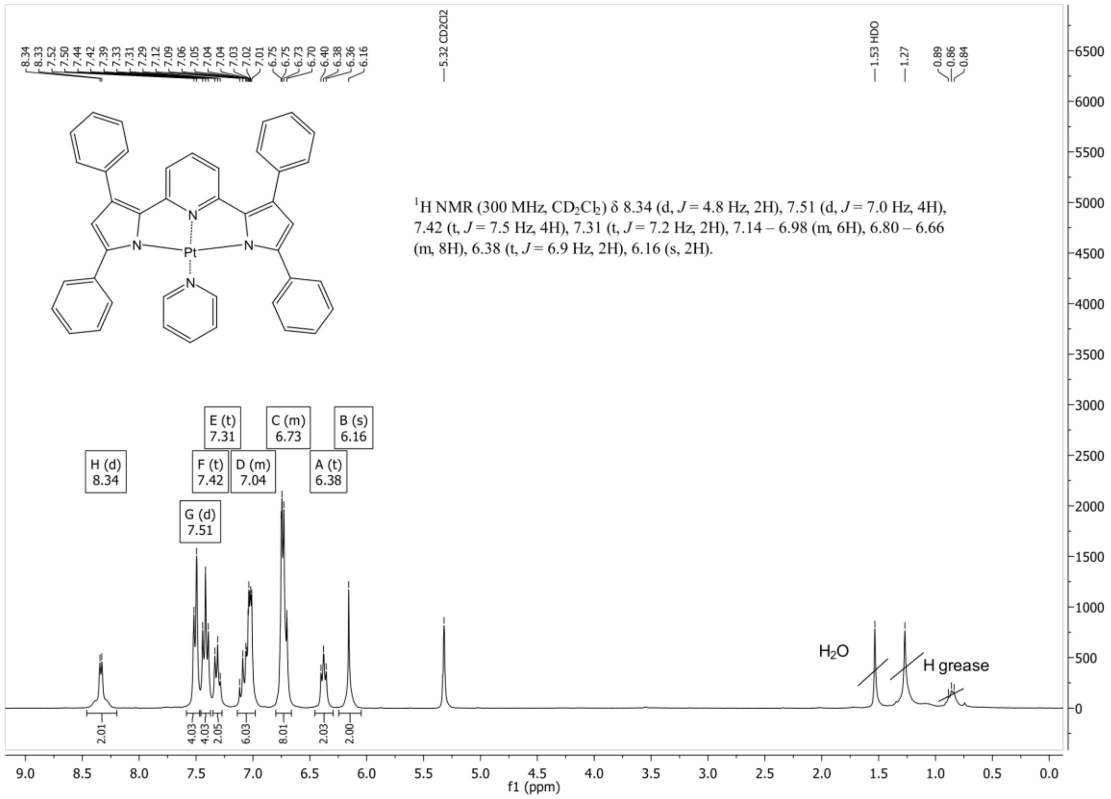

Figure S23. ^{1}H NMR spectrum of **Pt(^{Ph}PDP^{Ph})(Py)** in CD_2Cl_2.

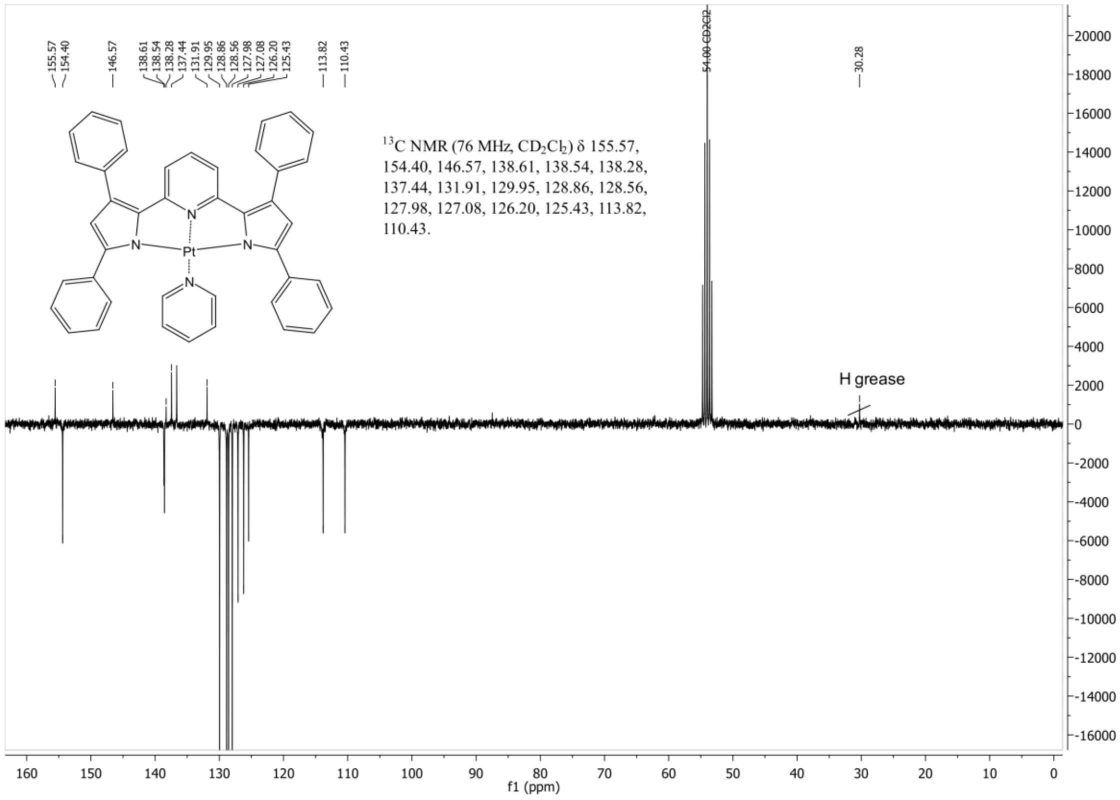

Figure S24. ^{13}C APT NMR spectrum of **Pt(^{Ph}PDP^{Ph})(Py)** in CD_2Cl_2.

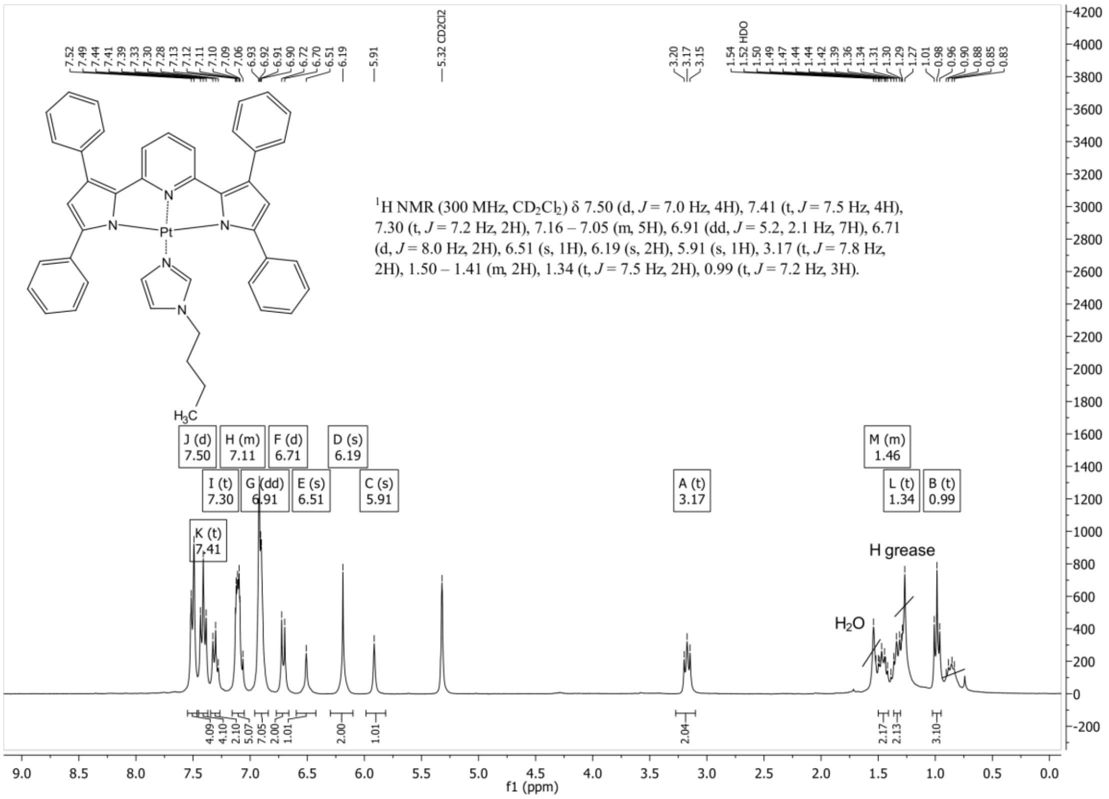

Figure S25. ^{1}H NMR spectrum of **Pt(^{Ph}PDP^{Ph})(BuIm)** in CD_2Cl_2.

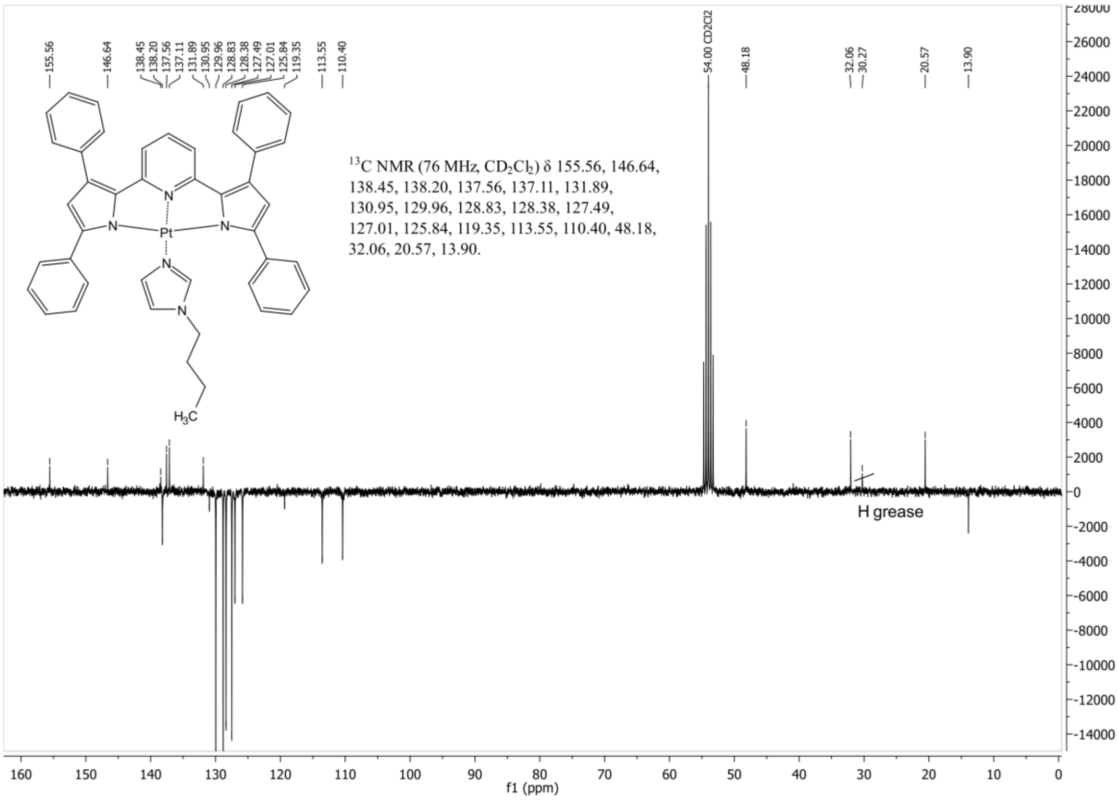

Figure S26. ^{13}C APT NMR spectrum of **Pt(^{Ph}PDP^{Ph})(BuIm)** in CD_2Cl_2.

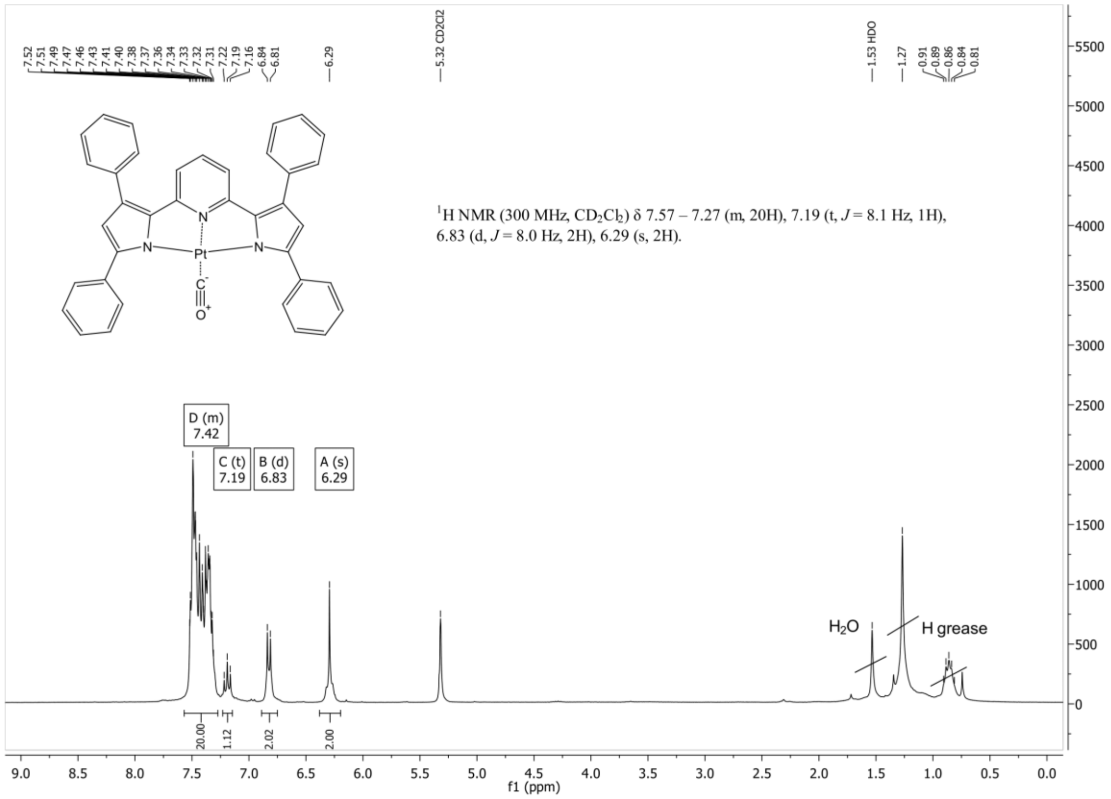

Figure S27. ^{1}H NMR spectrum of **Pt(^{Ph}PDP^{Ph})(CO)** in CD_2Cl_2.

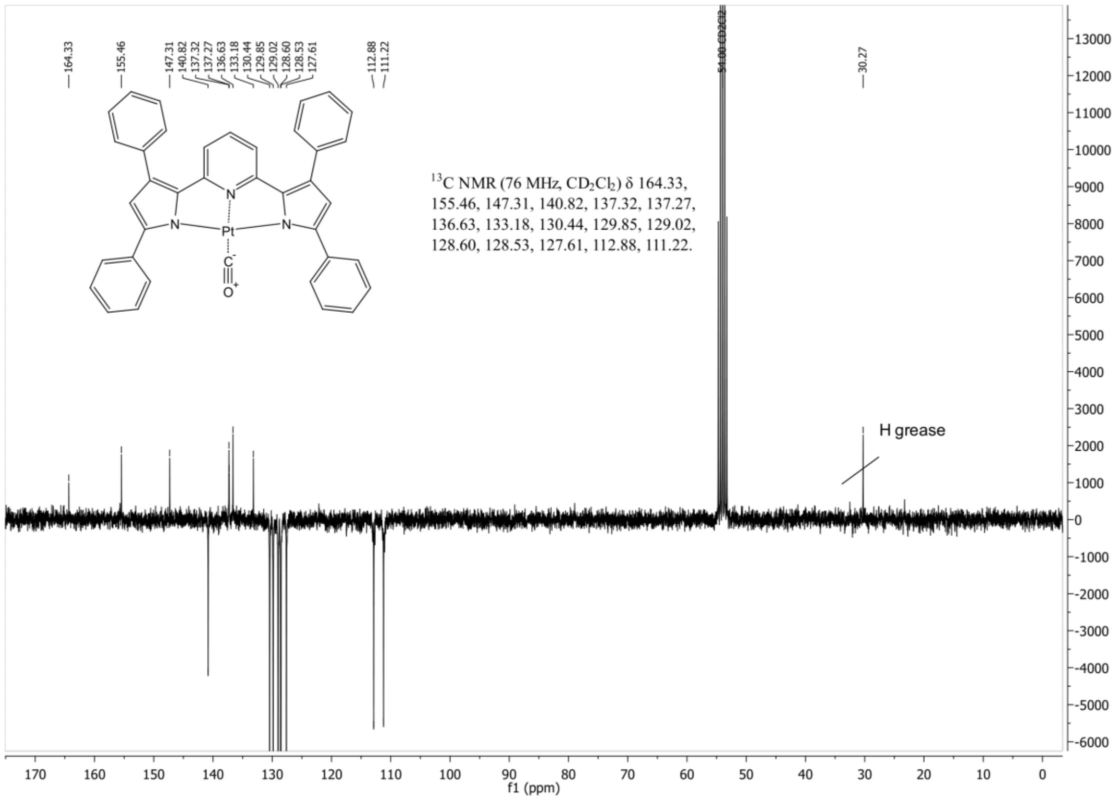

Figure S28. ^{13}C APT NMR spectrum of **Pt(^{Ph}PDP^{Ph})(CO)** in CD_2Cl_2.

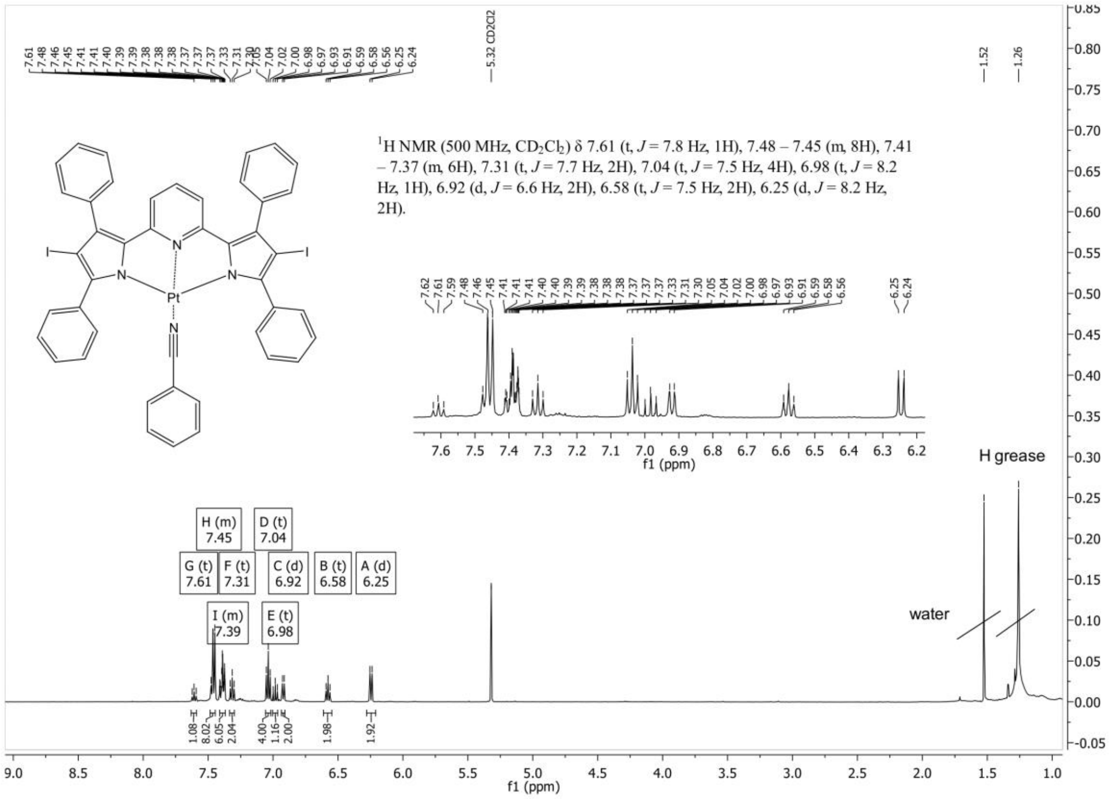

Figure S29. ^{1}H NMR spectrum of **Pt(^{Ph}IPDP^{Ph})(BN)** in CD_2Cl_2.

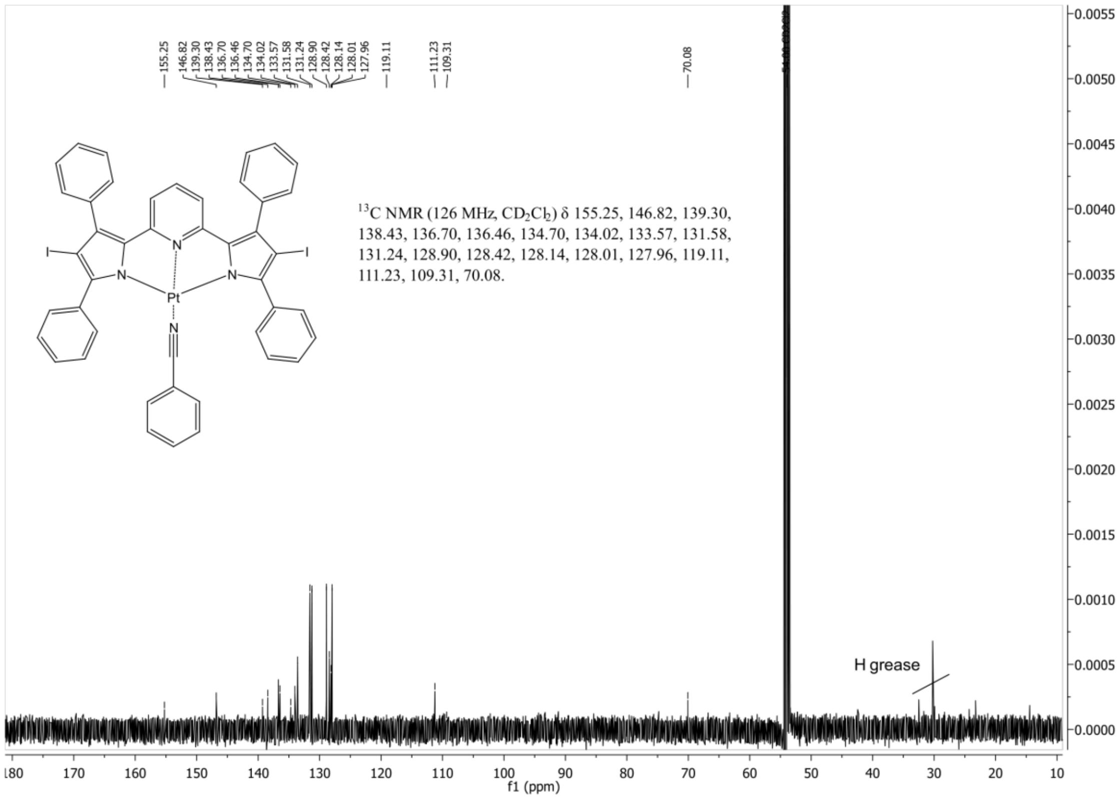

Figure S30. ^{13}C NMR spectrum of **Pt(^{Ph}IPDP^{Ph})(BN)** in CD_2Cl_2.

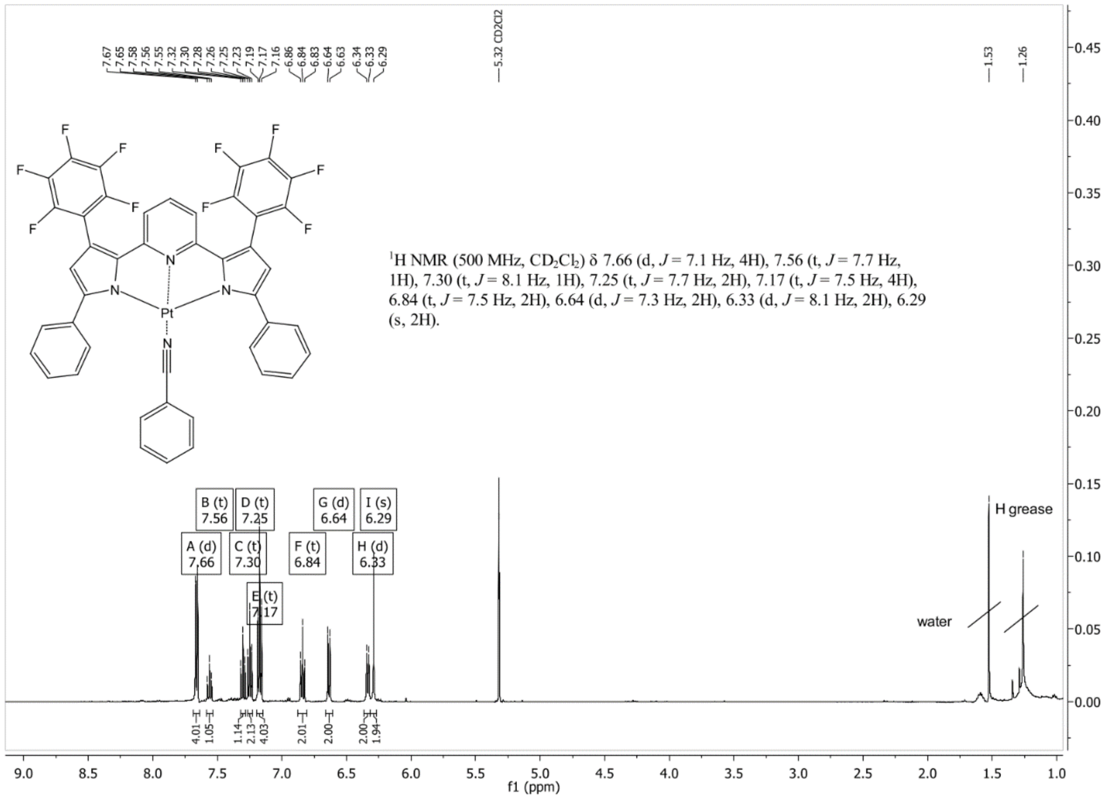

Figure S31. ^{1}H NMR spectrum of **Pt(^{Ph}PDP^{C6F5})(BN)** in CD_2Cl_2.

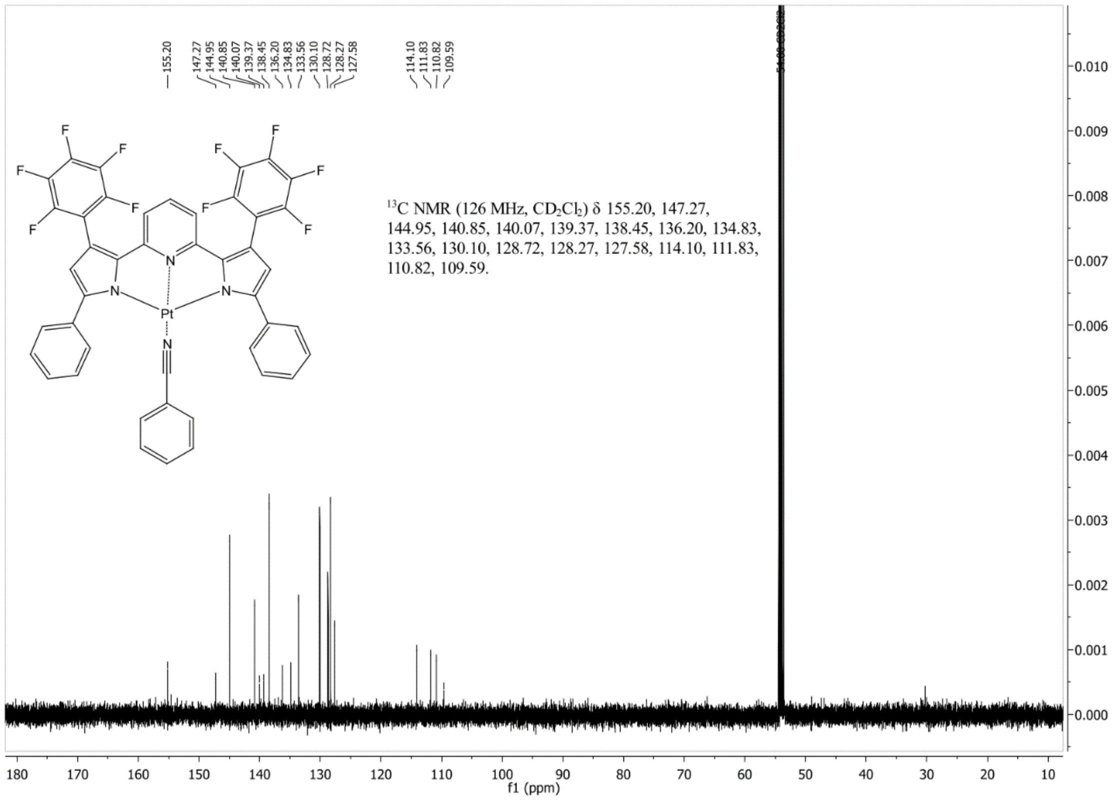

Figure S32. $^{13}C\{^{1}H\}\{^{19}F\}$ NMR spectrum of **Pt(^{Ph}PDP^{C6F5})(BN)** in CD_2Cl_2.

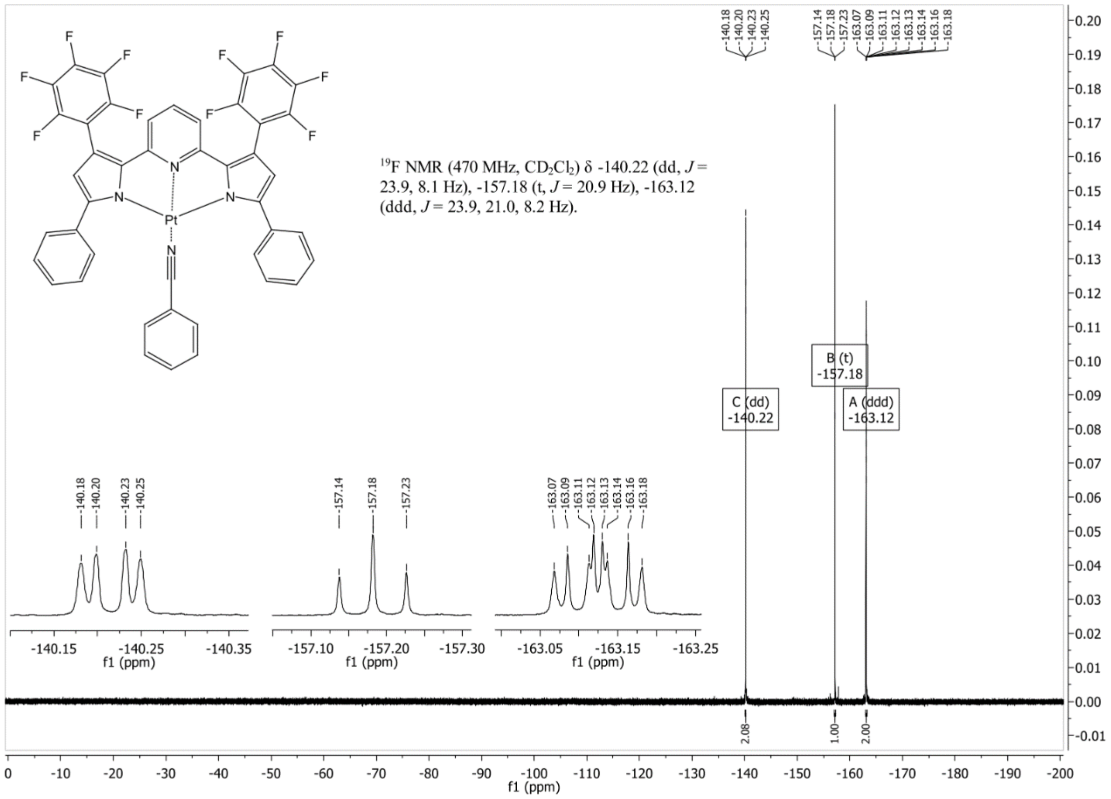

Figure S33. ^{19}F NMR spectrum of **Pt(^{Ph}PDP^{C6F5})(BN)** in CD_2Cl_2.

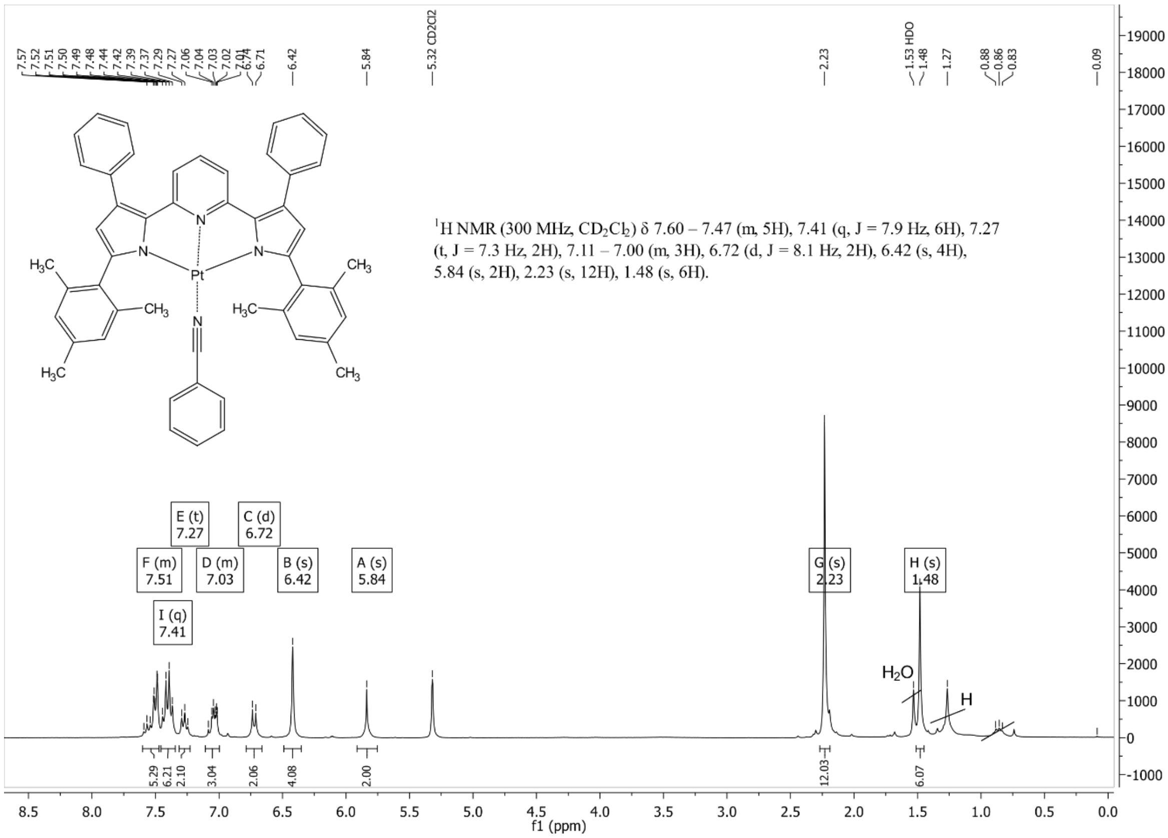

Figure S34. ^{1}H NMR spectrum of **Pt(^{Mes}PDP^{Ph})(BN)** in CD_2Cl_2.

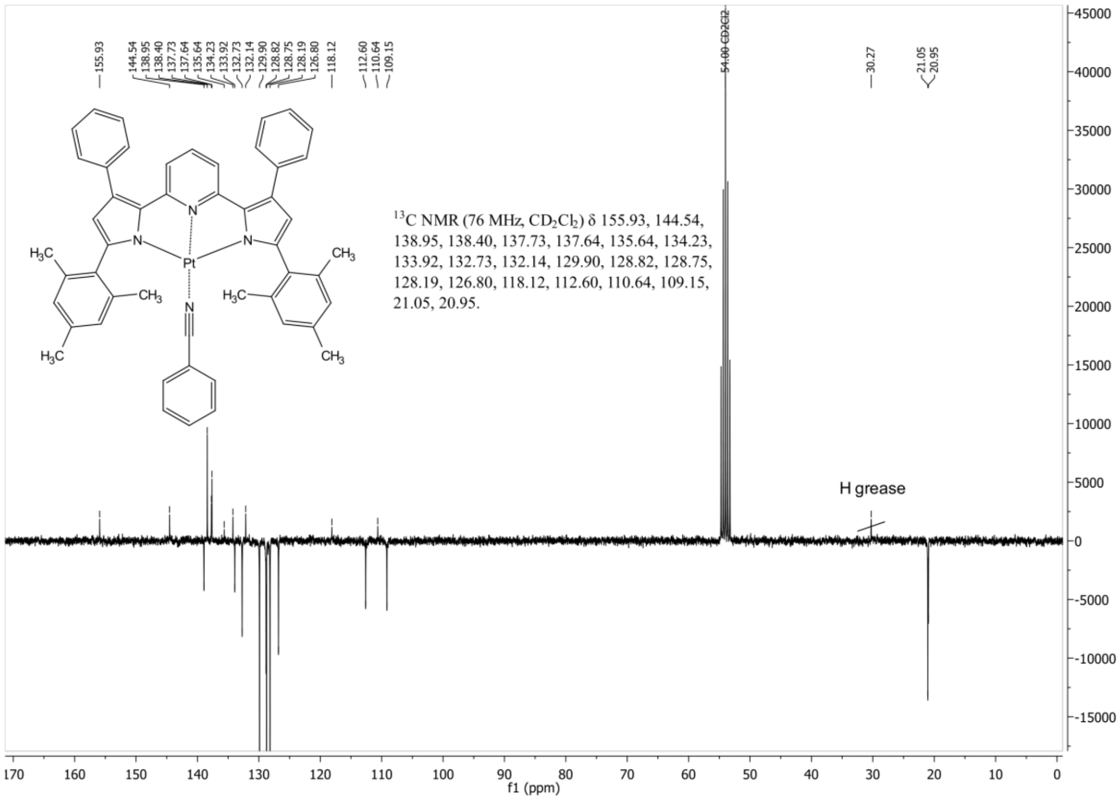

Figure S35. ^{13}C APT NMR spectrum of **Pt(^{Mes}PDP^{Ph})(BN)** in CD_2Cl_2.

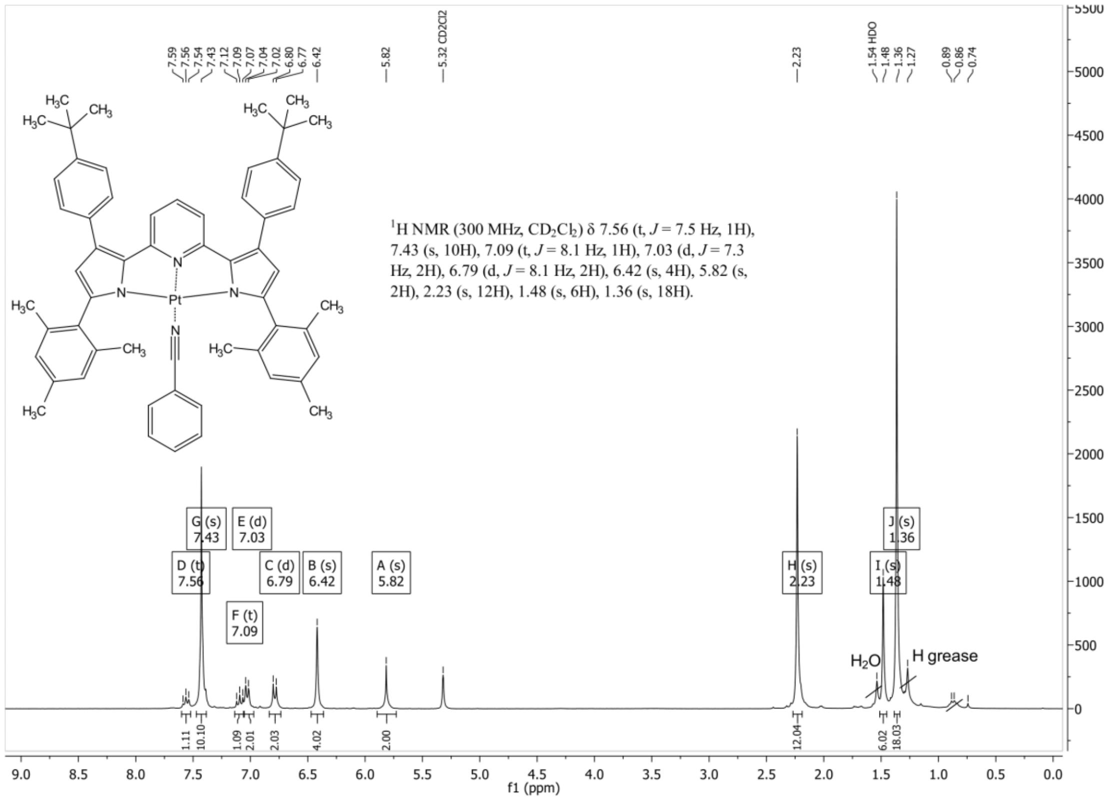

Figure S36. ^{1}H NMR spectrum of **Pt(^{Mes}PDP$^{t\text{-}BuPh}$)(BN)** in CD_2Cl_2.

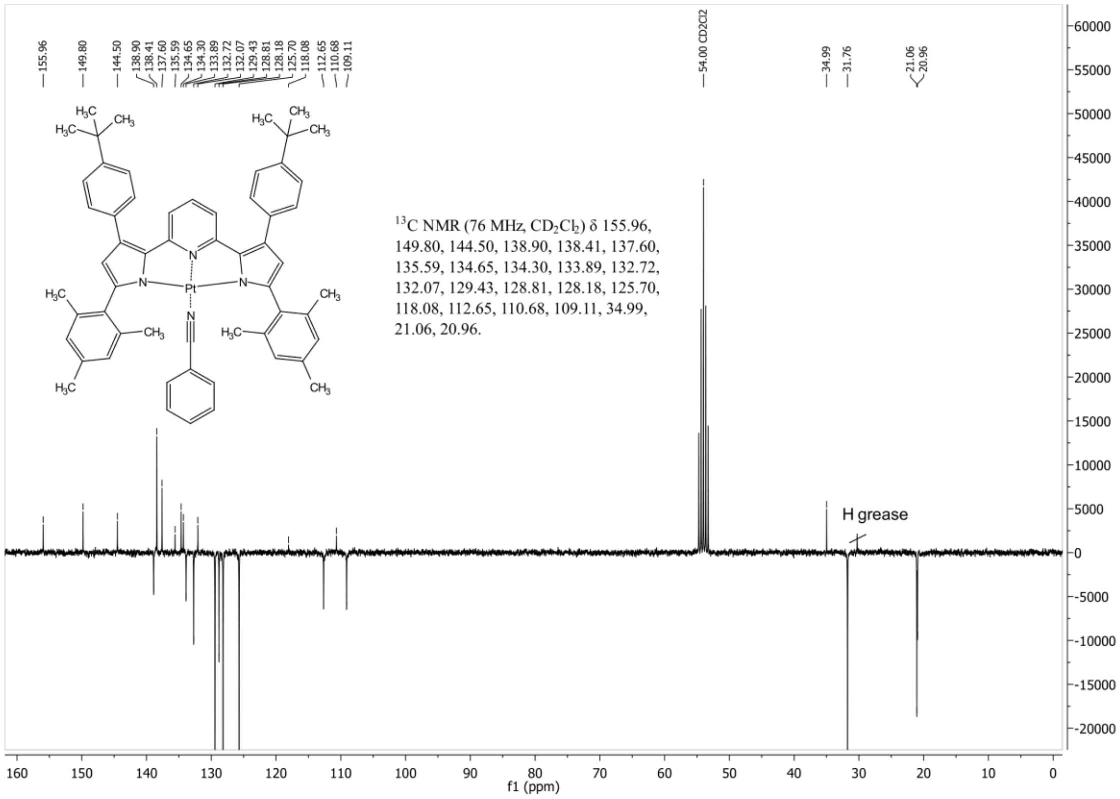

Figure S37. ^{13}C APT NMR spectrum of **Pt(^{Mes}PDP$^{t\text{-}BuPh}$)(BN)** in CD_2Cl_2.

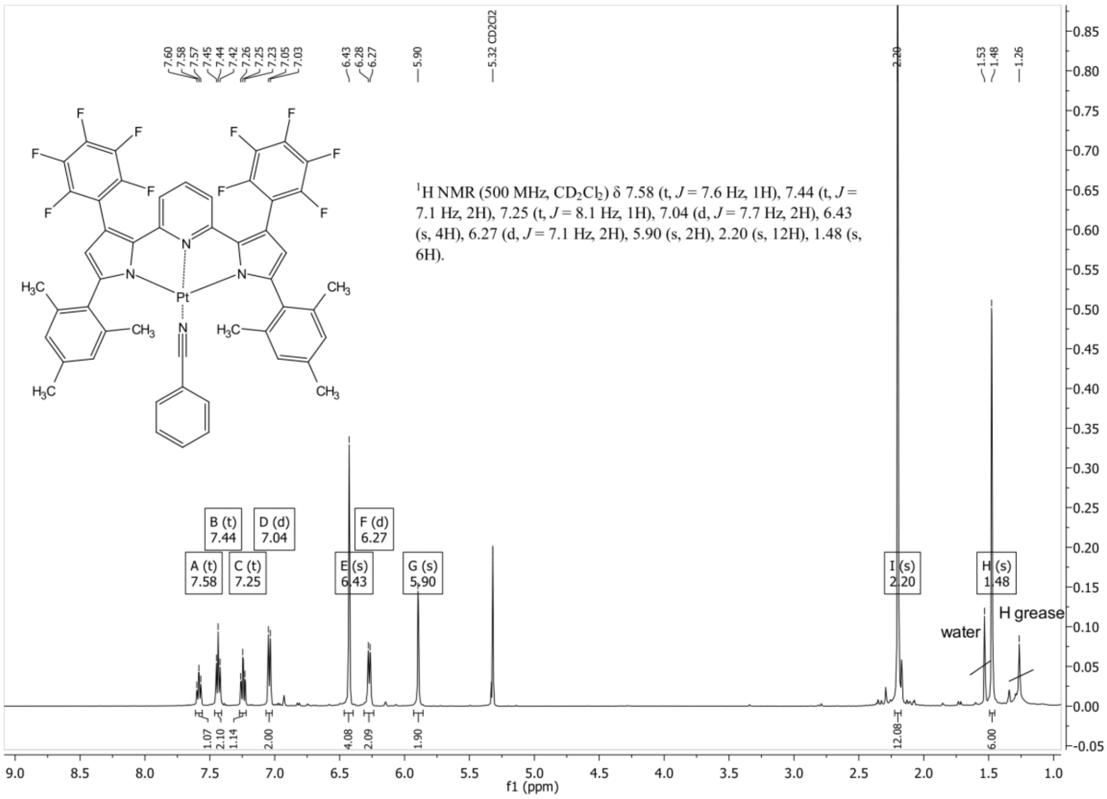

Figure S38. ^{1}H NMR spectrum of **Pt(^{Mes}PDP^{C6F5})(BN)** in CD_2Cl_2.

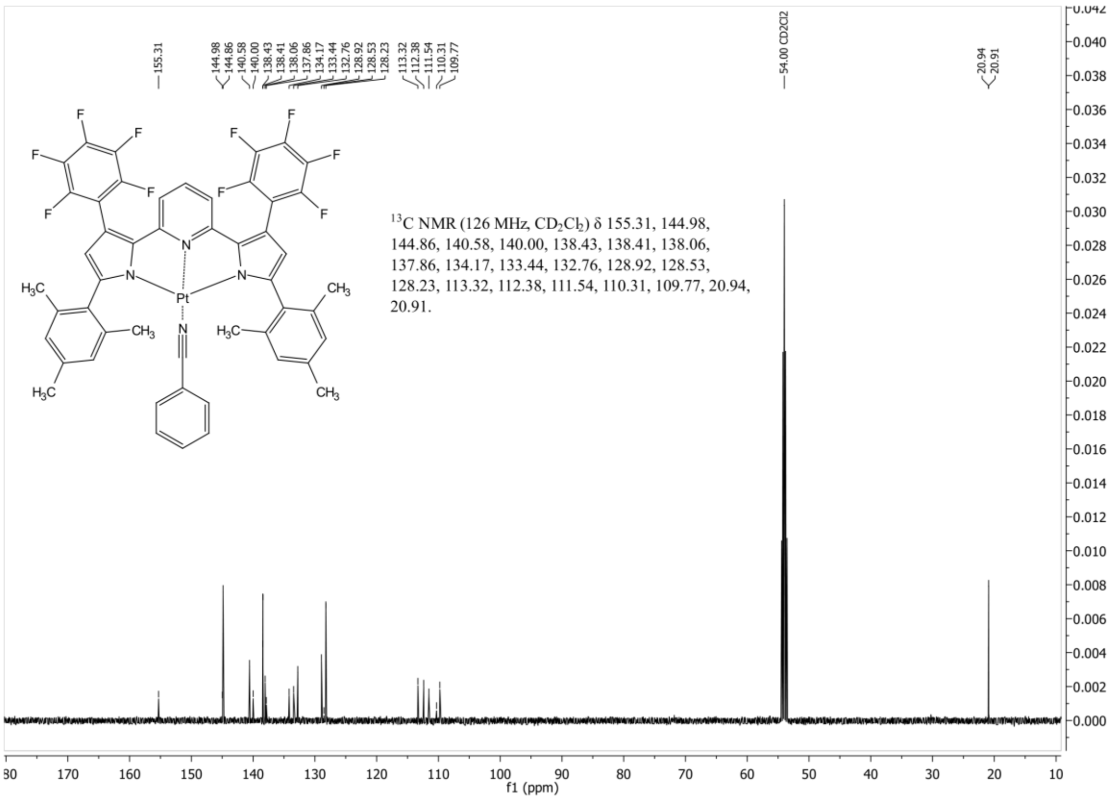

Figure S39. $^{13}C\{^{1}H\}\{^{19}F\}$ NMR spectrum of **Pt(^{Mes}PDP^{C6F5})(BN)** in CD_2Cl_2.

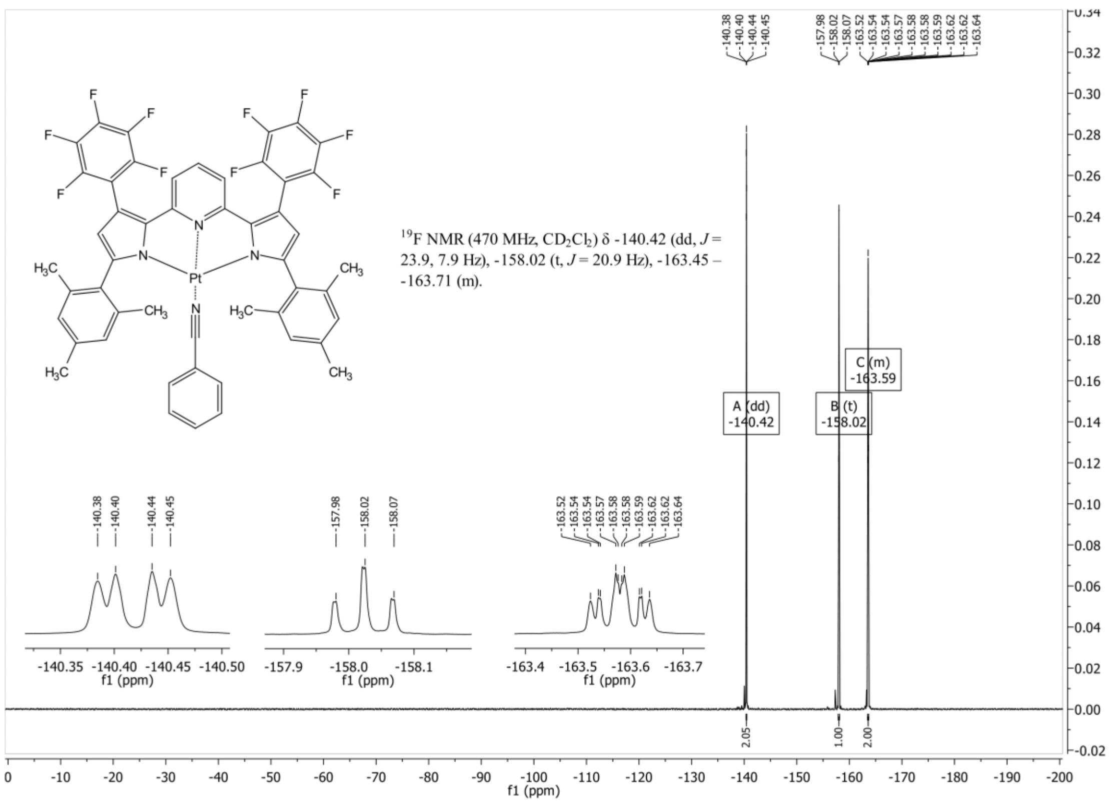

Figure S40. ^{19}F NMR spectrum of **Pt(^{Mes}PDP^{C6F5})(BN)** in CD_2Cl_2.

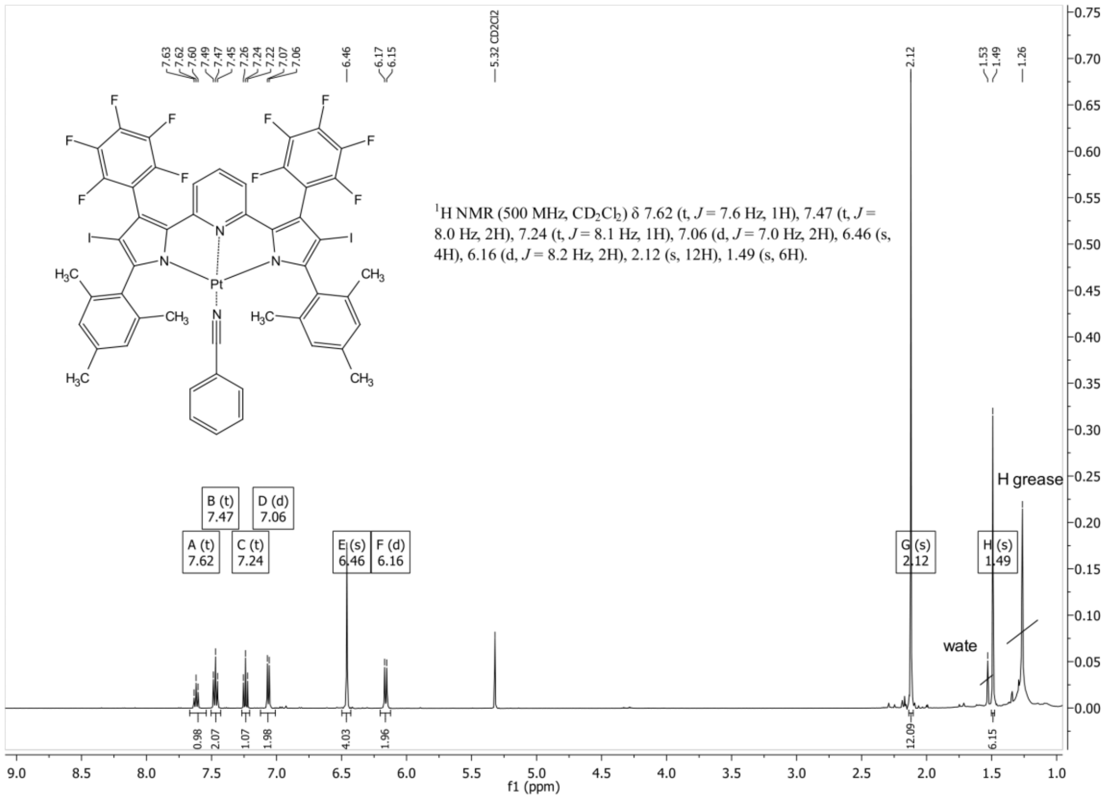

Figure S41. ^{1}H NMR spectrum of **Pt(^{Mes}IPDP^{C6F5})(BN)** in CD_2Cl_2.

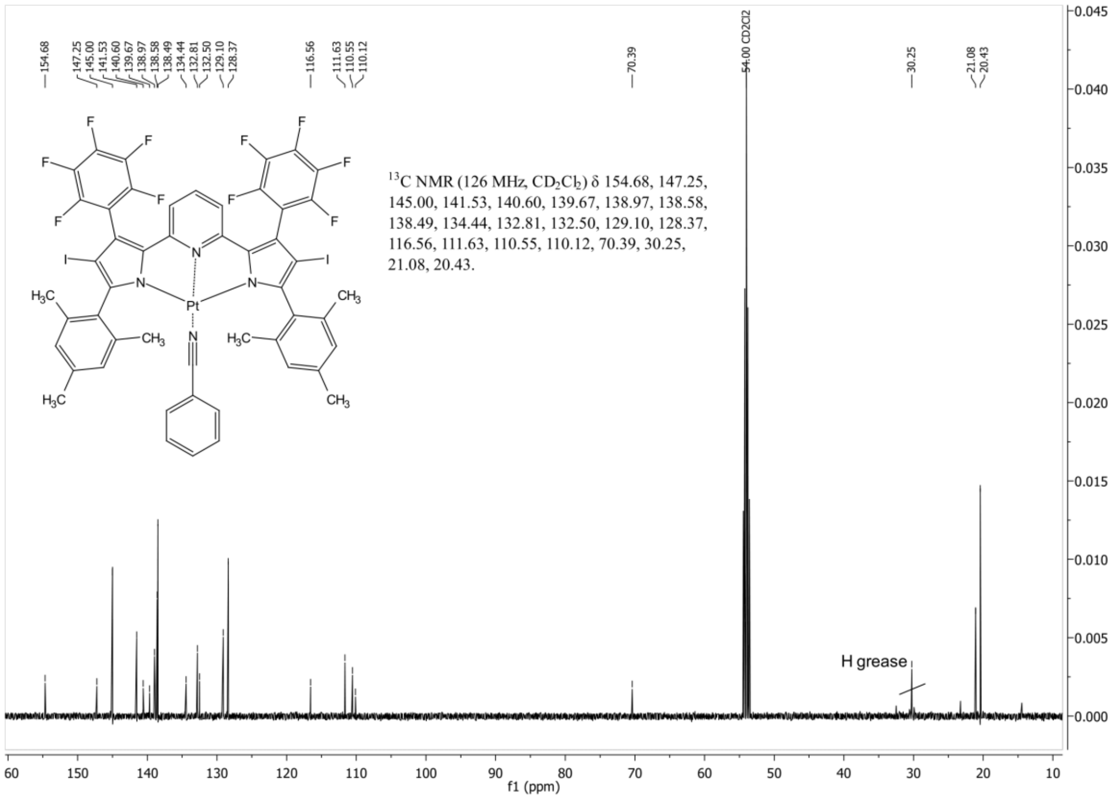

Figure S42. ^{13}C{^{1}H}{^{19}F} NMR spectrum of **Pt(^{Mes}IPDP^{C6F5})(BN)** in CD_2Cl_2.

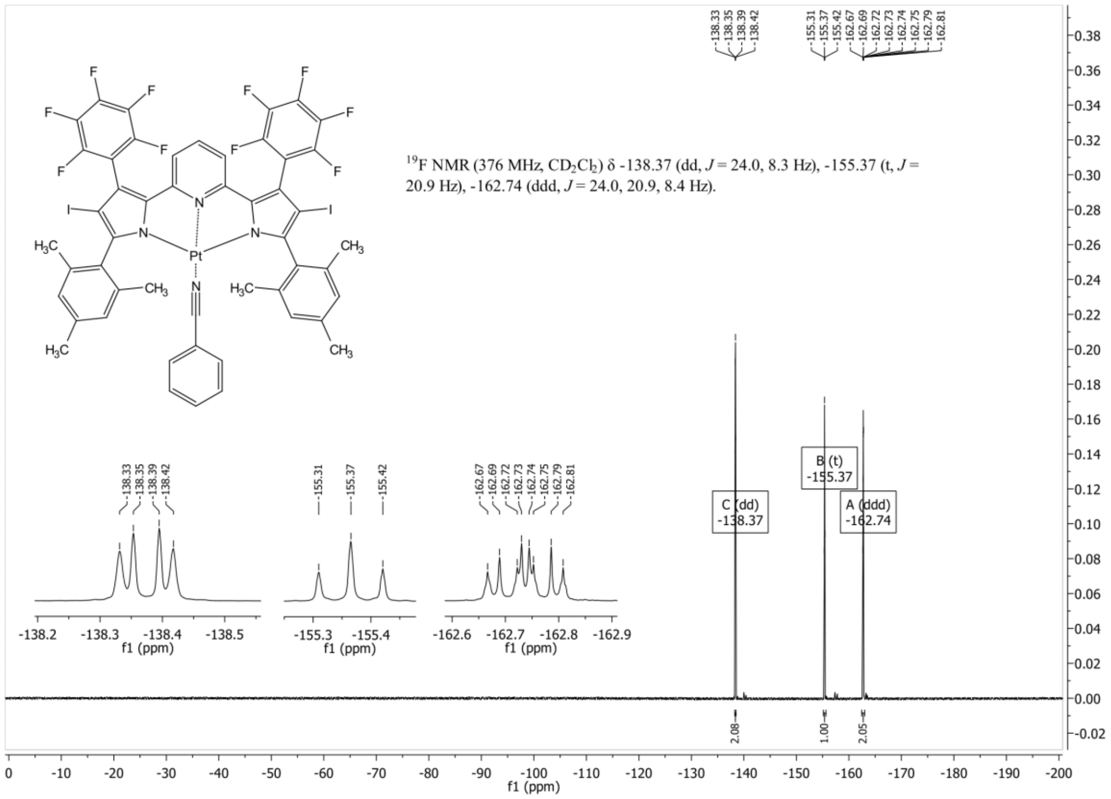

Figure S43. ^{19}F NMR spectrum of **Pt(^{Mes}IPDP^{C6F5})(BN)** in CD_2Cl_2.

6. Mass Spectra

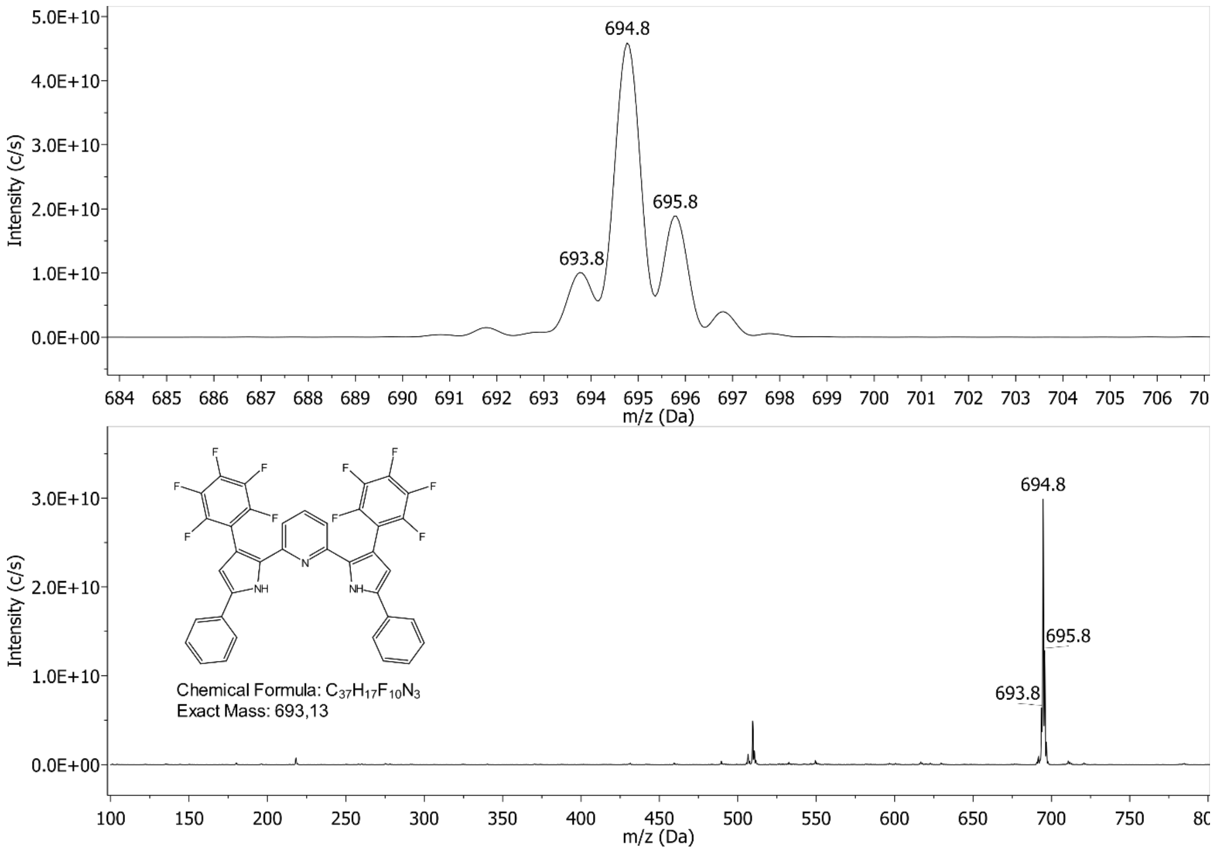

Figure S44. Mass spectrum (APCI-MS) of **H_2^{Ph}PDP^{C6F5}**.

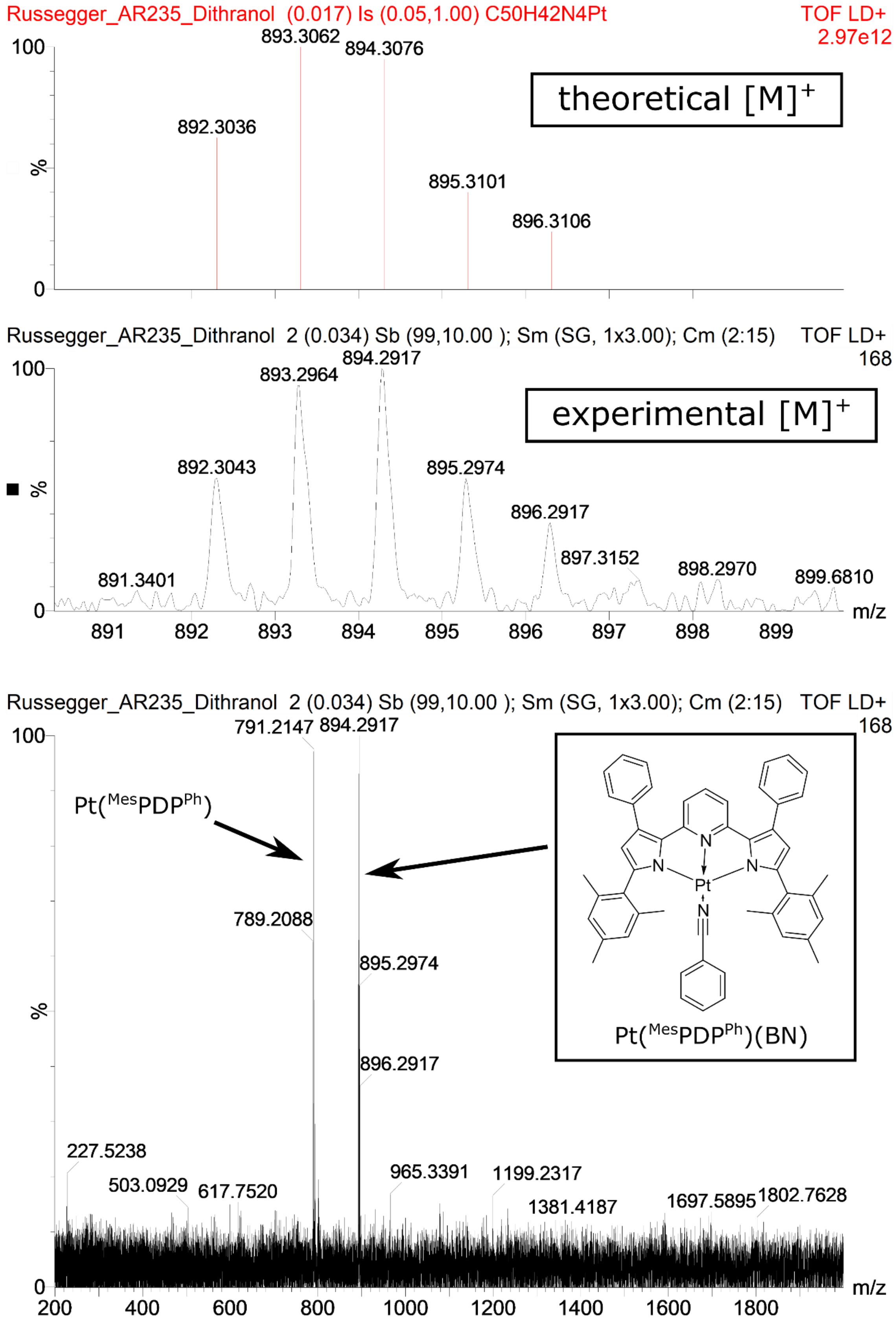

Figure S45. Mass spectrum (MALDI-TOF-MS) of **Pt(^{Mes}PDP^{Ph})(BN)** in dithranol matrix.

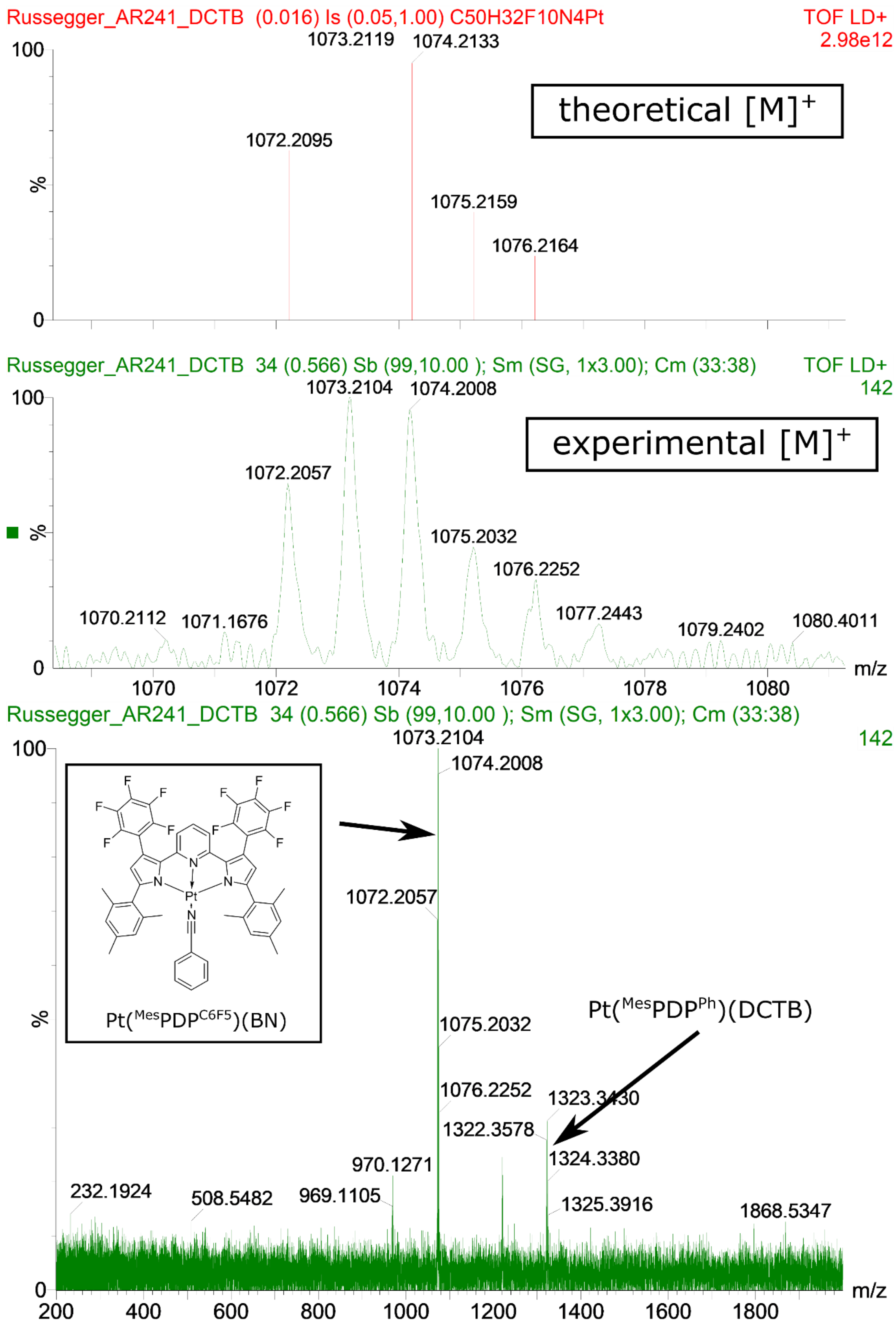

Figure S46. Mass spectrum (MALDI-TOF) of **Pt(^{Mes}PDP^{C6F5})(BN)** in DCTB matrix.

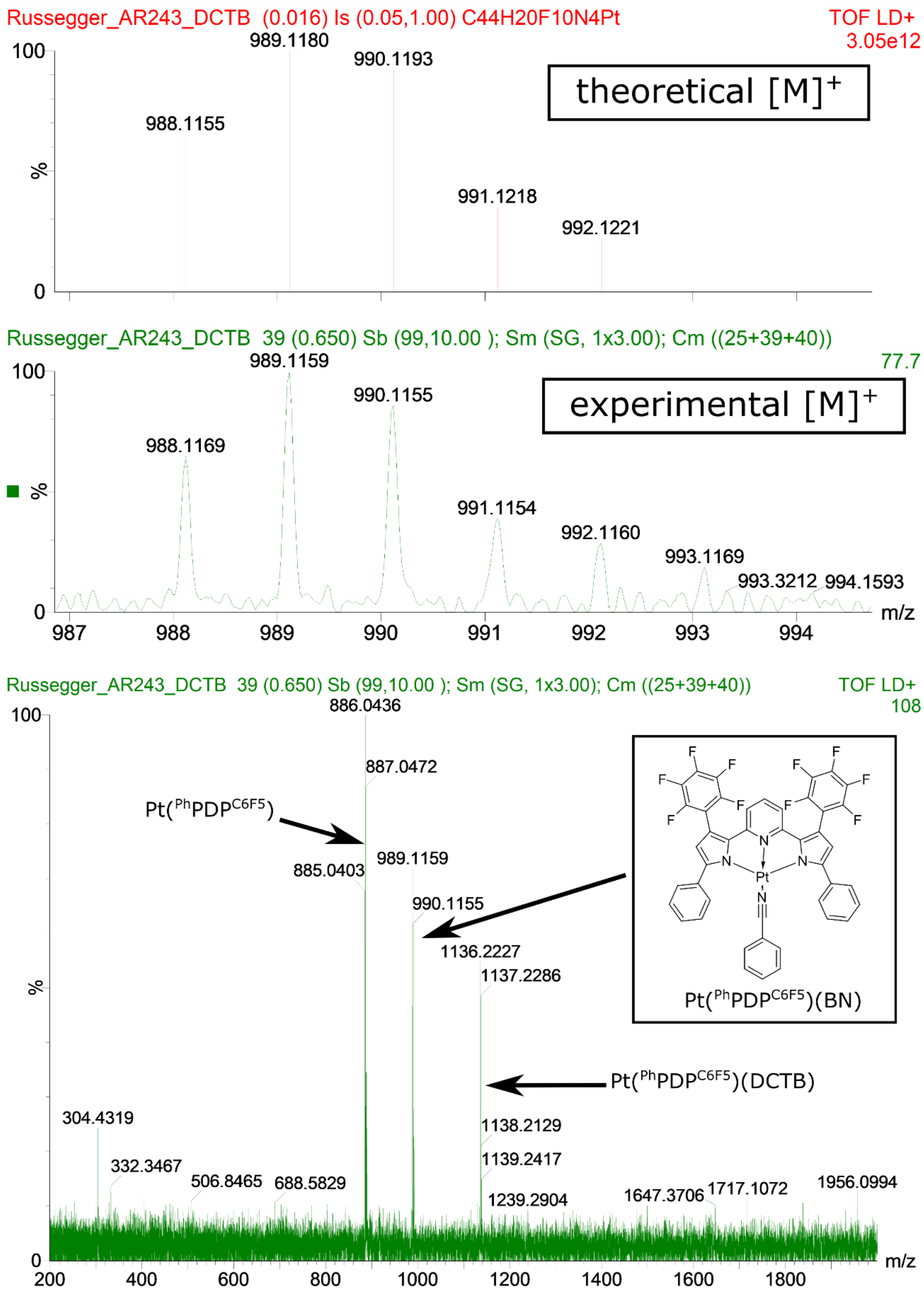

Figure S47. Mass spectrum (MALDI-TOF) of **Pt(^{Ph}PDP^{C6F5})(BN)** in DCTB matrix.

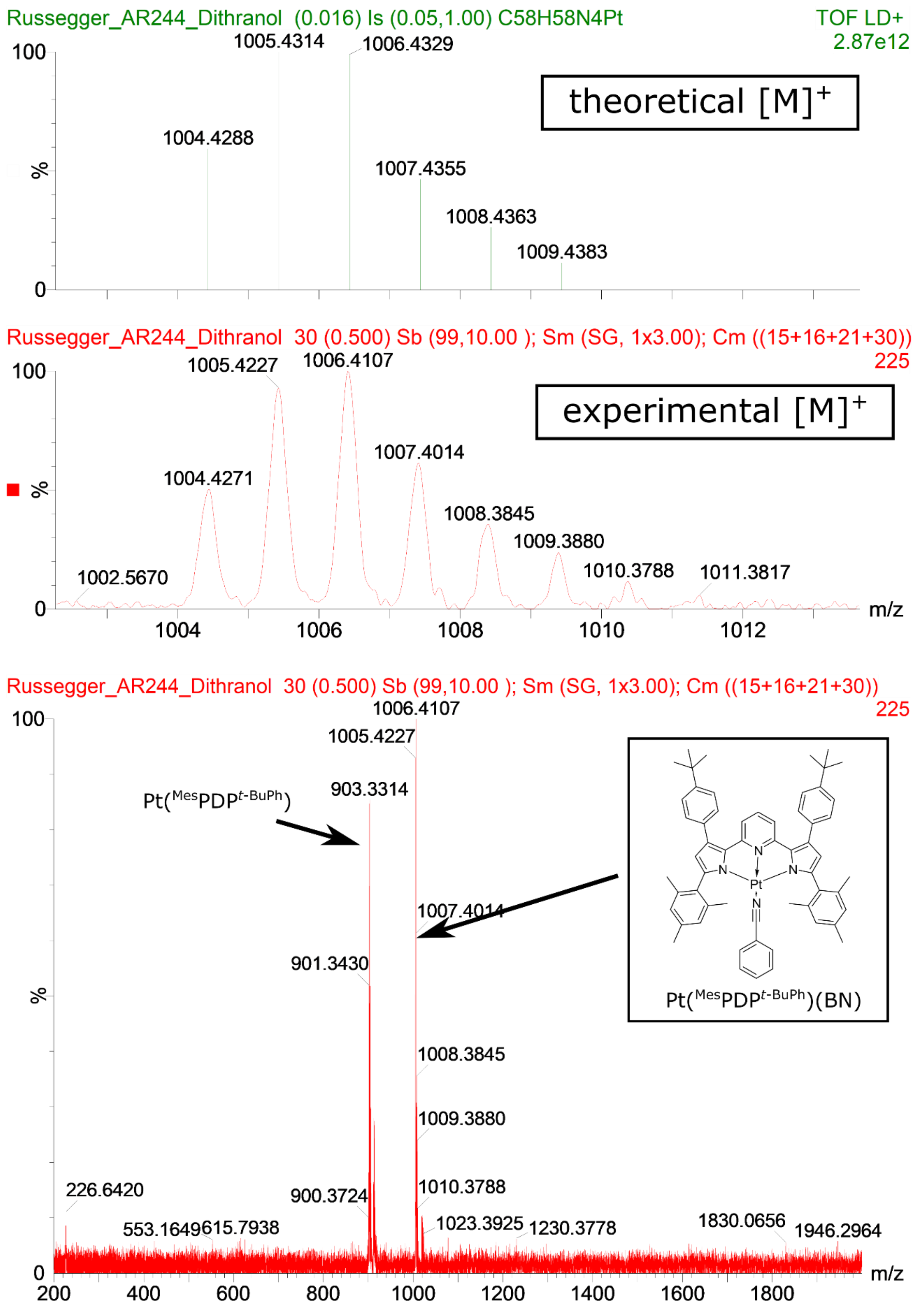

Figure S48. Mass spectrum (MALDI-TOF) of **Pt(^{Mes}PDP$^{t\text{-}BuPh}$)(BN)** in dithranol matrix.

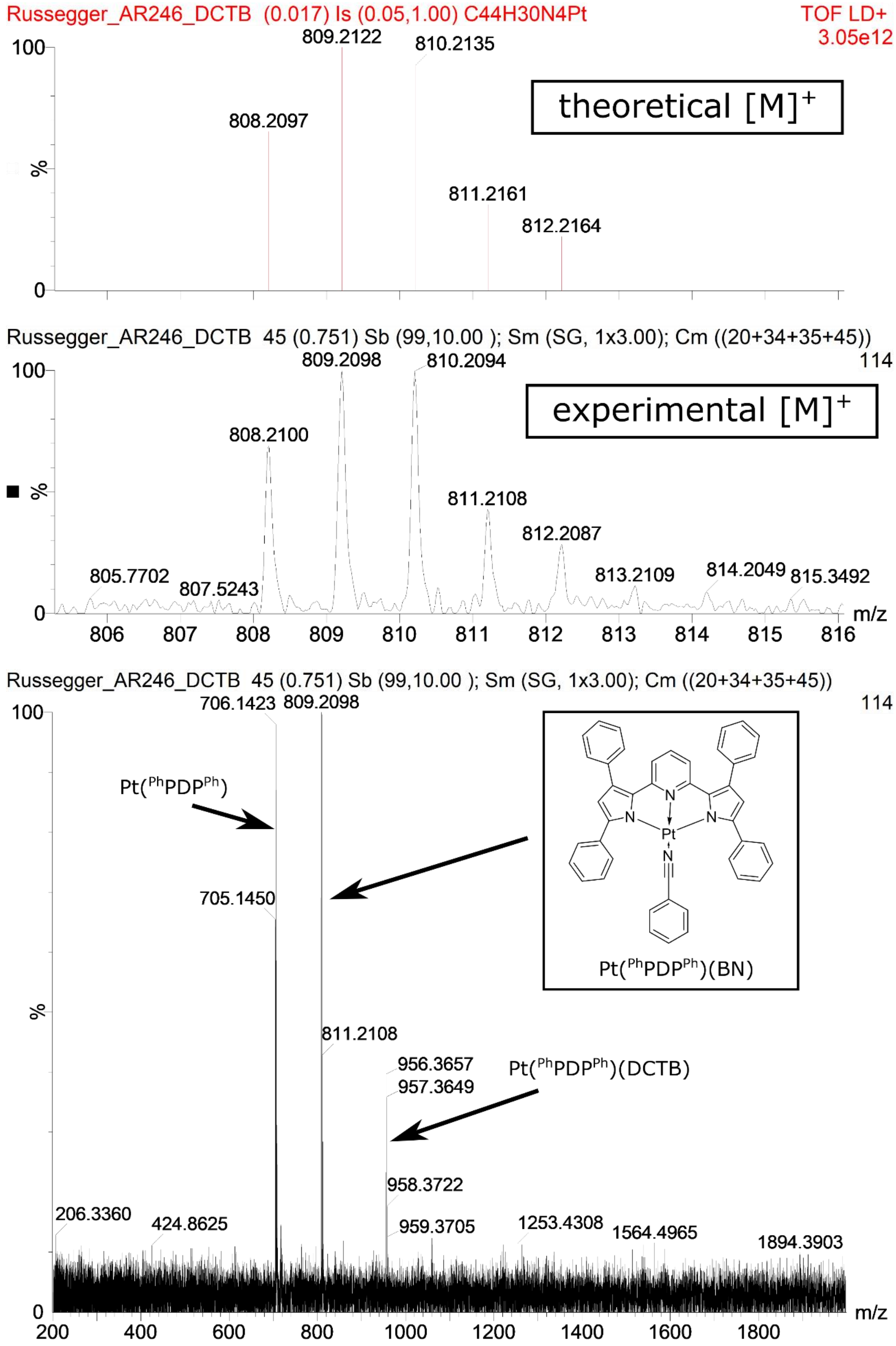

Figure S49. Mass spectrum (MALDI-TOF) of **Pt(^{Ph}PDP^{Ph})(BN)** in DCTB matrix.

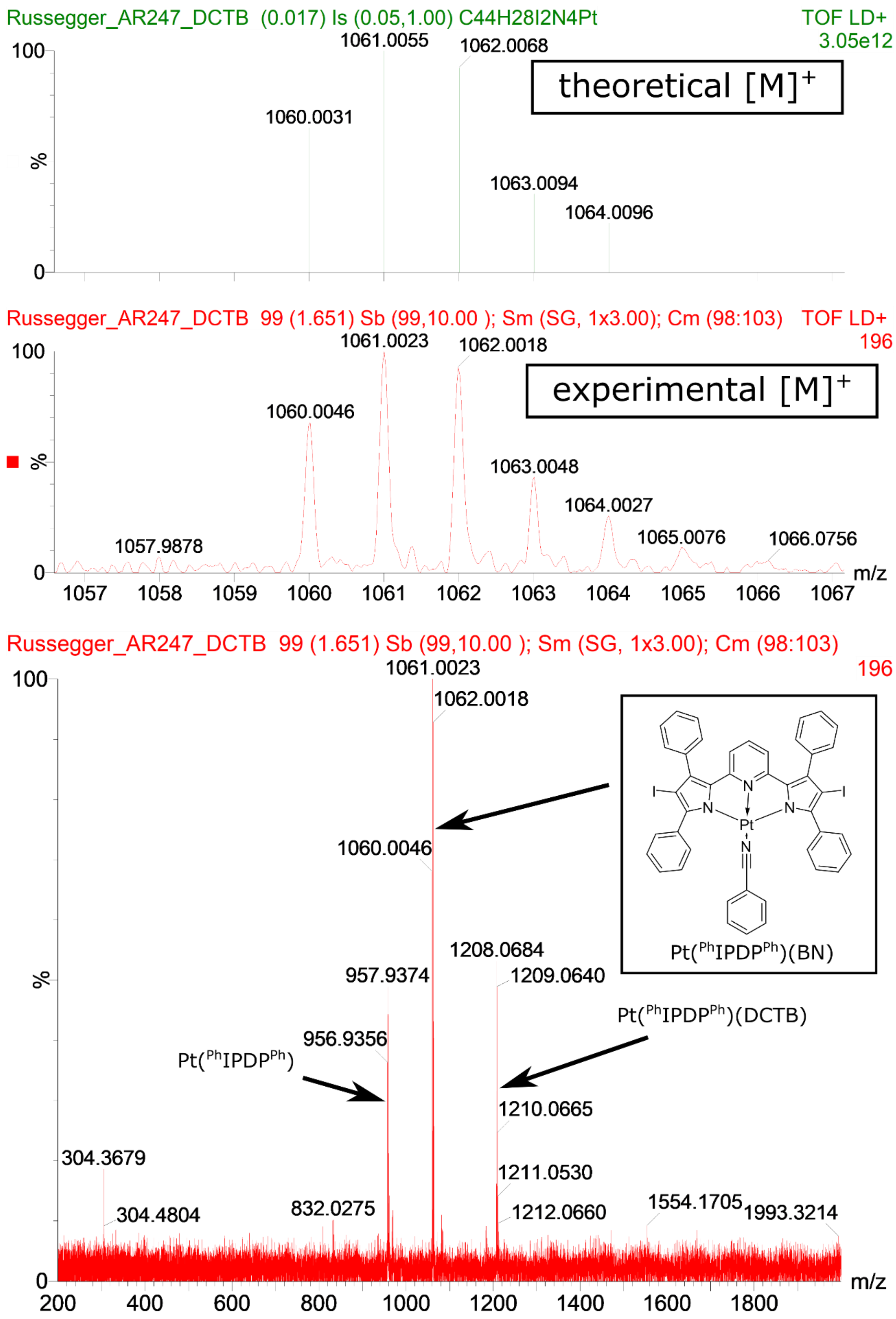

Figure S50. Mass spectrum (MALDI-TOF) of **Pt(^{Ph}IPDP^{Ph})(BN)** in DCTB matrix.

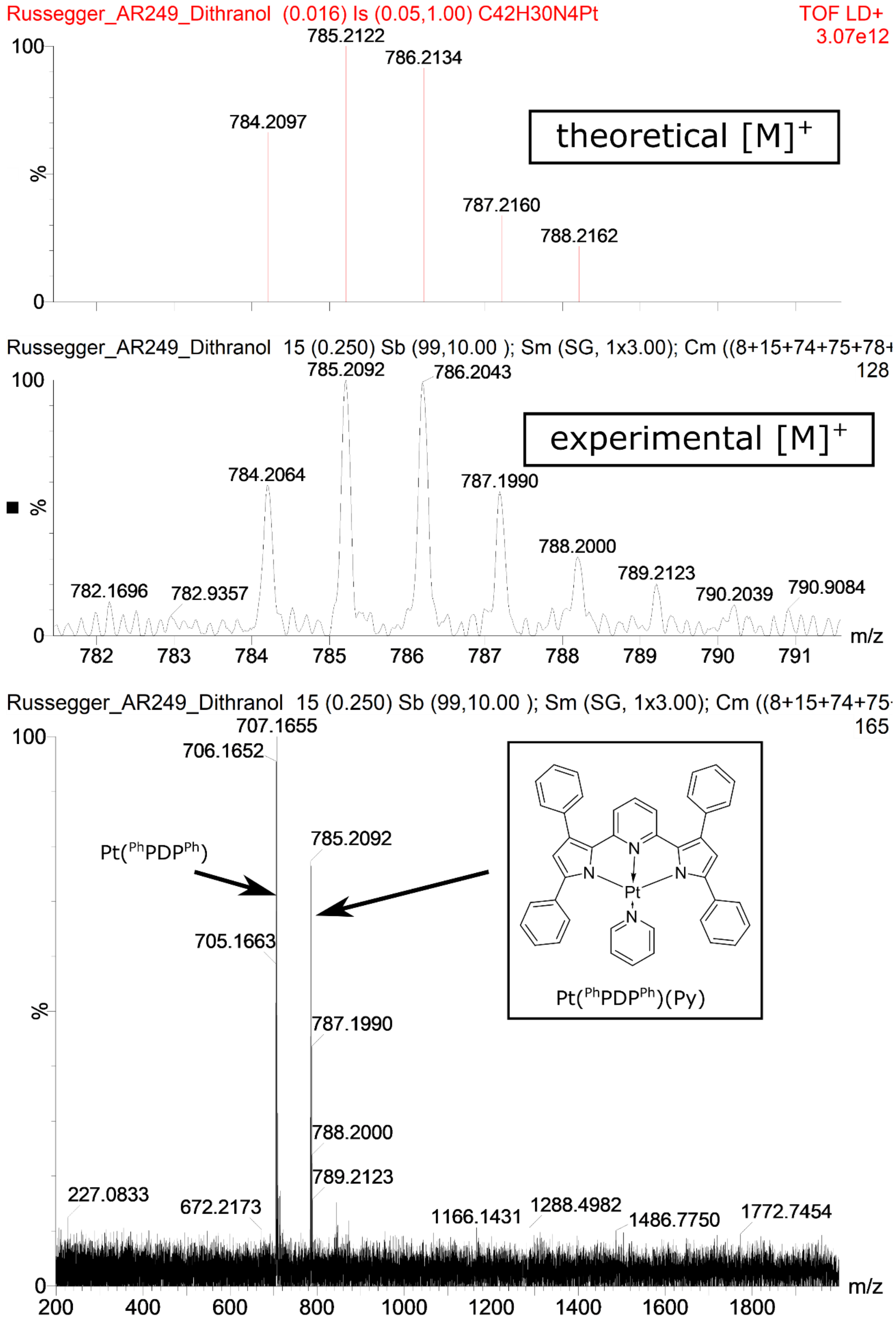

Figure S51. Mass spectrum (MALDI-TOF) of **Pt(^{Ph}PDP^{Ph})(Py)** in dithranol matrix.

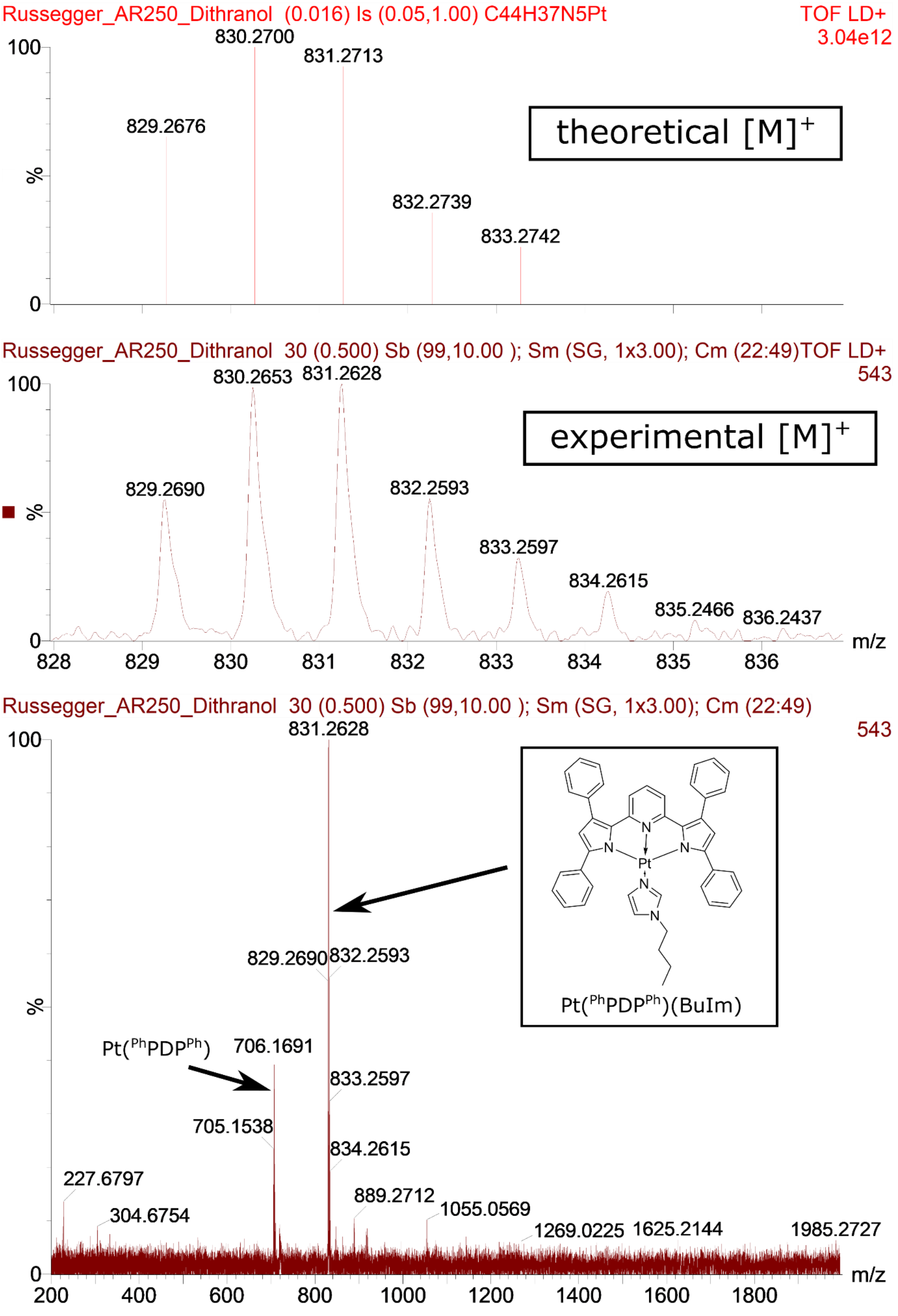

Figure S52. Mass spectrum (MALDI-TOF) of **Pt(^{Ph}PDP^{Ph})(BuIm)** in dithranol matrix.

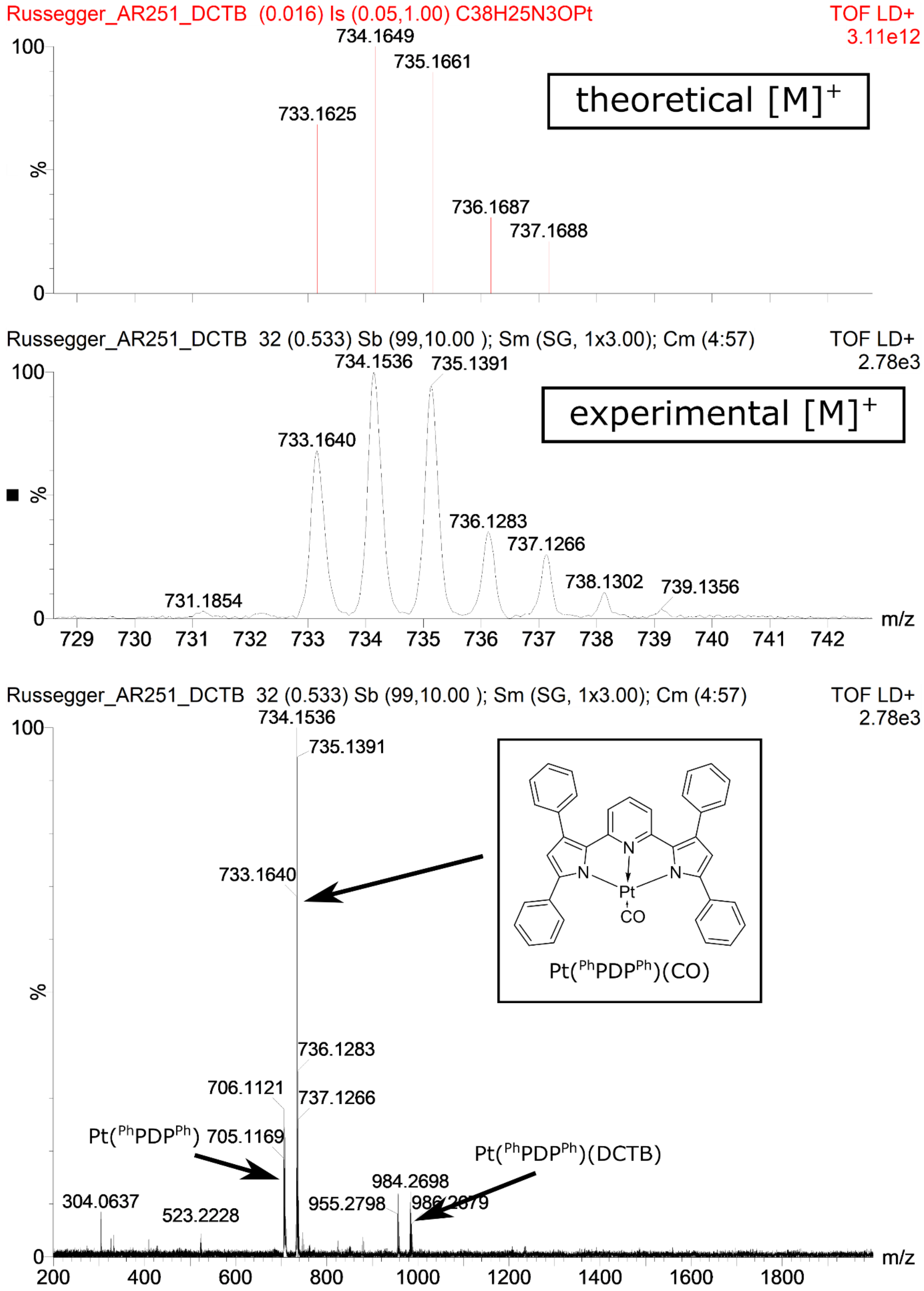

Figure S53. Mass spectrum (MALDI-TOF) of **Pt(^{Ph}PDP^{Ph})(CO)** in DCTB matrix.

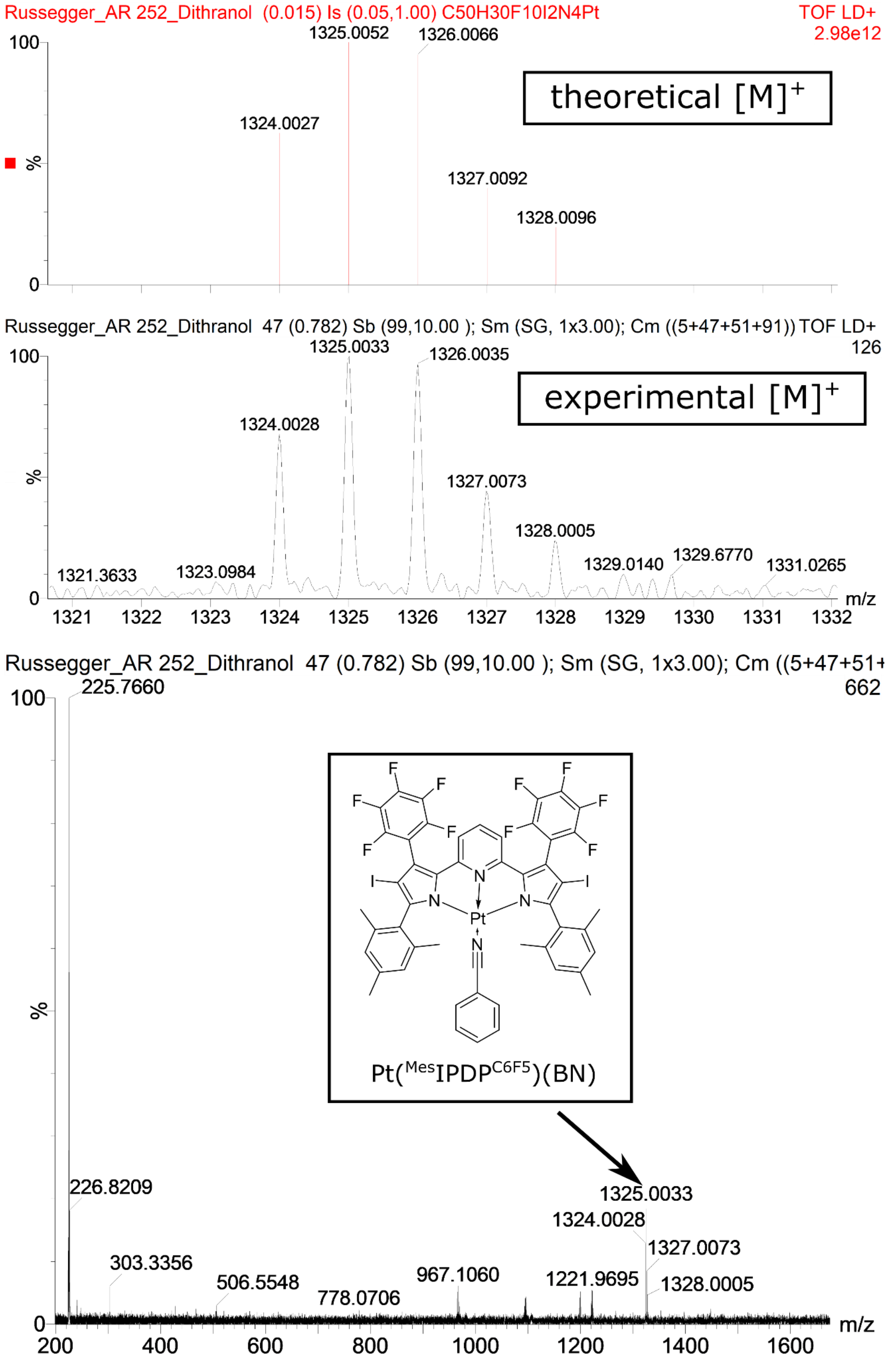

Figure S54. Mass spectrum (MALDI-TOF) of **Pt(^{Mes}IPDP^{C6F5})(BN)** in dithranol matrix.

7. References

(1) Neese, F. Software update: The ORCA Program System—Version 5.0. *Wiley Interdisciplinary Reviews: Computational Molecular Science* **2022**, *12* (5), e1606. DOI: 10.1002/wcms.1606.

(2) *Avogadro: an Open-Source Molecular Builder and Visualization Tool. Version 1.2.0.* http:// avogadro.cc/.

(3) Becke, A. D. Density‐Functional Thermochemistry. III. The Role of Exact Exchange. *J. Chem. Phys.* **1993**, *98* (7), 5648–5652. DOI: 10.1063/1.464913.

(4) Stephens, P. J.; Devlin, F. J.; Chabalowski, C. F.; Frisch, M. J. Ab Initio Calculation of Vibrational Absorption and Circular Dichroism Spectra Using Density Functional Force Fields. *J. Phys. Chem.* **1994**, *98* (45), 11623–11627. DOI: 10.1021/j100096a001.

(5) Weigend, F.; Ahlrichs, R. Balanced Basis Sets of Split Valence, Triple Zeta Valence and Quadruple Zeta Valence Quality for H to Rn: Design and Assessment of Accuracy. *Physical chemistry chemical physics : PCCP* **2005**, *7* (18), 3297–3305. DOI: 10.1039/B508541A.

(6) Weigend, F. Accurate Coulomb-Fitting Basis Sets for H to Rn. *Physical chemistry chemical physics : PCCP* **2006**, *8* (9), 1057–1065. DOI: 10.1039/B515623H.

(7) Grimme, S.; Antony, J.; Ehrlich, S.; Krieg, H. A Consistent and Accurate ab initio Parametrization of Density Functional Dispersion Correction (DFT-D) for the 94 Elements H-Pu. *The Journal of chemical physics* **2010**, *132* (15), 154104. DOI: 10.1063/1.3382344.

(8) Neese, F.; Wennmohs, F.; Hansen, A.; Becker, U. Efficient, Approximate and Parallel Hartree–Fock and Hybrid DFT Calculations. A 'Chain-of-Spheres' Algorithm for the Hartree–Fock Exchange. *Chemical Physics* **2009**, *356* (1-3), 98–109. DOI: 10.1016/j.chemphys.2008.10.036.

(9) Izsák, R.; Neese, F. An Overlap Fitted Chain of Spheres Exchange Method. *The Journal of chemical physics* **2011**, *135* (14), 144105. DOI: 10.1063/1.3646921.

(10) Barone, V.; Cossi, M. Quantum Calculation of Molecular Energies and Energy Gradients in Solution by a Conductor Solvent Model. *J. Phys. Chem. A* **1998**, *102* (11), 1995–2001. DOI: 10.1021/jp9716997.

(11) Younker, J. M.; Dobbs, K. D. Correlating Experimental Photophysical Properties of Iridium(III) Complexes to Spin–Orbit Coupled TDDFT Predictions. *19327455* **2013**, *117* (48), 25714–25723. DOI: 10.1021/jp410576a.

(12) Pander, P.; Zaytsev, A. V.; Sil, A.; Williams, J. A. G.; Lanoe, P.-H.; Kozhevnikov, V. N.; Dias, F. B. The Role of Dinuclearity in Promoting Thermally Activated Delayed Fluorescence (TADF) in Cyclometallated, N^C^N-Coordinated Platinum(II) Complexes. *J. Mater. Chem. C* **2021**, *9* (32), 10276–10287. DOI: 10.1039/D1TC02562G.

(13) Pander, P.; Zaytsev, A. V.; Sil, A.; Williams, J. A. G.; Kozhevnikov, V. N.; Dias, F. B. Enhancement of Thermally Activated Delayed Fluorescence Properties by Substitution of Ancillary Halogen in a Multiple Resonance-Like Diplatinum(II) Complex. *J. Mater. Chem. C* **2022**, *10* (12), 4851–4860. DOI: 10.1039/D1TC05026E.

(14) Zhang, Y.; Lee, T. S.; Favale, J. M.; Leary, D. C.; Petersen, J. L.; Scholes, G. D.; Castellano, F. N.; Milsmann, C. Delayed fluorescence from a Zirconium(IV) Photosensitizer with Ligand-to-Metal Charge-Transfer Excited States. *Nature Chemistry* **2020**, *12* (4), 345–352. DOI: 10.1038/s41557-020-0430-7.

(15) van Lenthe, E.; Baerends, E. J.; Snijders, J. G. Relativistic Regular Two‐Component Hamiltonians. *The Journal of chemical physics* **1993**, *99* (6), 4597–4610. DOI: 10.1063/1.466059.

(16) van Lenthe, E.; Baerends, E. J.; Snijders, J. G. Relativistic Total Energy Using Regular Approximations. *The Journal of chemical physics* **1994**, *101* (11), 9783–9792. DOI: 10.1063/1.467943.
